# Supplementary material for: Swimming exercise enhances brain plasticity in fish
Source: R Soc Open Sci. 2020 Jan 15;7(1):191640. doi: 10.1098/rsos.191640 (PMC7029906; doi:10.1098/rsos.191640)
Supplement: Supplementary figures and Tables [file rsos191640supp1.docx]

**Swimming exercise enhances brain plasticity in fish**

**Daan Mes^a^, Arjan P. Palstra^b^, Christiaan V. Henkel^c^, Ian Mayer^a^, Marco A. Vindas^d,e^***

* Corresponding author (marco.vindas@nmbu.no)

**Supplementary information**

**Table S1. Primer sequences for target genes.**

| Gene | Primer Sequence 5’ 🡪 3’ | Accession no. | Reference |
| --- | --- | --- | --- |
| *ef1αa* | Fw CCCCTCCAGGACGTTTACAAA  Rev CACACGGCCCACAGGTACA | BT059133.1 | Ingerslev et al. 2006. *Molec Immunol* 43(8): 1194-1201 |
| *S20* | Fwd GCAGACCTTATCCGTGGAGCTA  Rev TGGTGATGCGCAGAGTCTTG | NM_001140843.1 | Olsvik et al. 2005. *BMC* *Molec Biol* 6(1): 21 |
| *hprt1* | Fwd CGTGGCTCTCTGCGTGCTCA  Rev TGGAGCGGTCGCTGTTACGG | BT043501.1 | Andreassen et al. 2009 *BMC* *Genom* 10: 502 |
| *bdnf* | Fwd ATGTCTGGGCAGACCGTTAC  Rev GTTGTCCTGCATTGGGAGTT | GU108576.1 | Vindas et al. 2007. *J Exp Biol* 220(8): 1524-1532 |
| *syt* | Fwd GTTCAGCTTCAGAGTCCCCC  Rev GGAGAACTGGCCAATCACGA | XM_014194413.1 | This study |
| *pcna* | Fwd TGAGCTCGTCGGGTATCTCT  Rev CTCGAAGACTAGGGCGAGTG | BT056931.1 | Vindas et al. 2007. *J Exp Biol* 220(8): 1524-1532 |
| *neurod* | Fwd CAATGGACAGCTCCCACATCT  Rev CCAGCGCACTTCCGTATGA | BT058820.1 | Vindas et al. 2007. *J Exp Biol* 220(8): 1524-1532 |

**Table S2. qPCR validation.**

|  | *s20* | *ef1αa* | *hrpt1* | *bdnf* | *syt* | *pcna* | *neurod* |
| --- | --- | --- | --- | --- | --- | --- | --- |
| Slope | -2.6461 | -3.0560 | -3.0863 | -3.2359 | -3.0583 | -2.8474 | -3.1074 |
| Efficiency | 2.39 | 2.12 | 2.11 | 2.04 | 2.12 | 2.24 | 2.10 |
| Error | 0.36 | 0.15 | 0.23 | 0.23 | 0.44 | 0.46 | 0.53 |
| R^2 | 0.95 | 0.99 | 0.99 | 0.98 | 0.95 | 0.94 | 0.93 |
| Y-Intercept | 24.81 | 24.00 | 27.16 | 27.26 | 26.68 | 28.85 | 30.73 |

**Table S3.** Upregulated (false discovery rate (FDR) < 0.01) genes in Atlantic salmon juveniles subjected to 8 weeks of exercise, compared to unexercised controls, expressed as fold change (FC) differences.

| Gene name | Transcript ID | Product | FC |
| --- | --- | --- | --- |
| LOC106584782 | XM_014170341.1 | mitochondrial glutamate carrier 1-like | 4.28 |
| LOC106613326 | XM_014215426.1 | beta-arrestin-1 | 4.19 |
| LOC106564442 | XM_014130543.1 | protein phosphatase 1 regulatory subunit 29-like | 4.05 |
| LOC106563916 | XM_014129862.1 | metabotropic glutamate receptor 5-like | 3.89 |
| LOC106590004 | XM_014180484.1 | AT-rich interactive domain-containing protein 4B-like | 3.68 |
| LOC106592276 | XM_014183599.1 | protein unc-80 homolog | 3.50 |
| LOC106611460 | XM_014211674.1 | E3 ubiquitin-protein ligase TRIM9-like | 3.41 |
| LOC106589110 | XM_014178806.1 | disks large-associated protein 3-like | 3.36 |
| LOC106610165 | XM_014209350.1 | extensin-1-like | 3.28 |
| LOC106611028 | XM_014210842.1 | endothelial PAS domain-containing protein 1-like | 3.16 |
| LOC106582526 | XM_014165704.1 | fibronectin-like | 3.16 |
| synj1 | XM_014129816.1 | synaptojanin 1 | 3.15 |
| LOC106592751 | XM_014184076.1 | synaptotagmin-7-like | 3.11 |
| LOC106566150 | XM_014134005.1 | kinesin heavy chain isoform 5A-like | 3.10 |
| LOC106582460 | XM_014165597.1 | disintegrin and metalloproteinase domain-containing protein 23-like | 3.10 |
| LOC106563942 | XM_014129897.1 | phosphatidylinositol transfer protein alpha isoform-like | 3.05 |
| adcyap1r1 | XM_014123308.1 | adenylate cyclase activating polypeptide 1 (pituitary) receptor type I | 2.96 |
| LOC106570910 | XM_014143493.1 | sodium-dependent phosphate transporter 2-like | 2.86 |
| LOC106574207 | XM_014149879.1 | dermatopontin-like | 2.84 |
| LOC106579568 | XM_014159606.1 | ranBP-type and C3HC4-type zinc finger-containing protein 1-like | 2.74 |
| LOC106609628 | XM_014208583.1 | protein piccolo-like | 2.74 |
| LOC106592062 | XM_014183365.1 | caskin-1-like | 2.73 |
| LOC106589999 | XM_014180480.1 | AT-rich interactive domain-containing protein 4B-like | 2.71 |
| LOC106573403 | XM_014148415.1 | SH3 and multiple ankyrin repeat domains protein 3-like | 2.70 |
| LOC106585963 | XM_014172714.1 | poly [ADP-ribose] polymerase 8-like | 2.69 |
| nrsn1 | XM_014181953.1 | neurensin 1 | 2.67 |
| LOC106572978 | XM_014147574.1 | neural cell adhesion molecule L1.1-like | 2.67 |
| LOC106569583 | XM_014141079.1 | cell adhesion molecule 3-like | 2.63 |
| LOC106586919 | XM_014174667.1 | myc box-dependent-interacting protein 1-like | 2.61 |
| LOC106590676 | XM_014181834.1 | cysteine-rich secretory protein LCCL domain-containing 1-like | 2.61 |
| LOC106605188 | XM_014200603.1 | protein EFR3 homolog B-like | 2.60 |
| LOC106608344 | XM_014206235.1 | uncharacterized LOC106608344 | 2.59 |
| LOC106583499 | XM_014167743.1 | plasma membrane calcium-transporting ATPase 1-like | 2.58 |
| LOC106576423 | XM_014153600.1 | muscarinic acetylcholine receptor M2-like | 2.56 |
| LOC106569760 | XM_014141350.1 | rho GTPase-activating protein 29-like | 2.55 |
| LOC106609497 | XM_014208374.1 | muscarinic acetylcholine receptor M2-like | 2.54 |
| LOC106566945 | XM_014135621.1 | phosphatidylinositol 3%2C4%2C5-trisphosphate-dependent Rac exchanger 1 protein-like | 2.54 |
| LOC106581415 | XM_014163473.1 | metabotropic glutamate receptor 5-like | 2.53 |
| LOC106570995 | XM_014143612.1 | kinesin-like protein KIF3B | 2.52 |
| herc1 | XM_014153804.1 | HECT and RLD domain containing E3 ubiquitin protein ligase family member 1 | 2.48 |
| LOC106567667 | XM_014137220.1 | protein phosphatase 1E-like | 2.43 |
| chst12 | XM_014186112.1 | carbohydrate (chondroitin 4) sulfotransferase 12 | 2.41 |
| LOC106608754 | XM_014206871.1 | vigilin-like | 2.39 |
| pdcl | XM_014123682.1 | phosducin-like | 2.39 |
| LOC106595266 | XM_014186640.1 | glutamate receptor 2-like | 2.38 |
| LOC106562493 | XM_014127402.1 | microtubule-associated protein 1A-like | 2.38 |
| igf1r | XM_014124119.1 | insulin-like growth factor 1 receptor | 2.38 |
| LOC106601049 | XM_014192927.1 | cAMP-specific 3'%2C5'-cyclic phosphodiesterase 4D-like | 2.37 |
| LOC106573449 | XM_014148501.1 | protein piccolo-like | 2.37 |
| LOC106595230 | XM_014186610.1 | neural cell adhesion molecule L1-like | 2.35 |
| LOC106566388 | XM_014134363.1 | sodium-dependent neutral amino acid transporter SLC6A17-like | 2.34 |
| LOC106589899 | XM_014180316.1 | serine/threonine-protein kinase LMTK1-like | 2.34 |
| LOC106611901 | XM_014212556.1 | janus kinase and microtubule-interacting protein 1-like | 2.34 |
| vgll4 | XM_014168352.1 | vestigial-like family member 4 | 2.34 |
| LOC106572946 | XM_014147537.1 | IQ motif and SEC7 domain-containing protein 2-like | 2.34 |
| LOC106560380 | XM_014123252.1 | rho GTPase-activating protein 39-like | 2.33 |
| LOC106604362 | XM_014198918.1 | monocarboxylate transporter 8-like | 2.32 |
| LOC106568697 | XM_014139263.1 | abl interactor 1-like | 2.32 |
| LOC106567148 | XM_014136111.1 | basement membrane-specific heparan sulfate proteoglycan core protein-like | 2.32 |
| LOC106560445 | XM_014123362.1 | breast cancer metastasis-suppressor 1 homolog | 2.30 |
| LOC106606938 | XM_014203427.1 | glycine receptor subunit beta-like | 2.30 |
| slc6a11 | XM_014168361.1 | solute carrier family 6 (neurotransmitter transporter)%2C member 11 | 2.30 |
| LOC106576213 | XM_014153216.1 | voltage-dependent P/Q-type calcium channel subunit alpha-1A-like | 2.30 |
| LOC106603846 | XM_014198033.1 | gamma-aminobutyric acid receptor subunit beta-2-like | 2.26 |
| cssa09h20orf194 | XM_014211656.1 | chromosome ssa09 open reading frame%2C human C20orf194 | 2.25 |
| LOC106578942 | XM_014158231.1 | neurexin-1a-like | 2.25 |
| LOC106565592 | XM_014132881.1 | disco-interacting protein 2 homolog B-A-like | 2.25 |
| hnrnpul1 | XM_014215521.1 | heterogeneous nuclear ribonucleoprotein U-like 1 | 2.25 |
| LOC106581527 | XM_014163631.1 | ubiquitin carboxyl-terminal hydrolase 32-like | 2.25 |
| LOC106570681 | XM_014143154.1 | gamma-aminobutyric acid type B receptor subunit 1-like | 2.24 |
| LOC106578054 | XM_014156620.1 | NHS-like protein 2 | 2.24 |
| LOC106593073 | XM_014184416.1 | partner and localizer of BRCA2-like | 2.24 |
| LOC106613305 | XM_014215393.1 | neprilysin-like | 2.23 |
| LOC106570801 | XM_014143337.1 | calcium/calmodulin-dependent protein kinase type II subunit beta-like | 2.23 |
| LOC106573868 | XM_014149278.1 | probable cation-transporting ATPase 13A3 | 2.23 |
| LOC106592932 | XM_014184288.1 | PHD finger protein 21B-like | 2.21 |
| LOC106607452 | XM_014204412.1 | synaptogyrin-1-like | 2.21 |
| LOC106563878 | XM_014129802.1 | tripartite motif-containing protein 3-like | 2.20 |
| LOC106612202 | XM_014213167.1 | leucine-rich repeat extensin-like protein 5 | 2.20 |
| LOC106563893 | XM_014129830.1 | splicing factor%2C arginine/serine-rich 15-like | 2.19 |
| LOC106605517 | XM_014201257.1 | sodium- and chloride-dependent transporter XTRP3-like | 2.17 |
| LOC106586624 | XM_014174111.1 | immunoglobulin superfamily member 3-like | 2.17 |
| LOC106561392 | XM_014125320.1 | homeodomain-interacting protein kinase 2-like | 2.17 |
| LOC106605888 | XM_014201881.1 | uncharacterized protein DDB_G0284459-like | 2.16 |
| LOC106591965 | XM_014183239.1 | serine/threonine-protein kinase DCLK1-like | 2.15 |
| LOC106571967 | XM_014145585.1 | death-inducer obliterator 1-like | 2.15 |
| LOC106611279 | XM_014211318.1 | tau-tubulin kinase 2-like | 2.15 |
| LOC106579599 | XM_014159658.1 | histone-lysine N-methyltransferase 2C-like | 2.14 |
| LOC106610406 | XM_014209771.1 | circadian locomoter output cycles protein kaput-like | 2.14 |
| LOC106574702 | XM_014150714.1 | striated muscle preferentially expressed protein kinase-like | 2.13 |
| LOC106561741 | XM_014125968.1 | pleckstrin homology domain-containing family A member 5-like | 2.13 |
| LOC106582632 | XM_014165868.1 | chromodomain-helicase-DNA-binding protein 6-like | 2.13 |
| LOC106572123 | XM_014145993.1 | WD repeat-containing protein 7-like | 2.12 |
| LOC106588753 | XM_014178093.1 | F-box/LRR-repeat protein 2-like | 2.12 |
| LOC106572850 | XM_014147383.1 | monocarboxylate transporter 5-like | 2.12 |
| LOC106612075 | XM_014212904.1 | ATP-binding cassette sub-family A member 5-like | 2.12 |
| LOC106609808 | XM_014208831.1 | probable E3 ubiquitin-protein ligase makorin-1 | 2.11 |
| LOC106580977 | XM_014162587.1 | neurobeachin-like | 2.11 |
| pvrl1 | NM_001146661.1 | Poliovirus receptor-related protein 1 | 2.11 |
| LOC106563271 | XM_014128714.1 | zinc finger protein 618-like | 2.11 |
| LOC106594246 | XM_014185622.1 | trinucleotide repeat-containing gene 18 protein-like | 2.11 |
| LOC106600127 | XM_014191463.1 | tensin-1-like | 2.11 |
| LOC106562976 | XM_014128136.1 | disabled homolog 2-interacting protein-like | 2.10 |
| tmem55a | XM_014178844.1 | transmembrane protein 55A | 2.10 |
| LOC106591642 | XM_014182848.1 | fascin-like | 2.10 |
| LOC106580646 | XM_014161925.1 | leucine-rich repeat and calponin homology domain-containing protein 3-like | 2.09 |
| LOC106583361 | XM_014167493.1 | ERC protein 2-like | 2.09 |
| LOC106573711 | XM_014149012.1 | CMP-N-acetylneuraminate-beta-galactosamide-alpha-2%2C3-sialyltransferase 2-like | 2.09 |
| LOC106603714 | XM_014197733.1 | calpain-5-like | 2.09 |
| cdk19 | XM_014144231.1 | cyclin-dependent kinase 19 | 2.09 |
| LOC106580423 | XM_014161465.1 | clathrin heavy chain 1-like | 2.09 |
| LOC106572920 | XM_014147506.1 | methyl-CpG-binding protein 2-like | 2.09 |
| LOC106580500 | XM_014161647.1 | phosphatidate cytidylyltransferase 2-like | 2.08 |
| LOC106572669 | XM_014147046.1 | histone-lysine N-methyltransferase 2D-like | 2.08 |
| LOC106595370 | XM_014186743.1 | fascin-like | 2.07 |
| sergef | XM_014126054.1 | secretion regulating guanine nucleotide exchange factor | 2.07 |
| LOC106613358 | XM_014215481.1 | ubiquitin carboxyl-terminal hydrolase 32-like | 2.07 |
| LOC106572749 | XM_014147193.1 | tensin-2-like | 2.07 |
| LOC106575448 | XM_014151948.1 | mucin-17-like | 2.07 |
| LOC106585059 | XM_014170819.1 | clathrin heavy chain 1-like | 2.06 |
| LOC106578744 | XM_014157877.1 | lysosomal-trafficking regulator-like | 2.06 |
| LOC106569883 | XM_014141596.1 | synaptotagmin-11-like | 2.06 |
| LOC106566514 | XM_014134609.1 | ankyrin-1-like | 2.06 |
| LOC106595312 | XM_014186688.1 | putative ubiquitin carboxyl-terminal hydrolase 50 | 2.06 |
| LOC106565257 | XM_014132162.1 | plexin-B1-like | 2.05 |
| LOC106608262 | XM_014206116.1 | synapse differentiation-inducing gene protein 1-like | 2.05 |
| LOC106570191 | XM_014142238.1 | ras/Rap GTPase-activating protein SynGAP-like | 2.05 |
| LOC106601156 | XM_014193116.1 | glutamate receptor ionotropic%2C NMDA 2B-like | 2.05 |
| LOC106562562 | XM_014127503.1 | uncharacterized protein KIAA0895-like | 2.05 |
| LOC106582115 | XM_014164868.1 | nectin-4-like | 2.05 |
| LOC106610931 | XM_014210616.1 | gamma-aminobutyric acid receptor subunit gamma-1-like | 2.04 |
| LOC106611402 | XM_014211572.1 | protein Jade-1-like | 2.03 |
| LOC106569696 | XM_014141259.1 | receptor-type tyrosine-protein phosphatase F-like | 2.03 |
| LOC106609939 | XM_014208975.1 | protocadherin Fat 1-like | 2.03 |
| LOC106580561 | XM_014161750.1 | protein patched homolog 1-like | 2.03 |
| LOC100380784 | XM_014146808.1 | basement membrane-specific heparan sulfate proteoglycan core protein | 2.02 |
| LOC106567317 | XM_014136454.1 | neural proliferation differentiation and control protein 1-like | 2.02 |
| LOC106577760 | XM_014156083.1 | PERQ amino acid-rich with GYF domain-containing protein 1-like | 2.02 |
| LOC106568900 | XM_014139690.1 | lipid phosphate phosphatase-related protein type 4-like | 2.02 |
| LOC106579300 | XM_014159100.1 | uncharacterized LOC106579300 | 2.01 |
| LOC106606600 | XM_014202906.1 | trinucleotide repeat-containing gene 6C protein-like | 2.01 |
| LOC106579925 | XM_014160329.1 | cationic amino acid transporter 4-like | 2.01 |
| vps13a | XM_014172179.1 | vacuolar protein sorting 13 homolog A (S. cerevisiae) | 2.01 |
| LOC106582171 | XM_014164978.1 | A-kinase anchor protein 11-like | 2.01 |
| LOC106609034 | XM_014207368.1 | wiskott-Aldrich syndrome protein family member 3-like | 2.01 |
| LOC106568132 | XM_014138176.1 | ras-specific guanine nucleotide-releasing factor 2-like | 2.01 |
| LOC106572720 | XM_014147149.1 | uncharacterized LOC106572720 | 2.00 |
| LOC106613214 | XM_014215254.1 | cytoplasmic dynein 2 heavy chain 1-like | 2.00 |
| LOC106589521 | XM_014179606.1 | DNA-directed RNA polymerase III subunit RPC5-like | 2.00 |
| LOC106575544 | XM_014152103.1 | tensin-1-like | 2.00 |
| LOC106584219 | XM_014169258.1 | cytoplasmic polyadenylation element-binding protein 2-like | 2.00 |
| LOC106566329 | XM_014134243.1 | synaptophysin-like | 1.99 |
| LOC106571269 | XM_014144096.1 | collagen alpha-1(XII) chain-like | 1.99 |
| nkcc1a | NM_001123683.1 | Na/K/2Cl co-transporter | 1.99 |
| LOC106584716 | XM_014170233.1 | ubiquitin-conjugating enzyme E2 Q2-like | 1.99 |
| sobp | XM_014192170.1 | sine oculis binding protein homolog | 1.99 |
| cux2 | XM_014160397.1 | cut-like homeobox 2 | 1.99 |
| LOC106570633 | XM_014143099.1 | F-box/LRR-repeat protein 2-like | 1.98 |
| LOC106575743 | XM_014152416.1 | protein unc-80 homolog | 1.98 |
| LOC106585713 | XM_014172239.1 | cytoplasmic phosphatidylinositol transfer protein 1-like | 1.98 |
| LOC106607176 | XM_014203842.1 | histone deacetylase 5-like | 1.97 |
| LOC106583168 | XM_014167035.1 | electroneutral sodium bicarbonate exchanger 1-like | 1.97 |
| LOC106590617 | XM_014181747.1 | beta-1%2C4-mannosyl-glycoprotein 4-beta-N-acetylglucosaminyltransferase-like | 1.97 |
| LOC106565730 | XM_014133161.1 | contactin-3-like | 1.97 |
| syt17 | XM_014194413.1 | synaptotagmin XVII | 1.97 |
| gfod1 | XM_014181121.1 | glucose-fructose oxidoreductase domain containing 1 | 1.97 |
| LOC106565081 | XM_014131720.1 | calmodulin-binding transcription activator 2-like | 1.97 |
| LOC106611429 | XM_014211625.1 | glioma tumor suppressor candidate region gene 1 protein-like | 1.97 |
| LOC106594098 | XM_014185469.1 | shaker-related potassium channel tsha2-like | 1.96 |
| LOC106562829 | XM_014127851.1 | ral guanine nucleotide dissociation stimulator-like | 1.96 |
| LOC106601859 | XM_014194274.1 | uncharacterized serine-rich protein C215.13-like | 1.96 |
| LOC100380696 | XM_014152358.1 | fibronectin | 1.96 |
| LOC106605949 | XM_014201961.1 | tyrosine-protein phosphatase non-receptor type 3-like | 1.96 |
| LOC106591034 | XM_014182204.1 | glycogenin-1-like | 1.96 |
| LOC106582279 | XM_014165186.1 | leishmanolysin-like peptidase | 1.96 |
| ptch1 | XM_014171042.1 | patched 1 | 1.96 |
| LOC106570525 | XM_014142941.1 | juxtaposed with another zinc finger protein 1 | 1.96 |
| LOC106609675 | XM_014208646.1 | exocyst complex component 4-like | 1.95 |
| LOC106582221 | XM_014165078.1 | A-kinase anchor protein 11-like | 1.95 |
| btbd7 | XM_014145062.1 | BTB (POZ) domain containing 7 | 1.95 |
| LOC106563134 | XM_014128379.1 | voltage-dependent N-type calcium channel subunit alpha-1B-like | 1.95 |
| LOC106573195 | XM_014147980.1 | solute carrier family 45 member 3-like | 1.95 |
| LOC106576414 | XM_014153573.1 | POU domain%2C class 2%2C transcription factor 2-like | 1.94 |
| LOC106603934 | XM_014198188.1 | calcium/calmodulin-dependent protein kinase kinase 1-like | 1.94 |
| LOC106611949 | XM_014212651.1 | A disintegrin and metalloproteinase with thrombospondin motifs 2-like | 1.94 |
| LOC106573269 | XM_014148178.1 | UPF0606 protein KIAA1549-like | 1.94 |
| LOC106599334 | XM_014190509.1 | sickle tail protein homolog | 1.94 |
| LOC106566796 | XM_014135196.1 | protein SOGA1-like | 1.94 |
| LOC106571799 | XM_014145259.1 | forkhead box protein O3-like | 1.94 |
| LOC106610770 | XM_014210326.1 | filamin-A-interacting protein 1-like | 1.94 |
| dock5 | XM_014160188.1 | dedicator of cytokinesis 5 | 1.94 |
| LOC106569531 | XM_014140980.1 | transportin-1-like | 1.93 |
| LOC106612174 | XM_014213111.1 | rho guanine nucleotide exchange factor 9 | 1.93 |
| acacb | XM_014160782.1 | acetyl-CoA carboxylase beta | 1.93 |
| LOC106578063 | XM_014156632.1 | serine/threonine-protein phosphatase 2A 56 kDa regulatory subunit beta isoform-like | 1.93 |
| LOC106605289 | XM_014200762.1 | OTU domain-containing protein 7B-like | 1.93 |
| LOC106577167 | XM_014154979.1 | tau-tubulin kinase 1-like | 1.92 |
| LOC106568602 | XM_014139059.1 | disabled homolog 2-interacting protein-like | 1.92 |
| LOC106574153 | XM_014149798.1 | receptor-type tyrosine-protein phosphatase S-like | 1.92 |
| LOC106611865 | XM_014212478.1 | A disintegrin and metalloproteinase with thrombospondin motifs 2-like | 1.92 |
| LOC106579284 | XM_014159065.1 | uncharacterized LOC106579284 | 1.92 |
| LOC106570613 | XM_014143076.1 | probable serine/threonine-protein kinase kinX | 1.91 |
| LOC106575851 | XM_014152553.1 | Nance-Horan syndrome protein-like | 1.91 |
| LOC106577504 | XM_014155564.1 | ankyrin-2-like | 1.91 |
| LOC106568281 | XM_014138468.1 | phosphatidylinositol 4-kinase alpha-like | 1.91 |
| LOC106560483 | XM_014123444.1 | dedicator of cytokinesis protein 7-like | 1.91 |
| LOC106583531 | XM_014167828.1 | calcium/calmodulin-dependent protein kinase type 1-like | 1.91 |
| LOC106568312 | XM_014138534.1 | membrane-associated phosphatidylinositol transfer protein 2-like | 1.91 |
| LOC106568400 | XM_014138709.1 | integrin alpha-1-like | 1.91 |
| LOC106573992 | XM_014149476.1 | cyclin-dependent kinase-like 5 | 1.90 |
| LOC106578941 | XM_014158229.1 | neurexin-1a-beta-like | 1.90 |
| LOC106585259 | XM_014171270.1 | ankyrin-1-like | 1.90 |
| zfhx3 | XM_014126638.1 | zinc finger homeobox 3 | 1.90 |
| LOC106574979 | XM_014151114.1 | dystrophin-like | 1.90 |
| LOC106591047 | XM_014182234.1 | kinesin-associated protein 3-like | 1.89 |
| LOC106597634 | XM_014188796.1 | tetratricopeptide repeat protein 17-like | 1.89 |
| LOC106573459 | XM_014148523.1 | FERM domain-containing protein 4A-like | 1.89 |
| clock | XM_014169007.1 | clock circadian regulator | 1.89 |
| LOC106570447 | XM_014142790.1 | gamma-aminobutyric acid type B receptor subunit 2-like | 1.89 |
| kmt2d | XM_014135873.1 | lysine (K)-specific methyltransferase 2D | 1.89 |
| LOC106605889 | XM_014201882.1 | RUN and SH3 domain-containing protein 1-like | 1.89 |
| chd3 | XM_014155949.1 | chromodomain helicase DNA binding protein 3 | 1.88 |
| LOC106608439 | XM_014206372.1 | palmitoyltransferase ZDHHC3-like | 1.88 |
| LOC106602308 | XM_014194858.1 | MAX gene-associated protein-like | 1.88 |
| LOC106568226 | XM_014138375.1 | lysine-specific demethylase 2B-like | 1.88 |
| rnf123 | XM_014168343.1 | ring finger protein 123 | 1.88 |
| LOC106578973 | XM_014158322.1 | serine-rich coiled-coil domain-containing protein 2-like | 1.88 |
| LOC106610487 | XM_014209867.1 | protein TANC2-like | 1.88 |
| LOC106592162 | XM_014183471.1 | insulin-like growth factor 1 receptor | 1.88 |
| LOC106576674 | XM_014153915.1 | neuronal cell adhesion molecule-like | 1.88 |
| LOC106572064 | XM_014145841.1 | protein 4.1-like | 1.88 |
| LOC106572368 | XM_014146445.1 | plexin-A1-like | 1.87 |
| LOC106580439 | XM_014161499.1 | histone-lysine N-methyltransferase NSD3-like | 1.87 |
| ralgapa2 | XM_014210399.1 | Ral GTPase activating protein%2C alpha subunit 2 (catalytic) | 1.87 |
| LOC106586920 | XM_014174668.1 | translation initiation factor IF-2-like | 1.87 |
| cd164 | XM_014205492.1 | CD164 molecule%2C sialomucin | 1.87 |
| LOC106563815 | XM_014129707.1 | rapamycin-insensitive companion of mTOR-like | 1.86 |
| LOC106594999 | XM_014186381.1 | protein tweety homolog 3-like | 1.86 |
| LOC106586931 | XM_014174683.1 | neuropilin-2-like | 1.86 |
| LOC106563781 | XM_014129632.1 | protein furry homolog | 1.86 |
| LOC106613701 | XM_014216233.1 | excitatory amino acid transporter 1-like | 1.86 |
| LOC106612815 | XM_014214353.1 | ras-related protein Rab-23-like | 1.86 |
| LOC106585272 | XM_014171314.1 | membrane-associated phosphatidylinositol transfer protein 2-like | 1.85 |
| LOC106569478 | XM_014140866.1 | uncharacterized LOC106569478 | 1.85 |
| fbxl16 | XM_014202492.1 | F-box and leucine-rich repeat protein 16 | 1.85 |
| LOC106612058 | XM_014212875.1 | LON peptidase N-terminal domain and RING finger protein 1-like | 1.85 |
| LOC106562618 | XM_014127575.1 | CREB-regulated transcription coactivator 3-like | 1.85 |
| LOC106578893 | XM_014158131.1 | uncharacterized protein KIAA0195-like | 1.85 |
| cbl | XM_014213633.1 | Cbl proto-oncogene%2C E3 ubiquitin protein ligase | 1.85 |
| LOC106569805 | XM_014141435.1 | probable helicase senataxin | 1.85 |
| LOC106601252 | XM_014193315.1 | histone deacetylase 5-like | 1.84 |
| LOC106570463 | XM_014142825.1 | neurabin-1-like | 1.84 |
| LOC106569018 | XM_014139973.1 | kinesin-like protein KIF26B | 1.84 |
| vdr0 | XM_014146371.1 | vitamin D receptor | 1.84 |
| LOC106609237 | XM_014207789.1 | striatin-interacting protein 1 homolog | 1.84 |
| LOC106611507 | XM_014211774.1 | protein diaphanous homolog 2-like | 1.84 |
| LOC106571009 | XM_014143627.1 | myotubularin-related protein 9-like | 1.83 |
| LOC106574299 | XM_014150103.1 | potassium/sodium hyperpolarization-activated cyclic nucleotide-gated channel 3-like | 1.83 |
| LOC106575892 | XM_014152608.1 | protein FAM117B-like | 1.83 |
| LOC106571361 | XM_014144366.1 | A-kinase anchor protein 7 isoform gamma-like | 1.83 |
| LOC106613636 | XM_014216094.1 | microtubule-associated serine/threonine-protein kinase 3-like | 1.83 |
| LOC106568027 | XM_014138028.1 | homeobox protein cut-like 1 | 1.83 |
| LOC106568376 | XM_014138668.1 | rab GTPase-activating protein 1-like | 1.83 |
| LOC106594565 | XM_014185929.1 | formin-2-like | 1.83 |
| rbm33 | XM_014180672.1 | RNA binding motif protein 33 | 1.83 |
| edc4 | XM_014149081.1 | enhancer of mRNA decapping 4 | 1.83 |
| LOC106573085 | XM_014147770.1 | contactin-1a-like | 1.82 |
| LOC106591933 | XM_014183199.1 | oxysterol-binding protein-related protein 3-like | 1.82 |
| LOC106605998 | XM_014202048.1 | nibrin-like | 1.82 |
| LOC106595407 | XM_014186778.1 | casein kinase I isoform delta-B-like | 1.82 |
| LOC106585658 | XM_014172094.1 | acetyl-CoA carboxylase 2-like | 1.81 |
| LOC106592729 | XM_014184055.1 | GPI transamidase component PIG-S-like | 1.81 |
| rapgef2 | XM_014199228.1 | Rap guanine nucleotide exchange factor (GEF) 2 | 1.81 |
| aplp2 | XM_014215554.1 | amyloid beta (A4) precursor-like protein 2 | 1.81 |
| LOC106606311 | XM_014202467.1 | charged multivesicular body protein 6-like | 1.81 |
| LOC106588669 | XM_014177879.1 | thyroid hormone receptor beta | 1.81 |
| wdr11 | XM_014154429.1 | WD repeat domain 11 | 1.80 |
| dag1 | XM_014168313.1 | dystroglycan 1 (dystrophin-associated glycoprotein 1) | 1.80 |
| LOC106583703 | XM_014168207.1 | glycerol-3-phosphate dehydrogenase [NAD(+)]%2C cytoplasmic-like | 1.80 |
| LOC106590861 | XM_014182039.1 | leucine-rich repeat extensin-like protein 1 | 1.80 |
| LOC106583566 | XM_014167901.1 | CUGBP Elav-like family member 4 | 1.80 |
| LOC106606768 | XM_014203126.1 | voltage-dependent P/Q-type calcium channel subunit alpha-1A-like | 1.80 |
| pcdh9 | XM_014151127.1 | protocadherin 9 | 1.80 |
| ptprr | XM_014208833.1 | protein tyrosine phosphatase%2C receptor type%2C R | 1.79 |
| lrch3 | XM_014213547.1 | leucine-rich repeats and calponin homology (CH) domain containing 3 | 1.79 |
| LOC106612514 | XM_014213716.1 | alpha-2-macroglobulin-like | 1.79 |
| LOC106606599 | XM_014202904.1 | retinoic acid-induced protein 1-like | 1.79 |
| LOC106566323 | XM_014134234.1 | CXXC-type zinc finger protein 1-like | 1.79 |
| LOC106572809 | XM_014147301.1 | tumor necrosis factor receptor superfamily member 5-like | 1.79 |
| nedd4l | XM_014138852.1 | neural precursor cell expressed%2C developmentally down-regulated 4-like%2C E3 ubiquitin protein ligase | 1.79 |
| LOC106585249 | XM_014171260.1 | phosphatidate cytidylyltransferase 2-like | 1.79 |
| LOC106562089 | XM_014126655.1 | copine-7-like | 1.79 |
| ddx6 | XM_014215579.1 | DEAD (Asp-Glu-Ala-Asp) box helicase 6 | 1.79 |
| LOC106597569 | XM_014188737.1 | protein 4.1 homolog | 1.78 |
| LOC106578174 | XM_014156806.1 | tripartite motif-containing protein 2-like | 1.78 |
| LOC106595470 | XM_014186843.1 | serine/threonine-protein kinase PAK 2-like | 1.78 |
| LOC106583746 | XM_014168288.1 | dedicator of cytokinesis protein 3-like | 1.78 |
| LOC106590738 | XM_014181898.1 | beta-1%2C4-mannosyl-glycoprotein 4-beta-N-acetylglucosaminyltransferase-like | 1.78 |
| LOC106586786 | XM_014174419.1 | peripheral plasma membrane protein CASK-like | 1.78 |
| LOC106575987 | XM_014152769.1 | copine-8 | 1.78 |
| LOC106586946 | XM_014174704.1 | ras-associated and pleckstrin homology domains-containing protein 1-like | 1.78 |
| nphp4 | XM_014135428.1 | nephronophthisis 4 | 1.78 |
| LOC106611270 | XM_014211303.1 | endoribonuclease Dicer-like | 1.78 |
| LOC106574143 | XM_014149776.1 | guanine nucleotide-binding protein subunit beta-4 | 1.78 |
| LOC106611370 | XM_014211528.1 | neurexin-3a-like | 1.78 |
| LOC106603820 | XM_014197991.1 | folliculin-interacting protein 1-like | 1.78 |
| LOC106560530 | XM_014123504.1 | microtubule-associated serine/threonine-protein kinase 2-like | 1.77 |
| LOC106602516 | XM_014195192.1 | ubiquitin-like modifier-activating enzyme 6 | 1.77 |
| LOC106588723 | XM_014177998.1 | uncharacterized LOC106588723 | 1.77 |
| LOC106589430 | XM_014179414.1 | neurotrypsin-like | 1.77 |
| LOC106582455 | XM_014165579.1 | dedicator of cytokinesis protein 9-like | 1.77 |
| LOC106610776 | XM_014210337.1 | disks large-associated protein 2-like | 1.77 |
| LOC106564132 | XM_014130147.1 | lysosomal alpha-glucosidase-like | 1.77 |
| LOC106604864 | XM_014199940.1 | janus kinase and microtubule-interacting protein 1-like | 1.77 |
| LOC106583767 | XM_014168326.1 | homeodomain-interacting protein kinase 1-like | 1.77 |
| slc7a3 | XM_014199635.1 | solute carrier family 7 (cationic amino acid transporter%2C y+ system)%2C member 3 | 1.77 |
| LOC106599465 | XM_014190712.1 | nuclear receptor coactivator 2-like | 1.76 |
| LOC106586426 | XM_014173671.1 | CLIP-associating protein 1-B-like | 1.76 |
| LOC106591770 | XM_014182978.1 | trinucleotide repeat-containing gene 18 protein-like | 1.76 |
| LOC106613097 | XM_014215015.1 | unconventional myosin-XVIIIa-like | 1.76 |
| LOC106610979 | XM_014210738.1 | leucine-rich repeat-containing protein 9-like | 1.76 |
| LOC106573256 | XM_014148161.1 | retinoblastoma-like protein 2 | 1.76 |
| LOC106566728 | XM_014135048.1 | nuclear receptor coactivator 3-like | 1.76 |
| sfmbt2 | XM_014207878.1 | Scm-like with four mbt domains 2 | 1.76 |
| LOC106583686 | XM_014168179.1 | proto-oncogene tyrosine-protein kinase Src-like | 1.75 |
| i2c3 | XM_014176828.1 | Eukaryotic translation initiation factor 2C 3 | 1.75 |
| LOC106574911 | XM_014151046.1 | multiple epidermal growth factor-like domains protein 8 | 1.75 |
| LOC106561324 | XM_014125162.1 | serine/threonine-protein kinase WNK1-like | 1.75 |
| otof | XM_014145198.1 | otoferlin | 1.75 |
| micall1 | XM_014130683.1 | MICAL-like 1 | 1.75 |
| LOC106572997 | XM_014147591.1 | potassium voltage-gated channel subfamily A member 2-like | 1.75 |
| LOC106593268 | XM_014184606.1 | protein flightless-1 homolog | 1.75 |
| LOC106584443 | XM_014169785.1 | CUGBP Elav-like family member 5 | 1.74 |
| LOC106577394 | XM_014155365.1 | acyl-CoA desaturase-like | 1.74 |
| LOC106588748 | XM_014178086.1 | upstream-binding protein 1-like | 1.74 |
| LOC106568585 | XM_014139033.1 | uncharacterized LOC106568585 | 1.74 |
| LOC106561729 | XM_014125948.1 | tumor protein p53-inducible protein 11-like | 1.73 |
| LOC106563093 | XM_014128322.1 | uncharacterized LOC106563093 | 1.73 |
| LOC106584763 | XM_014170311.1 | potassium voltage-gated channel subfamily C member 1-like | 1.73 |
| LOC106565077 | XM_014131710.1 | solute carrier family 12 member 5-like | 1.73 |
| LOC106588140 | XM_014176832.1 | aquaporin-3-like | 1.73 |
| LOC106562412 | XM_014127283.1 | membrane-associated phosphatidylinositol transfer protein 2-like | 1.73 |
| LOC106566892 | XM_014135492.1 | disintegrin and metalloproteinase domain-containing protein 17-like | 1.73 |
| LOC106607841 | XM_014205259.1 | protein ZNF365-like | 1.73 |
| LOC106603484 | XM_014197244.1 | proline-rich protein 7-like | 1.73 |
| LOC106577281 | XM_014155222.1 | uncharacterized LOC106577281 | 1.73 |
| ahr2b | NM_001123556.1 | aryl hydrocarbon receptor 2 beta | 1.73 |
| LOC106589035 | XM_014178683.1 | eyes absent homolog 3-like | 1.73 |
| LOC106575885 | XM_014152596.1 | gastrula zinc finger protein XlCGF17.1-like | 1.73 |
| LOC106588641 | XM_014177818.1 | transcription factor HIVEP3-like | 1.72 |
| LOC106572628 | XM_014146986.1 | serine/threonine-protein phosphatase 5-like | 1.72 |
| LOC106611291 | XM_014211337.1 | pleckstrin homology domain-containing family H member 1-like | 1.72 |
| LOC106560750 | XM_014123959.1 | zinc finger protein 609-like | 1.72 |
| cnnm3 | XM_014128009.1 | cyclin and CBS domain divalent metal cation transport mediator 3 | 1.72 |
| LOC106589275 | XM_014179026.1 | helicase SRCAP-like | 1.72 |
| LOC106589506 | XM_014179577.1 | serine/threonine-protein kinase SBK1-like | 1.72 |
| LOC106574855 | XM_014150973.1 | R3H domain-containing protein 1-like | 1.72 |
| LOC106588045 | XM_014176710.1 | low-density lipoprotein receptor-related protein 3-like | 1.71 |
| LOC106565713 | XM_014133120.1 | SLIT-ROBO Rho GTPase-activating protein 2-like | 1.71 |
| LOC106568675 | XM_014139196.1 | serine/threonine-protein kinase PRP4 homolog | 1.71 |
| LOC106613037 | XM_014214916.1 | serine/threonine-protein kinase TAO1-like | 1.71 |
| LOC106566447 | XM_014134494.1 | syntaphilin-like | 1.71 |
| hdac4 | XM_014164292.1 | histone deacetylase 4 | 1.71 |
| stxbp5 | XM_014145279.1 | syntaxin binding protein 5 (tomosyn) | 1.71 |
| zcchc8 | XM_014161314.1 | zinc finger%2C CCHC domain containing 8 | 1.71 |
| LOC106607600 | XM_014204689.1 | adenosine 3'-phospho 5'-phosphosulfate transporter 1-like | 1.71 |
| nck2 | XM_014174379.1 | NCK adaptor protein 2 | 1.71 |
| arfgef2 | XM_014145961.1 | ADP-ribosylation factor guanine nucleotide-exchange factor 2 (brefeldin A-inhibited) | 1.71 |
| LOC106583942 | XM_014168640.1 | tumor suppressor candidate 5 homolog | 1.70 |
| LOC106565299 | XM_014132244.1 | voltage-dependent L-type calcium channel subunit alpha-1D-like | 1.70 |
| LOC106562309 | XM_014127101.1 | protein lin-54 homolog | 1.70 |
| LOC106579370 | XM_014159210.1 | protein phosphatase 1 regulatory subunit 1B-like | 1.70 |
| LOC106606041 | XM_014202115.1 | kinesin-like protein KIF13A | 1.70 |
| LOC106603105 | XM_014196330.1 | leucine-rich repeat and calponin homology domain-containing protein 2-like | 1.70 |
| LOC106588883 | XM_014178393.1 | inactive phospholipase C-like protein 2 | 1.70 |
| LOC106602439 | XM_014195080.1 | protein WWC2-like | 1.69 |
| LOC106567020 | XM_014135795.1 | serine/threonine-protein kinase WNK2-like | 1.69 |
| LOC106567401 | XM_014136584.1 | peripheral-type benzodiazepine receptor-associated protein 1-like | 1.69 |
| LOC106601438 | XM_014193653.1 | RNA binding protein fox-1 homolog 3-like | 1.69 |
| LOC106601595 | XM_014193918.1 | dynamin-2-like | 1.69 |
| LOC106608994 | XM_014207312.1 | phosphofurin acidic cluster sorting protein 1-like | 1.69 |
| LOC106573061 | XM_014147715.1 | CUGBP Elav-like family member 2 | 1.69 |
| LOC106580493 | XM_014161626.1 | methylcytosine dioxygenase TET3-like | 1.69 |
| LOC106604912 | XM_014200037.1 | ras-GEF domain-containing family member 1C-like | 1.69 |
| pusl1 | XM_014168350.1 | pseudouridylate synthase-like 1 | 1.69 |
| LOC106602984 | XM_014196077.1 | pecanex-like protein 1 | 1.69 |
| LOC106567492 | XM_014136782.1 | glucocorticoid receptor-like | 1.69 |
| LOC106604036 | XM_014198371.1 | B-cell receptor CD22-like | 1.69 |
| LOC106610280 | XM_014209542.1 | general vesicular transport factor p115-like | 1.68 |
| fbxo15 | XM_014157193.1 | F-box protein 15 | 1.68 |
| vps39 | XM_014211709.1 | vacuolar protein sorting 39 homolog (S. cerevisiae) | 1.68 |
| LOC106610942 | XM_014210634.1 | uncharacterized protein KIAA1109-like | 1.68 |
| LOC106570883 | XM_014143453.1 | DENN domain-containing protein 1A-like | 1.68 |
| LOC106606288 | XM_014202432.1 | zinc finger protein 239-like | 1.68 |
| LOC106577545 | XM_014155660.1 | equilibrative nucleoside transporter 2-like | 1.68 |
| LOC106580373 | XM_014161353.1 | probable E3 ubiquitin-protein ligase HECTD4 | 1.68 |
| LOC106560529 | XM_014123503.1 | phosphatidylinositol 3-kinase regulatory subunit gamma-like | 1.68 |
| LOC106590429 | XM_014181455.1 | histone-lysine N-methyltransferase 2C-like | 1.67 |
| LOC106587746 | XM_014176363.1 | inositol hexakisphosphate and diphosphoinositol-pentakisphosphate kinase 2-like | 1.67 |
| LOC106587514 | XM_014175965.1 | synaptotagmin-7-like | 1.67 |
| pkdcc | XM_014201656.1 | protein kinase domain containing%2C cytoplasmic | 1.67 |
| papolg | XM_014216469.1 | poly(A) polymerase gamma | 1.67 |
| LOC106560721 | XM_014123886.1 | ankyrin repeat domain-containing protein 26-like | 1.67 |
| LOC106584876 | XM_014170481.1 | uncharacterized LOC106584876 | 1.67 |
| LOC106587736 | XM_014176343.1 | mitogen-activated protein kinase 6-like | 1.67 |
| LOC106612385 | XM_014213478.1 | suppressor of cytokine signaling 3-like | 1.67 |
| LOC106581932 | XM_014164439.1 | calcitonin gene-related peptide type 1 receptor | 1.67 |
| kmt2a | XM_014213640.1 | lysine (K)-specific methyltransferase 2A | 1.66 |
| LOC106579141 | XM_014158736.1 | zinc finger protein 91-like | 1.66 |
| LOC106575383 | XM_014151888.1 | serine/threonine-protein phosphatase 2A regulatory subunit B'' subunit alpha-like | 1.66 |
| LOC106565177 | XM_014132020.1 | voltage-dependent calcium channel subunit alpha-2/delta-2-like | 1.66 |
| LOC106593034 | XM_014184379.1 | STIP1 homology and U box-containing protein 1-like | 1.66 |
| LOC106575774 | XM_014152454.1 | glycerol-3-phosphate dehydrogenase%2C mitochondrial-like | 1.66 |
| LOC106561902 | XM_014126233.1 | protein kinase C-binding protein NELL1-like | 1.66 |
| ip6k1 | XM_014168331.1 | inositol hexakisphosphate kinase 1 | 1.66 |
| LOC106560308 | XM_014123072.1 | zinc finger protein 644-like | 1.66 |
| LOC106603377 | XM_014196969.1 | long-chain-fatty-acid--CoA ligase 4-like | 1.66 |
| LOC106570630 | XM_014143095.1 | CLIP-associating protein 2-like | 1.66 |
| strn4 | XM_014213497.1 | striatin%2C calmodulin binding protein 4 | 1.66 |
| LOC106594520 | XM_014185897.1 | protein kinase C beta type | 1.66 |
| LOC106567057 | XM_014135894.1 | low-density lipoprotein receptor-related protein 1-like | 1.66 |
| LOC106608678 | XM_014206777.1 | zinc finger and BTB domain-containing protein 38-like | 1.65 |
| LOC106565696 | XM_014133483.1 | rab11 family-interacting protein 5-like | 1.65 |
| LOC106581879 | XM_014164319.1 | type I inositol 3%2C4-bisphosphate 4-phosphatase-like | 1.65 |
| LOC106572786 | XM_014147257.1 | disco-interacting protein 2 homolog B-A-like | 1.65 |
| LOC106588578 | XM_014177721.1 | LIX1-like protein | 1.65 |
| LOC106585120 | XM_014170969.1 | substance-P receptor-like | 1.65 |
| LOC106560488 | XM_014123453.1 | dedicator of cytokinesis protein 7-like | 1.65 |
| LOC106602556 | XM_014195248.1 | protocadherin Fat 1-like | 1.65 |
| LOC106564141 | XM_014130157.1 | protein tweety homolog 3-like | 1.65 |
| LOC106595830 | XM_014187176.1 | LIM and calponin homology domains-containing protein 1-like | 1.65 |
| arhgef1 | XM_014201601.1 | Rho guanine nucleotide exchange factor (GEF) 1 | 1.65 |
| LOC106608576 | XM_014206565.1 | ankyrin-2-like | 1.65 |
| LOC106609844 | XM_014208866.1 | exportin-T-like | 1.65 |
| LOC106573044 | XM_014147667.1 | membrane-associated guanylate kinase%2C WW and PDZ domain-containing protein 2-like | 1.64 |
| LOC106603506 | XM_014197286.1 | nesprin-1-like | 1.64 |
| LOC106611061 | XM_014210912.1 | latent-transforming growth factor beta-binding protein 2-like | 1.64 |
| LOC106585296 | XM_014171366.1 | probable E3 ubiquitin-protein ligase HECTD4 | 1.64 |
| ablim2 | XM_014206542.1 | actin binding LIM protein family%2C member 2 | 1.64 |
| LOC106584848 | XM_014170451.1 | mucin-2-like | 1.64 |
| LOC106563331 | XM_014128850.1 | poly [ADP-ribose] polymerase 14-like | 1.64 |
| LOC106607272 | XM_014204072.1 | F-box/LRR-repeat protein 20 | 1.64 |
| agt | NM_001140446.1 | angiotensinogen (serpin peptidase inhibitor%2C clade A%2C member 8) | 1.64 |
| xk | XM_014174210.1 | X-linked Kx blood group | 1.64 |
| LOC106561234 | XM_014124953.1 | C-Jun-amino-terminal kinase-interacting protein 2-like | 1.64 |
| LOC106586262 | XM_014173338.1 | dedicator of cytokinesis protein 9-like | 1.64 |
| LOC106580084 | XM_014160710.1 | vacuolar protein sorting-associated protein 13A-like | 1.64 |
| LOC106570436 | XM_014142770.1 | ral guanine nucleotide dissociation stimulator-like | 1.64 |
| LOC106584061 | XM_014168886.1 | terminal uridylyltransferase 4-like | 1.64 |
| LOC106568090 | XM_014138113.1 | amyloid protein-binding protein 2-like | 1.63 |
| ubr4 | XM_014147487.1 | ubiquitin protein ligase E3 component n-recognin 4 | 1.63 |
| LOC106564512 | XM_014130636.1 | transcription activator BRG1-like | 1.63 |
| LOC106580569 | XM_014161766.1 | RING finger protein 165-like | 1.63 |
| sdccag8 | XM_014179967.1 | serologically defined colon cancer antigen 8 | 1.63 |
| map3k12 | XM_014134036.1 | mitogen-activated protein kinase kinase kinase 12 | 1.63 |
| trrap | XM_014179710.1 | transformation/transcription domain-associated protein | 1.63 |
| exd2 | XM_014155462.1 | exonuclease 3'-5' domain containing 2 | 1.63 |
| LOC106572005 | XM_014145694.1 | E3 ubiquitin-protein ligase MIB2-like | 1.63 |
| LOC106605615 | XM_014201449.1 | histone-lysine N-methyltransferase 2A-like | 1.63 |
| LOC106582497 | XM_014165662.1 | integrator complex subunit 6-like | 1.63 |
| LOC106599430 | XM_014190665.1 | laminin subunit gamma-1-like | 1.63 |
| LOC106569192 | XM_014140291.1 | histone-lysine N-methyltransferase 2C-like | 1.63 |
| LOC106563328 | XM_014128843.1 | ankyrin-1-like | 1.63 |
| LOC106603889 | XM_014198100.1 | ubiquitin-like domain-containing CTD phosphatase 1 | 1.63 |
| LOC106603896 | XM_014198112.1 | grpE protein homolog 2%2C mitochondrial-like | 1.63 |
| LOC106583004 | XM_014166712.1 | leucine-rich repeats and immunoglobulin-like domains protein 2 | 1.63 |
| LOC106567782 | XM_014137511.1 | stAR-related lipid transfer protein 13-like | 1.63 |
| LOC106577316 | XM_014155272.1 | proto-oncogene c-Rel-like | 1.63 |
| LOC106565160 | XM_014131966.1 | serine/threonine-protein kinase WNK2-like | 1.62 |
| ppc1a | NM_001146657.1 | Phosphatidic acid phosphatase type 2 domain-containing protein 1A | 1.62 |
| LOC106587928 | XM_014176587.1 | C-myc promoter-binding protein-like | 1.62 |
| ptchd1 | XM_014181864.1 | patched domain containing 1 | 1.62 |
| LOC106576932 | XM_014154471.1 | heat shock 70 kDa protein 12A-like | 1.62 |
| LOC106565828 | XM_014133395.1 | FYVE%2C RhoGEF and PH domain-containing protein 5-like | 1.62 |
| yeats2 | XM_014192065.1 | YEATS domain containing 2 | 1.62 |
| LOC106577338 | XM_014155305.1 | transmembrane protein 145-like | 1.62 |
| LOC106609821 | XM_014208846.1 | putative homeodomain transcription factor 2 | 1.62 |
| LOC106562605 | XM_014127555.1 | glutamine and serine-rich protein 1-like | 1.62 |
| LOC106572764 | XM_014147233.1 | ceramide glucosyltransferase-B | 1.62 |
| LOC106576490 | XM_014153694.1 | receptor-type tyrosine-protein phosphatase beta-like | 1.62 |
| LOC106588378 | XM_014177293.1 | major histocompatibility complex class I-related gene protein-like | 1.62 |
| LOC106572904 | XM_014147488.1 | arginine-glutamic acid dipeptide repeats protein-like | 1.62 |
| LOC106566301 | XM_014134205.1 | arginine-glutamic acid dipeptide repeats protein-like | 1.62 |
| LOC106582631 | XM_014165861.1 | receptor-type tyrosine-protein phosphatase T-like | 1.62 |
| LOC106603776 | XM_014197852.1 | ribosomal protein S6 kinase alpha-5-like | 1.61 |
| LOC106579490 | XM_014159434.1 | disco-interacting protein 2 homolog C-like | 1.61 |
| LOC106579463 | XM_014159394.1 | acyl-coenzyme A thioesterase 9%2C mitochondrial-like | 1.61 |
| LOC106611247 | XM_014211251.1 | uncharacterized LOC106611247 | 1.61 |
| LOC100136526 | XM_014131866.1 | peroxisome proliferator-activated receptor delta | 1.61 |
| LOC106598586 | XM_014189618.1 | serine/arginine repetitive matrix protein 2-like | 1.61 |
| LOC106577155 | XM_014154958.1 | mitogen-activated protein kinase kinase kinase kinase 3-like | 1.61 |
| LOC106578615 | XM_014157618.1 | receptor-type tyrosine-protein phosphatase N2-like | 1.61 |
| LOC106578422 | XM_014157181.1 | catenin delta-2-like | 1.61 |
| LOC106596416 | XM_014187709.1 | ZZ-type zinc finger-containing protein 3-like | 1.61 |
| LOC106613592 | XM_014216012.1 | dual specificity protein phosphatase CDC14A-like | 1.61 |
| LOC106605718 | XM_014201621.1 | chromodomain-helicase-DNA-binding protein 4-like | 1.61 |
| LOC106610732 | XM_014210248.1 | consortin-like | 1.61 |
| LOC106574311 | XM_014150144.1 | pyridoxal kinase-like | 1.61 |
| LOC106567646 | XM_014137180.1 | opioid-binding protein/cell adhesion molecule-like | 1.61 |
| LOC106607167 | XM_014203808.1 | histone acetyltransferase p300-like | 1.61 |
| LOC106575549 | XM_014152113.1 | uncharacterized LOC106575549 | 1.61 |
| LOC106563682 | XM_014129474.1 | H(+)/Cl(-) exchange transporter 5-like | 1.61 |
| ptchd2 | XM_014160136.1 | patched domain containing 2 | 1.61 |
| LOC106588934 | XM_014178493.1 | pyruvate dehydrogenase (acetyl-transferring) kinase isozyme 2%2C mitochondrial-like | 1.61 |
| LOC106581263 | XM_014163213.1 | A-kinase anchor protein 1%2C mitochondrial-like | 1.60 |
| LOC106572961 | XM_014147560.1 | ubiquitin-like modifier-activating enzyme 1 | 1.60 |
| LOC106581600 | XM_014163723.1 | glioma tumor suppressor candidate region gene 1 protein-like | 1.60 |
| aatk | XM_014212696.1 | apoptosis-associated tyrosine kinase | 1.60 |
| LOC106592637 | XM_014183976.1 | RNA-binding protein 10-like | 1.60 |
| heatr5a | XM_014194302.1 | HEAT repeat containing 5A | 1.60 |
| LOC106590469 | XM_014181530.1 | basic helix-loop-helix domain-containing protein KIAA2018-like | 1.60 |
| LOC106577519 | XM_014155615.1 | ras-related protein Rab-1B | 1.60 |
| kiaa0226 | XM_014149850.1 | KIAA0226 ortholog | 1.60 |
| LOC106579917 | XM_014160293.1 | E1A-binding protein p400-like | 1.60 |
| LOC106584104 | XM_014168941.1 | protein furry homolog-like | 1.60 |
| LOC106588695 | XM_014177955.1 | non-canonical poly(A) RNA polymerase PAPD7-like | 1.60 |
| LOC106567672 | XM_014137229.1 | cell surface glycoprotein MUC18-like | 1.59 |
| LOC106561041 | XM_014124611.1 | cadherin EGF LAG seven-pass G-type receptor 1-like | 1.59 |
| nbeal1 | XM_014149439.1 | neurobeachin-like 1 | 1.59 |
| LOC106567530 | XM_014136888.1 | protein Jade-1-like | 1.59 |
| gpt2 | XM_014126510.1 | glutamic pyruvate transaminase (alanine aminotransferase) 2 | 1.59 |
| LOC106602627 | XM_014195383.1 | spectrin beta chain%2C erythrocytic-like | 1.59 |
| LOC106613240 | XM_014215308.1 | rho guanine nucleotide exchange factor 17-like | 1.59 |
| LOC106564302 | XM_014130339.1 | transportin-2-like | 1.59 |
| LOC106589111 | XM_014178807.1 | gap junction alpha-4 protein-like | 1.59 |
| LOC106579616 | XM_014159705.1 | zinc finger homeobox protein 4-like | 1.59 |
| LOC106603108 | XM_014196337.1 | serine/threonine-protein kinase PAK 3-like | 1.59 |
| LOC106592297 | XM_014183625.1 | G protein-coupled receptor kinase 6-like | 1.59 |
| LOC106606681 | XM_014203021.1 | serine/threonine-protein kinase/endoribonuclease IRE1-like | 1.59 |
| LOC106565428 | XM_014132551.1 | natural resistance-associated macrophage protein 2-like | 1.59 |
| LOC106588060 | XM_014176726.1 | Golgi apparatus protein 1-like | 1.59 |
| LOC106571902 | XM_014145442.1 | IQ motif and SEC7 domain-containing protein 1-like | 1.59 |
| LOC106565061 | XM_014131688.1 | dystroglycan-like | 1.58 |
| LOC106565528 | XM_014132764.1 | beta-1%2C4 N-acetylgalactosaminyltransferase 1-like | 1.58 |
| LOC106565476 | XM_014132695.1 | inositol 1%2C4%2C5-trisphosphate receptor type 1 | 1.58 |
| LOC106577926 | XM_014156395.1 | histone-lysine N-methyltransferase 2B-like | 1.58 |
| LOC106563220 | XM_014128579.1 | dihydropyrimidinase-related protein 2 | 1.58 |
| LOC106571417 | XM_014144494.1 | transcription factor HIVEP2-like | 1.58 |
| LOC106588247 | XM_014177065.1 | histone-lysine N-methyltransferase 2B-like | 1.58 |
| LOC106579406 | XM_014159276.1 | serine/threonine-protein kinase TAO2-like | 1.57 |
| LOC106566442 | XM_014134482.1 | solute carrier organic anion transporter family member 4A1-like | 1.57 |
| LOC106612163 | XM_014213099.1 | cohesin subunit SA-2-like | 1.57 |
| LOC106565096 | XM_014131802.1 | phosphatidylinositol 4-phosphate 3-kinase C2 domain-containing subunit beta-like | 1.57 |
| LOC106569851 | XM_014141529.1 | A-kinase anchor protein 9-like | 1.57 |
| LOC106593155 | XM_014184491.1 | serine/arginine repetitive matrix protein 2-like | 1.57 |
| LOC106562453 | XM_014127345.1 | selenocysteine insertion sequence-binding protein 2-like | 1.57 |
| LOC106572354 | XM_014146414.1 | 6-phosphofructo-2-kinase/fructose-2%2C6-bisphosphatase 2-like | 1.57 |
| LOC106584282 | XM_014169349.1 | protein zyg-11 homolog | 1.57 |
| LOC106597731 | XM_014188879.1 | serine/threonine-protein phosphatase 6 regulatory ankyrin repeat subunit A-like | 1.57 |
| LOC106600879 | XM_014192624.1 | synaptogyrin-1-like | 1.57 |
| ppp1r9a | XM_014178495.1 | protein phosphatase 1%2C regulatory subunit 9A | 1.57 |
| LOC106570093 | XM_014142078.1 | histone-lysine N-methyltransferase ASH1L-like | 1.57 |
| LOC106579633 | XM_014159734.1 | nectin-1-like | 1.57 |
| LOC106587237 | XM_014175434.1 | immunoglobulin superfamily DCC subclass member 4-like | 1.57 |
| LOC106583764 | XM_014168323.1 | arf-GAP with coiled-coil%2C ANK repeat and PH domain-containing protein 3-like | 1.57 |
| LOC106577598 | XM_014155794.1 | excitatory amino acid transporter 3-like | 1.56 |
| LOC106609667 | XM_014208636.1 | alpha-N-acetylneuraminide alpha-2%2C8-sialyltransferase-like | 1.56 |
| LOC106574832 | XM_014150927.1 | R3H domain-containing protein 1-like | 1.56 |
| LOC106606314 | XM_014202471.1 | hepatocyte growth factor-regulated tyrosine kinase substrate-like | 1.56 |
| LOC106575618 | XM_014152230.1 | histone deacetylase 4-like | 1.56 |
| LOC106590401 | XM_014181380.1 | brefeldin A-inhibited guanine nucleotide-exchange protein 1-like | 1.56 |
| igsf8 | XM_014133993.1 | immunoglobulin superfamily%2C member 8 | 1.56 |
| vps13d | XM_014135237.1 | vacuolar protein sorting 13 homolog D (S. cerevisiae) | 1.56 |
| LOC106565466 | XM_014132678.1 | low-density lipoprotein receptor-related protein 1-like | 1.56 |
| LOC106569707 | XM_014141277.1 | zinc finger protein 236-like | 1.56 |
| syne1 | XM_014205626.1 | spectrin repeat containing%2C nuclear envelope 1 | 1.56 |
| LOC106603258 | XM_014196678.1 | human immunodeficiency virus type I enhancer-binding protein 2 homolog | 1.55 |
| sos2 | XM_014193968.1 | son of sevenless homolog 2 (Drosophila) | 1.55 |
| LOC106584534 | XM_014169938.1 | Golgi apparatus protein 1-like | 1.55 |
| LOC106600538 | XM_014191960.1 | zinc finger protein 512B-like | 1.55 |
| vamp7 | XM_014198467.1 | vesicle-associated membrane protein 7 | 1.55 |
| mtmr4 | XM_014136579.1 | myotubularin related protein 4 | 1.55 |
| vps13c | XM_014176678.1 | vacuolar protein sorting 13 homolog C (S. cerevisiae) | 1.55 |
| podxl | XM_014208089.1 | podocalyxin-like | 1.55 |
| LOC106585950 | XM_014172703.1 | chondroitin sulfate proteoglycan 4-like | 1.55 |
| LOC106594799 | XM_014186186.1 | zinc finger protein 345-like | 1.55 |
| LOC106586162 | XM_014173077.1 | formin-like protein 2 | 1.55 |
| LOC106561621 | XM_014125750.1 | spermatid perinuclear RNA-binding protein-like | 1.55 |
| LOC106583663 | XM_014168118.1 | E3 ubiquitin-protein ligase Itchy-like | 1.55 |
| LOC106568365 | XM_014138650.1 | mucin-5AC-like | 1.55 |
| LOC106594751 | XM_014186169.1 | 60 kDa lysophospholipase-like | 1.55 |
| LOC106566913 | XM_014135516.1 | UHRF1-binding protein 1-like | 1.54 |
| znf827 | XM_014156831.1 | zinc finger protein 827 | 1.54 |
| kifap3 | XM_014123520.1 | kinesin-associated protein 3 | 1.54 |
| LOC106601240 | XM_014193286.1 | protein Tob2-like | 1.54 |
| LOC106587065 | XM_014174962.1 | glycosyltransferase-like protein LARGE2 | 1.54 |
| atp9b | XM_014177039.1 | ATPase%2C class II%2C type 9B | 1.54 |
| fndc3b | XM_014150549.1 | fibronectin type III domain containing 3B | 1.54 |
| LOC106566857 | XM_014135352.1 | msx2-interacting protein-like | 1.54 |
| LOC106575446 | XM_014151946.1 | cohesin subunit SA-1 | 1.53 |
| LOC106563084 | XM_014128307.1 | multiple C2 and transmembrane domain-containing protein 1-like | 1.53 |
| hipk3 | XM_014125488.1 | homeodomain interacting protein kinase 3 | 1.53 |
| LOC106563569 | XM_014129266.1 | CD276 antigen homolog | 1.53 |
| LOC106610139 | XM_014209315.1 | BAH and coiled-coil domain-containing protein 1-like | 1.53 |
| cables1 | XM_014140540.1 | Cdk5 and Abl enzyme substrate 1 | 1.53 |
| tm136 | NM_001140640.1 | Transmembrane protein 136 | 1.53 |
| LOC106612277 | XM_014213294.1 | probable E3 ubiquitin-protein ligase HERC1 | 1.53 |
| LOC106607536 | XM_014204573.1 | pleckstrin homology domain-containing family A member 1-like | 1.53 |
| LOC106571573 | XM_014144796.1 | CLOCK-interacting pacemaker-like | 1.53 |
| LOC106602727 | XM_014195546.1 | uncharacterized LOC106602727 | 1.53 |
| LOC106604536 | XM_014199256.1 | dihydropteridine reductase-like | 1.53 |
| LOC106586971 | XM_014174734.1 | bromodomain-containing protein 3-like | 1.53 |
| LOC106570655 | XM_014143124.1 | alpha-1%2C3-mannosyl-glycoprotein 2-beta-N-acetylglucosaminyltransferase-like | 1.52 |
| LOC106580341 | XM_014161252.1 | ankyrin-1-like | 1.52 |
| LOC106578639 | XM_014157648.1 | sickle tail protein-like | 1.52 |
| dock11 | XM_014206848.1 | dedicator of cytokinesis 11 | 1.52 |
| LOC106611764 | XM_014212315.1 | cationic amino acid transporter 3-like | 1.52 |
| LOC106601392 | XM_014193567.1 | nucleosome-remodeling factor subunit BPTF-like | 1.52 |
| gria4 | XM_014215179.1 | glutamate receptor%2C ionotropic%2C AMPA 4 | 1.52 |
| LOC106566343 | XM_014134267.1 | host cell factor 1-like | 1.52 |
| LOC106562918 | XM_014128019.1 | zinc finger FYVE domain-containing protein 16-like | 1.52 |
| ipp | XM_014140732.1 | intracisternal A particle-promoted polypeptide | 1.52 |
| LOC100380643 | XM_014134249.1 | filamin-A | 1.52 |
| LOC100380701 | XM_014191699.1 | uncharacterized protein KIAA1109 | 1.52 |
| LOC106568813 | XM_014139483.1 | collagen alpha-1(XI) chain-like | 1.52 |
| LOC106591672 | XM_014182896.1 | probable cationic amino acid transporter | 1.51 |
| LOC106561920 | XM_014126314.1 | transcription factor TFIIIB component B'' homolog | 1.51 |
| LOC106572349 | XM_014146396.1 | ankyrin repeat and SAM domain-containing protein 1A-like | 1.51 |
| LOC106589879 | XM_014180291.1 | histone acetyltransferase KAT6B-like | 1.51 |
| LOC106576009 | XM_014152805.1 | multidrug resistance protein 1-like | 1.51 |
| LOC106591328 | XM_014182539.1 | LIM domain-binding protein 1 | 1.51 |
| akt2 | XM_014129610.1 | v-akt murine thymoma viral oncogene homolog 2 | 1.51 |
| LOC106567146 | XM_014136107.1 | dnaJ homolog subfamily C member 16-like | 1.51 |
| gad2 | XM_014181019.1 | glutamate decarboxylase 2 (pancreatic islets and brain%2C 65kDa) | 1.51 |
| LOC106574701 | XM_014150712.1 | formin-like protein 2 | 1.51 |
| LOC106576369 | XM_014153496.1 | UHRF1-binding protein 1-like | 1.51 |
| dmxl1 | XM_014172539.1 | Dmx-like 1 | 1.50 |
| hecw2 | XM_014172970.1 | HECT%2C C2 and WW domain containing E3 ubiquitin protein ligase 2 | 1.50 |
| katnal1 | XM_014129722.1 | katanin p60 subunit A-like 1 | 1.50 |
| LOC106569240 | XM_014140414.1 | calcium/calmodulin-dependent protein kinase type II subunit beta-like | 1.50 |
| pxk | XM_014168403.1 | PX domain containing serine/threonine kinase | 1.50 |
| LOC106613957 | XM_014216815.1 | ras-specific guanine nucleotide-releasing factor RalGPS2 | 1.50 |
| LOC106588368 | XM_014177277.1 | zinc fingers and homeoboxes protein 2-like | 1.50 |
| LOC106593684 | XM_014185056.1 | uncharacterized LOC106593684 | 1.50 |
| LOC106565749 | XM_014133211.1 | inositol 1%2C4%2C5-trisphosphate receptor type 1-like | 1.50 |
| LOC106579810 | XM_014160007.1 | volume-regulated anion channel subunit LRRC8A-like | 1.50 |
| LOC106578854 | XM_014158078.1 | growth arrest-specific protein 7-like | 1.50 |
| LOC106611718 | XM_014212219.1 | sodium/hydrogen exchanger 6-like | 1.50 |
| LOC106572956 | XM_014147548.1 | uncharacterized LOC106572956 | 1.50 |
| parp16 | XM_014170240.1 | poly (ADP-ribose) polymerase family%2C member 16 | 1.50 |
| acaca | XM_014138098.1 | acetyl-CoA carboxylase alpha | 1.49 |
| LOC106584996 | XM_014170713.1 | putative phospholipase B-like 2 | 1.49 |
| ptpn9 | XM_014124056.1 | protein tyrosine phosphatase%2C non-receptor type 9 | 1.49 |
| dennd5a | XM_014124661.1 | DENN/MADD domain containing 5A | 1.49 |
| LOC106569807 | XM_014141433.1 | serine/threonine-protein kinase OSR1-like | 1.49 |
| LOC106564026 | XM_014129998.1 | zinc finger protein 239-like | 1.49 |
| sfxn1 | XM_014213238.1 | sideroflexin 1 | 1.49 |
| lama2 | XM_014205066.1 | laminin%2C alpha 2 | 1.49 |
| LOC106602006 | XM_014194447.1 | ubiquitin carboxyl-terminal hydrolase 7-like | 1.49 |
| LOC106564108 | XM_014130101.1 | bifunctional apoptosis regulator-like | 1.49 |
| LOC106607385 | XM_014204301.1 | KN motif and ankyrin repeat domain-containing protein 2-like | 1.49 |
| LOC106583011 | XM_014166738.1 | ankyrin repeat and SAM domain-containing protein 1A-like | 1.49 |
| LOC106586633 | XM_014174126.1 | MORC family CW-type zinc finger protein 3-like | 1.49 |
| LOC106607262 | XM_014204055.1 | synaptic vesicle membrane protein VAT-1 homolog | 1.49 |
| LOC106583141 | XM_014166998.1 | low-density lipoprotein receptor-related protein 1-like | 1.49 |
| LOC100286503 | XM_014164404.1 | tensin-1 | 1.48 |
| LOC106583613 | XM_014168006.1 | plectin-like | 1.48 |
| rab12 | XM_014141315.1 | RAB12%2C member RAS oncogene family | 1.48 |
| LOC106585250 | XM_014171261.1 | oxysterol-binding protein 2-like | 1.48 |
| LOC106612325 | XM_014213377.1 | transcriptional regulator ATRX-like | 1.48 |
| LOC106574130 | XM_014149749.1 | arf-GAP with coiled-coil%2C ANK repeat and PH domain-containing protein 2-like | 1.48 |
| tmtc3 | XM_014125664.1 | transmembrane and tetratricopeptide repeat containing 3 | 1.48 |
| cfap54 | XM_014195735.1 | cilia and flagella associated 54 | 1.48 |
| LOC106583229 | XM_014167156.1 | signal transducer and activator of transcription 1-like | 1.48 |
| LOC106585077 | XM_014170855.1 | tankyrase-1 | 1.48 |
| LOC106609299 | XM_014207957.1 | filamin-C-like | 1.48 |
| LOC106560551 | XM_014123546.1 | protein crumbs homolog 1-like | 1.48 |
| LOC106601875 | XM_014194301.1 | ubiquitin carboxyl-terminal hydrolase 22-like | 1.48 |
| LOC106563684 | XM_014129482.1 | ras-related protein Rab-39B-like | 1.48 |
| LOC106562084 | XM_014126644.1 | nuclear mitotic apparatus protein 1-like | 1.47 |
| LOC106586635 | XM_014174131.1 | uncharacterized LOC106586635 | 1.47 |
| LOC106608762 | XM_014206884.1 | chromodomain-helicase-DNA-binding protein 3-like | 1.47 |
| LOC106570670 | XM_014143139.1 | phosphatidylinositide phosphatase SAC1-B | 1.47 |
| LOC106570016 | XM_014141877.1 | microtubule-actin cross-linking factor 1-like | 1.47 |
| LOC106563702 | XM_014129516.1 | RNA-binding protein 4-like | 1.47 |
| LOC106612640 | XM_014214040.1 | ribosomal protein S6 kinase alpha-1 | 1.47 |
| LOC106592516 | XM_014183860.1 | uncharacterized LOC106592516 | 1.47 |
| LOC106583546 | XM_014167850.1 | uncharacterized LOC106583546 | 1.47 |
| LOC106576946 | XM_014154494.1 | baculoviral IAP repeat-containing protein 6-like | 1.47 |
| akap11 | XM_014173904.1 | A kinase (PRKA) anchor protein 11 | 1.47 |
| LOC106573267 | XM_014148176.1 | zinc finger CCCH domain-containing protein 13-like | 1.47 |
| kndc1 | XM_014200072.1 | kinase non-catalytic C-lobe domain (KIND) containing 1 | 1.47 |
| LOC106601226 | XM_014193262.1 | transmembrane protein 184B-like | 1.47 |
| LOC106581996 | XM_014164618.1 | type I inositol 3%2C4-bisphosphate 4-phosphatase-like | 1.47 |
| LOC106602602 | XM_014195339.1 | ATP-binding cassette sub-family A member 1-like | 1.46 |
| LOC106603277 | XM_014196713.1 | seizure protein 6-like | 1.46 |
| usp24 | XM_014169242.1 | ubiquitin specific peptidase 24 | 1.46 |
| LOC106583648 | XM_014168079.1 | potassium voltage-gated channel subfamily KQT member 2-like | 1.46 |
| LOC106567418 | XM_014136630.1 | teneurin-2-like | 1.46 |
| LOC106593144 | XM_014184482.1 | zinc finger protein 883-like | 1.46 |
| mllt4 | XM_014205151.1 | myeloid/lymphoid or mixed-lineage leukemia%3B translocated to%2C 4 | 1.46 |
| gtf3c3 | XM_014185889.1 | general transcription factor IIIC%2C polypeptide 3%2C 102kDa | 1.46 |
| LOC106580770 | XM_014162162.1 | uncharacterized LOC106580770 | 1.46 |
| LOC106603436 | XM_014197114.1 | NADPH--cytochrome P450 reductase-like | 1.46 |
| LOC106603839 | XM_014198021.1 | janus kinase and microtubule-interacting protein 2-like | 1.46 |
| LOC106593919 | XM_014185295.1 | WD repeat domain phosphoinositide-interacting protein 2-like | 1.46 |
| LOC106608076 | XM_014205763.1 | MAX gene-associated protein-like | 1.45 |
| LOC106568720 | XM_014139300.1 | tyrosine-protein kinase JAK1-like | 1.45 |
| LOC106584072 | XM_014168912.1 | desmoplakin-like | 1.45 |
| LOC106608363 | XM_014206275.1 | tau-tubulin kinase 1-like | 1.45 |
| hgsnat | XM_014181930.1 | heparan-alpha-glucosaminide N-acetyltransferase | 1.45 |
| LOC106576347 | XM_014153470.1 | glutamate receptor ionotropic%2C kainate 5-like | 1.45 |
| trip11 | XM_014211716.1 | thyroid hormone receptor interactor 11 | 1.45 |
| LOC106580491 | XM_014161618.1 | histone acetyltransferase KAT6A-like | 1.45 |
| LOC106578133 | XM_014156747.1 | leucine-rich repeat and fibronectin type-III domain-containing protein 2-like | 1.45 |
| LOC106569517 | XM_014140950.1 | semaphorin-6B-like | 1.45 |
| LOC106610679 | XM_014210140.1 | nuclear receptor coactivator 7-like | 1.44 |
| LOC106604312 | XM_014198827.1 | ankyrin repeat and KH domain-containing protein 1-like | 1.44 |
| LOC106590181 | XM_014180923.1 | gamma-adducin-like | 1.44 |
| LOC106568339 | XM_014138599.1 | tyrosine-protein kinase Fer-like | 1.44 |
| LOC106582182 | XM_014164995.1 | E3 SUMO-protein ligase RanBP2-like | 1.44 |
| LOC106560827 | XM_014124168.1 | ran-binding protein 10-like | 1.44 |
| ralbp1 | XM_014141319.1 | ralA binding protein 1 | 1.44 |
| LOC106563875 | XM_014129799.1 | protocadherin-16-like | 1.44 |
| LOC106600808 | XM_014192497.1 | zinc finger protein 91-like | 1.44 |
| LOC100380858 | XM_014172839.1 | E3 ubiquitin-protein ligase MYCBP2 | 1.44 |
| LOC106582213 | XM_014165061.1 | E3 SUMO-protein ligase RanBP2-like | 1.44 |
| LOC106575498 | XM_014152012.1 | probable ubiquitin carboxyl-terminal hydrolase FAF-X | 1.44 |
| LOC106612937 | XM_014214612.1 | multidrug and toxin extrusion protein 1-like | 1.44 |
| LOC106608394 | XM_014206306.1 | zinc finger MIZ domain-containing protein 1-like | 1.44 |
| bahcc1 | XM_014179743.1 | BAH domain and coiled-coil containing 1 | 1.44 |
| LOC106612256 | XM_014213255.1 | proton-coupled amino acid transporter 1-like | 1.44 |
| LOC106608676 | XM_014206776.1 | DNA-directed RNA polymerase II subunit RPB1-like | 1.44 |
| LOC106562962 | XM_014128097.1 | collagen alpha-1(V) chain-like | 1.44 |
| LOC106588182 | XM_014176914.1 | histone-lysine N-methyltransferase ASH1L-like | 1.44 |
| LOC106585522 | XM_014171824.1 | E1A-binding protein p400-like | 1.43 |
| daam1 | XM_014211140.1 | dishevelled associated activator of morphogenesis 1 | 1.43 |
| LOC106605802 | XM_014201748.1 | regulation of nuclear pre-mRNA domain-containing protein 2-like | 1.43 |
| LOC106566627 | XM_014134877.1 | arf-GAP with SH3 domain%2C ANK repeat and PH domain-containing protein 2-like | 1.43 |
| LOC106590504 | XM_014181589.1 | thyroid hormone receptor-associated protein 3-like | 1.43 |
| LOC106599001 | XM_014190038.1 | microtubule-associated serine/threonine-protein kinase 3-like | 1.43 |
| LOC106593948 | XM_014185325.1 | uncharacterized LOC106593948 | 1.43 |
| LOC106563729 | XM_014129555.1 | amyloid beta A4 precursor protein-binding family B member 3-like | 1.43 |
| LOC106604257 | XM_014198738.1 | rho-related GTP-binding protein RhoG-like | 1.43 |
| gpr18 | XM_014163774.1 | G protein-coupled receptor 18 | 1.43 |
| LOC106576468 | XM_014153670.1 | adiponectin receptor protein 2-like | 1.43 |
| LOC106589933 | XM_014180385.1 | calcineurin subunit B type 1-like | 1.42 |
| LOC106610929 | XM_014210609.1 | gamma-aminobutyric acid receptor subunit beta-1-like | 1.42 |
| LOC106613135 | XM_014215095.1 | ephrin type-A receptor 4-like | 1.42 |
| LOC106603151 | XM_014196451.1 | intersectin-2-like | 1.42 |
| LOC106577113 | XM_014154855.1 | echinoderm microtubule-associated protein-like 6 | 1.42 |
| LOC106597009 | XM_014188247.1 | endophilin-A1-like | 1.42 |
| LOC106562151 | XM_014126810.1 | tight junction protein ZO-1-like | 1.42 |
| LOC106613723 | XM_014216273.1 | protein strawberry notch homolog 2-like | 1.42 |
| LOC106584705 | XM_014170219.1 | transcription factor PU.1-like | 1.42 |
| LOC106584317 | XM_014169431.1 | centrosome-associated protein 350-like | 1.42 |
| LOC106606244 | XM_014202392.1 | ubiquitin carboxyl-terminal hydrolase 7-like | 1.42 |
| orai2 | XM_014197169.1 | ORAI calcium release-activated calcium modulator 2 | 1.42 |
| LOC106584620 | XM_014170105.1 | transcription factor SOX-6-like | 1.42 |
| LOC106580886 | XM_014162419.1 | uncharacterized LOC106580886 | 1.42 |
| LOC106607739 | XM_014205013.1 | DNA polymerase zeta catalytic subunit-like | 1.42 |
| LOC106583293 | XM_014167280.1 | gastrula zinc finger protein XlCGF58.1-like | 1.42 |
| LOC106586757 | XM_014174358.1 | serine/threonine-protein kinase 10-like | 1.42 |
| LOC106562326 | XM_014127130.1 | synaptotagmin-7-like | 1.42 |
| LOC106589530 | XM_014179626.1 | CREB-binding protein-like | 1.42 |
| LOC106565100 | XM_014131804.1 | pleckstrin homology domain-containing family A member 6-like | 1.42 |
| LOC100380641 | XM_014147119.1 | E3 ubiquitin-protein ligase HUWE1 | 1.42 |
| LOC106568668 | XM_014139191.1 | FAST kinase domain-containing protein 3-like | 1.41 |
| LOC106606113 | XM_014202234.1 | SNF-related serine/threonine-protein kinase-like | 1.41 |
| LOC106560231 | XM_014122872.1 | ubiquitin carboxyl-terminal hydrolase 24-like | 1.41 |
| LOC106600551 | XM_014191988.1 | serine/threonine-protein kinase OSR1-like | 1.41 |
| kbtbd4 | XM_014126108.1 | kelch repeat and BTB (POZ) domain containing 4 | 1.41 |
| LOC106566213 | XM_014134088.1 | protein VPRBP-like | 1.41 |
| LOC106585556 | XM_014171906.1 | dihydropyrimidinase-related protein 2-like | 1.41 |
| LOC106587652 | XM_014176230.1 | paired amphipathic helix protein Sin3a-like | 1.41 |
| LOC106588461 | XM_014177495.1 | microtubule-actin cross-linking factor 1-like | 1.41 |
| LOC106595592 | XM_014186959.1 | SCY1-like protein 2 | 1.41 |
| LOC106565273 | XM_014132192.1 | ral GTPase-activating protein subunit beta-like | 1.41 |
| LOC106586144 | XM_014173048.1 | RNA-binding protein 34-like | 1.40 |
| herc2 | XM_014215599.1 | HECT and RLD domain containing E3 ubiquitin protein ligase 2 | 1.40 |
| ncor1 | XM_014137920.1 | nuclear receptor corepressor 1 | 1.40 |
| ankrd12 | XM_014142934.1 | ankyrin repeat domain 12 | 1.40 |
| LOC106560257 | XM_014122934.1 | protein furry homolog-like | 1.40 |
| LOC106604099 | XM_014198491.1 | transcriptional regulator ATRX-like | 1.40 |
| LOC106607615 | XM_014204761.1 | protein FAM149B1-like | 1.40 |
| LOC106603121 | XM_014196383.1 | sodium/potassium/calcium exchanger 3-like | 1.40 |
| LOC106566780 | XM_014135168.1 | cadherin EGF LAG seven-pass G-type receptor 3-like | 1.40 |
| LOC106576747 | XM_014154115.1 | echinoderm microtubule-associated protein-like 4 | 1.40 |
| LOC106566398 | XM_014134378.1 | zinc finger protein 335-like | 1.40 |
| LOC106571127 | XM_014143830.1 | kinesin-like protein KIF26A | 1.40 |
| LOC106567026 | XM_014135807.1 | E3 ubiquitin-protein ligase HUWE1-like | 1.40 |
| LOC106606345 | XM_014202500.1 | protein NLRC3-like | 1.40 |
| LOC106613785 | XM_014216397.1 | single-stranded DNA-binding protein 3-like | 1.40 |
| pi4ka | XM_014127721.1 | phosphatidylinositol 4-kinase%2C catalytic%2C alpha | 1.39 |
| atl1 | XM_014194332.1 | atlastin GTPase 1 | 1.39 |
| LOC106607387 | XM_014204302.1 | dedicator of cytokinesis protein 7-like | 1.39 |
| LOC106581247 | XM_014163167.1 | unconventional myosin-XVIIIa-like | 1.39 |
| LOC106571938 | XM_014145520.1 | bromodomain adjacent to zinc finger domain protein 2A-like | 1.39 |
| LOC106586745 | XM_014174319.1 | DDB1- and CUL4-associated factor 6-like | 1.39 |
| celsr1 | XM_014170066.1 | cadherin%2C EGF LAG seven-pass G-type receptor 1 | 1.39 |
| LOC106574232 | XM_014149951.1 | lymphocyte antigen 75-like | 1.39 |
| mdn1 | XM_014204960.1 | midasin AAA ATPase 1 | 1.39 |
| LOC106585779 | XM_014172394.1 | ubiquitin carboxyl-terminal hydrolase 34-like | 1.39 |
| LOC106579993 | XM_014160495.1 | voltage-dependent N-type calcium channel subunit alpha-1B-like | 1.39 |
| LOC106574324 | XM_014150166.1 | ATP-binding cassette sub-family B member 6%2C mitochondrial-like | 1.39 |
| LOC106586434 | XM_014173706.1 | low-density lipoprotein receptor-related protein 1B-like | 1.39 |
| LOC106569317 | XM_014140546.1 | E3 ubiquitin-protein ligase mib1-like | 1.39 |
| LOC106604853 | XM_014199910.1 | carnosine synthase 1-like | 1.39 |
| LOC106608924 | XM_014207147.1 | histone acetyltransferase p300-like | 1.39 |
| LOC106601059 | XM_014192961.1 | dedicator of cytokinesis protein 7-like | 1.39 |
| LOC106607502 | XM_014204506.1 | zinc finger protein 646-like | 1.39 |
| LOC106570121 | XM_014142151.1 | histone-lysine N-methyltransferase 2B-like | 1.39 |
| LOC106613609 | XM_014216043.1 | microtubule-associated protein 1B-like | 1.39 |
| gpr19 | XM_014183397.1 | G protein-coupled receptor 19 | 1.39 |
| LOC106608644 | XM_014206720.1 | DENN domain-containing protein 4C-like | 1.39 |
| arhgap31 | XM_014129333.1 | Rho GTPase activating protein 31 | 1.39 |
| arvcf | XM_014170897.1 | armadillo repeat gene deleted in velocardiofacial syndrome | 1.39 |
| apc | XM_014126219.1 | adenomatous polyposis coli | 1.39 |
| LOC106572865 | XM_014147408.1 | sortilin-like | 1.39 |
| LOC106582376 | XM_014165433.1 | isocitrate dehydrogenase [NADP] cytoplasmic-like | 1.39 |
| LOC106583532 | XM_014167833.1 | potassium voltage-gated channel subfamily B member 1-like | 1.38 |
| LOC106580939 | XM_014162499.1 | pleckstrin homology-like domain family B member 1 | 1.38 |
| LOC106588595 | XM_014177750.1 | poly [ADP-ribose] polymerase 10-like | 1.38 |
| LOC106572710 | XM_014147116.1 | histone lysine demethylase PHF8-like | 1.38 |
| bicc1 | XM_014154784.1 | BicC family RNA binding protein 1 | 1.38 |
| LOC106610958 | XM_014210685.1 | BAG family molecular chaperone regulator 5-like | 1.38 |
| baz2b | XM_014164143.1 | bromodomain adjacent to zinc finger domain%2C 2B | 1.38 |
| LOC106577192 | XM_014155046.1 | disks large homolog 5-like | 1.38 |
| LOC106561374 | XM_014125257.1 | zinc finger protein 462-like | 1.37 |
| rb39b | NM_001140706.1 | Ras-related protein Rab-39B | 1.37 |
| camkk2 | XM_014134123.1 | calcium/calmodulin-dependent protein kinase kinase 2%2C beta | 1.37 |
| LOC106611354 | XM_014211507.1 | transcription factor HIVEP2-like | 1.37 |
| LOC106571901 | XM_014145440.1 | nuclear pore membrane glycoprotein 210-like | 1.37 |
| LOC106606894 | XM_014203350.1 | uncharacterized LOC106606894 | 1.37 |
| LOC106609167 | XM_014207647.1 | uncharacterized LOC106609167 | 1.37 |
| LOC106607945 | XM_014205458.1 | attractin-like protein 1 | 1.37 |
| LOC106609880 | XM_014208904.1 | ATP-binding cassette sub-family A member 1-like | 1.37 |
| LOC106597319 | XM_014188527.1 | serine/threonine-protein kinase PRP4 homolog | 1.37 |
| LOC106612372 | XM_014213449.1 | rho GTPase-activating protein 35-like | 1.37 |
| LOC106568905 | XM_014139698.1 | glycogen debranching enzyme-like | 1.37 |
| LOC106613304 | XM_014215396.1 | disks large homolog 5-like | 1.36 |
| LOC106587244 | XM_014175447.1 | tight junction protein ZO-1-like | 1.36 |
| celsr3 | XM_014146319.1 | cadherin%2C EGF LAG seven-pass G-type receptor 3 | 1.36 |
| LOC106608277 | XM_014206138.1 | stathmin-4-like | 1.36 |
| LOC106566935 | XM_014135550.1 | mediator of RNA polymerase II transcription subunit 13-like | 1.36 |
| LOC106572922 | XM_014147508.1 | host cell factor 1-like | 1.36 |
| setd1b | XM_014130654.1 | SET domain containing 1B | 1.36 |
| LOC106569615 | XM_014141157.1 | hippocampus abundant transcript 1 protein-like | 1.36 |
| LOC106566730 | XM_014135058.1 | death-inducer obliterator 1-like | 1.36 |
| fgfr2 | XM_014154423.1 | fibroblast growth factor receptor 2 | 1.36 |
| LOC106581949 | XM_014164507.1 | probable ubiquitin carboxyl-terminal hydrolase FAF-X | 1.36 |
| LOC106564780 | XM_014131144.1 | proteoglycan 4-like | 1.36 |
| LOC106603505 | XM_014197279.1 | nuclear receptor corepressor 1-like | 1.35 |
| LOC106573031 | XM_014147639.1 | activating molecule in BECN1-regulated autophagy protein 1-like | 1.35 |
| myo6 | XM_014204923.1 | myosin VI | 1.35 |
| LOC106567420 | XM_014136642.1 | ankyrin repeat and KH domain-containing protein 1-like | 1.35 |
| LOC106574260 | XM_014150023.1 | CLIP-associating protein 1-B-like | 1.35 |
| LOC106606839 | XM_014203218.1 | cAMP-dependent protein kinase catalytic subunit alpha | 1.34 |
| LOC106606438 | XM_014202644.1 | E3 ubiquitin-protein ligase RNF213-like | 1.34 |
| mapkapk5 | XM_014160995.1 | mitogen-activated protein kinase-activated protein kinase 5 | 1.34 |
| LOC106609404 | XM_014208197.1 | golgin subfamily B member 1-like | 1.34 |
| LOC106606187 | XM_014202318.1 | putative oxidoreductase GLYR1 | 1.34 |
| LOC106609364 | XM_014208124.1 | myotubularin-related protein 5-like | 1.34 |
| LOC106560961 | XM_014124440.1 | A-kinase anchor protein 13-like | 1.34 |
| LOC106586143 | XM_014173040.1 | glutaminase kidney isoform%2C mitochondrial-like | 1.34 |
| LOC106587346 | XM_014175667.1 | transcription factor 12-like | 1.33 |
| LOC106570019 | XM_014141900.1 | zinc finger MYM-type protein 4-like | 1.33 |
| LOC106601535 | XM_014193789.1 | histone-lysine N-methyltransferase SETD1A-like | 1.33 |
| cssa01h5orf42 | XM_014130125.1 | chromosome ssa01 open reading frame%2C human C5orf42 | 1.33 |
| akap12 | XM_014205622.1 | A kinase (PRKA) anchor protein 12 | 1.33 |
| LOC106604460 | XM_014199094.1 | mediator of RNA polymerase II transcription subunit 12-like | 1.33 |
| LOC106610401 | XM_014209766.1 | putative sodium-coupled neutral amino acid transporter 10 | 1.33 |
| LOC106588825 | XM_014178285.1 | zinc finger protein OZF-like | 1.33 |
| LOC106603093 | XM_014196293.1 | nuclear factor related to kappa-B-binding protein-like | 1.33 |
| LOC106584099 | XM_014168932.1 | uncharacterized LOC106584099 | 1.33 |
| LOC106594832 | XM_014186218.1 | mitogen-activated protein kinase 6-like | 1.33 |
| tenm3 | XM_014199200.1 | teneurin transmembrane protein 3 | 1.33 |
| LOC106577869 | XM_014156319.1 | ral GTPase-activating protein subunit alpha-2-like | 1.33 |
| LOC106573797 | XM_014149146.1 | uncharacterized LOC106573797 | 1.32 |
| LOC106582878 | XM_014166456.1 | host cell factor 1-like | 1.32 |
| LOC106590111 | XM_014180690.1 | disks large-associated protein 1-like | 1.32 |
| LOC106599852 | XM_014191197.1 | ATP-binding cassette sub-family B member 8%2C mitochondrial-like | 1.32 |
| LOC106591038 | XM_014182227.1 | transcriptional regulator ATRX-like | 1.32 |
| LOC106594851 | XM_014186236.1 | vacuolar protein-sorting-associated protein 36-like | 1.32 |
| LOC106608443 | XM_014206375.1 | zinc finger protein 638-like | 1.32 |
| LOC106577937 | XM_014156410.1 | solute carrier family 12 member 6-like | 1.32 |
| tenm2 | XM_014198165.1 | teneurin transmembrane protein 2 | 1.32 |
| kiaa1033 | XM_014169894.1 | KIAA1033 ortholog | 1.32 |
| LOC106609564 | XM_014208501.1 | UHRF1-binding protein 1-like | 1.32 |
| LOC106578687 | XM_014157789.1 | titin-like | 1.32 |
| LOC106565871 | XM_014133495.1 | leucine-rich repeats and immunoglobulin-like domains protein 2 | 1.32 |
| LOC106579475 | XM_014159419.1 | uncharacterized protein CXorf23-like | 1.32 |
| LOC106563799 | XM_014129659.1 | telomerase-binding protein EST1A-like | 1.31 |
| LOC106577550 | XM_014155668.1 | DNA-directed RNA polymerase II subunit RPB1 | 1.31 |
| dync1h1 | XM_014211261.1 | dynein%2C cytoplasmic 1%2C heavy chain 1 | 1.31 |
| LOC106575550 | XM_014152116.1 | ATP-dependent RNA helicase DDX3X-like | 1.31 |
| LOC106572432 | XM_014146609.1 | lysine-specific demethylase 5C-like | 1.31 |
| LOC106585679 | XM_014172162.1 | probable helicase with zinc finger domain | 1.31 |
| LOC106563017 | XM_014128196.1 | microtubule-associated serine/threonine-protein kinase 1-like | 1.31 |
| zfhx2 | XM_014181083.1 | zinc finger homeobox 2 | 1.31 |
| LOC106578096 | XM_014156690.1 | uncharacterized LOC106578096 | 1.31 |
| LOC106569888 | XM_014141603.1 | uncharacterized LOC106569888 | 1.30 |
| LOC106603594 | XM_014197462.1 | zinc finger protein 287-like | 1.30 |
| LOC106584163 | XM_014169101.1 | uncharacterized protein KIAA1107-like | 1.30 |
| atg2b | XM_014182999.1 | autophagy related 2B | 1.30 |
| LOC106570138 | XM_014142175.1 | protein capicua homolog | 1.30 |
| LOC106567000 | XM_014135743.1 | disco-interacting protein 2 homolog B-A | 1.29 |
| LOC106583438 | XM_014167597.1 | helicase ARIP4-like | 1.29 |
| cep152 | XM_014126006.1 | centrosomal protein 152kDa | 1.29 |
| LOC106563574 | XM_014129280.1 | APC membrane recruitment protein 2-like | 1.29 |
| LOC106579416 | XM_014159306.1 | glutamine and serine-rich protein 1-like | 1.29 |
| LOC106563499 | XM_014129150.1 | leucine-rich repeat and fibronectin type-III domain-containing protein 4-like | 1.29 |
| LOC106605930 | XM_014201937.1 | cullin-9-like | 1.29 |
| tsc1 | XM_014174820.1 | tuberous sclerosis 1 | 1.29 |
| LOC106580217 | XM_014161045.1 | zinc finger CCCH domain-containing protein 13-like | 1.29 |
| prkar1a | XM_014179783.1 | protein kinase%2C cAMP-dependent%2C regulatory%2C type I%2C alpha | 1.29 |
| LOC106580171 | XM_014160933.1 | glutamate receptor ionotropic%2C delta-2-like | 1.28 |
| LOC106605771 | XM_014201700.1 | uncharacterized LOC106605771 | 1.28 |
| LOC106608118 | XM_014205839.1 | serine/threonine-protein kinase MRCK alpha-like | 1.28 |
| LOC106603445 | XM_014197130.1 | E3 ubiquitin-protein ligase TTC3-like | 1.28 |
| LOC106564572 | XM_014130716.1 | choline transporter-like protein 2 | 1.28 |
| LOC106603740 | XM_014197790.1 | rho guanine nucleotide exchange factor 12-like | 1.27 |
| LOC100380626 | XM_014211175.1 | E3 ubiquitin-protein ligase HECTD1 | 1.27 |
| LOC106563530 | XM_014129206.1 | anoctamin-5-like | 1.27 |
| LOC106584401 | XM_014169676.1 | AP-3 complex subunit delta-1-like | 1.26 |
| LOC106567856 | XM_014137676.1 | transcription initiation factor TFIID subunit 1-like | 1.25 |
| LOC106607579 | XM_014204654.1 | ELM2 and SANT domain-containing protein 1-like | 1.24 |
| LOC106561878 | XM_014126152.1 | bifunctional glutamate/proline--tRNA ligase-like | 1.24 |
| arhgap5 | XM_014194348.1 | Rho GTPase activating protein 5 | 1.24 |
| LOC106566086 | XM_014133916.1 | protein FAM208A-like | 1.23 |
| LOC106590587 | XM_014181700.1 | E3 ubiquitin-protein ligase MARCH6-like | 1.23 |
| LOC106563887 | XM_014129821.1 | nipped-B-like protein | 1.22 |
| LOC106605925 | XM_014201929.1 | uncharacterized LOC106605925 | 1.21 |

**Table S4.** Downregulated (false discovery rate (FDR) < 0.01) genes in Atlantic salmon juveniles subjected to 8 weeks of exercise, compared to unexercised controls, expressed as fold change (FC) differences.

| Gene name | Transcript ID | Product | FC |
| --- | --- | --- | --- |
| LOC106576770 | XM_014154148.1 | placenta-specific protein 9-like | 2.24 |
| LOC106567285 | XM_014136415.1 | protein phosphatase 1 regulatory subunit 12B-like | 2.17 |
| LOC106597403 | XM_014188595.1 | 60S ribosomal protein L30-like | 2.06 |
| LOC106567650 | XM_014137192.1 | interleukin-31 receptor subunit alpha-like | 2.06 |
| LOC106605470 | XM_014201175.1 | calbindin-like | 2.05 |
| ier2 | NM_001140121.1 | immediate early response 2 | 2.04 |
| ccd93 | NM_001140793.1 | Coiled-coil domain-containing protein 93 | 2.01 |
| gmnn | XM_014178281.1 | geminin%2C DNA replication inhibitor | 2.01 |
| LOC106600784 | XM_014192436.1 | uncharacterized LOC106600784 | 2.01 |
| LOC106607261 | XM_014204054.1 | uncharacterized LOC106607261 | 2.00 |
| LOC106595025 | XM_014186406.1 | uncharacterized LOC106595025 | 1.97 |
| foxn4 | XM_014160780.1 | forkhead box N4 | 1.94 |
| LOC106586018 | XM_014172799.1 | P2Y purinoceptor 8-like | 1.87 |
| mrps25 | XM_014134839.1 | mitochondrial ribosomal protein S25 | 1.82 |
| tprkb | NM_001146530.1 | TP53RK binding protein | 1.79 |
| LOC106560857 | XM_014124239.1 | testican-2-like | 1.77 |
| LOC106601135 | XM_014193096.1 | formin-like protein 1 | 1.77 |
| LOC106573097 | XM_014147799.1 | sorting nexin-10B-like | 1.76 |
| arhgap22 | XM_014198771.1 | Rho GTPase activating protein 22 | 1.74 |
| LOC106577931 | XM_014156403.1 | gamma-secretase subunit PEN-2 | 1.74 |
| LOC106610322 | XM_014209609.1 | NADH dehydrogenase [ubiquinone] 1 beta subcomplex subunit 6-like | 1.74 |
| LOC106566918 | XM_014135522.1 | SAM pointed domain-containing Ets transcription factor-like | 1.73 |
| LOC106595643 | XM_014187007.1 | uncharacterized protein C11orf98 homolog | 1.72 |
| LOC106609350 | XM_014208095.1 | PHD finger-like domain-containing protein 5A | 1.71 |
| anc13 | NM_001146659.1 | Anaphase-promoting complex subunit 13 | 1.71 |
| LOC106610173 | XM_014209364.1 | polypeptide N-acetylgalactosaminyltransferase-like 6 | 1.71 |
| LOC106570689 | XM_014143162.1 | MARCKS-related protein-like | 1.70 |
| LOC106572841 | XM_014147371.1 | small nuclear ribonucleoprotein G | 1.70 |
| LOC106579659 | XM_014159793.1 | GTP-binding protein Rheb | 1.70 |
| LOC106581057 | XM_014162767.1 | coiled-coil-helix-coiled-coil-helix domain-containing protein 2-like | 1.69 |
| LOC106566856 | XM_014135351.1 | succinate dehydrogenase [ubiquinone] iron-sulfur subunit%2C mitochondrial-like | 1.69 |
| LOC106598275 | XM_014189325.1 | G patch domain and KOW motifs-containing protein-like | 1.69 |
| LOC106605408 | XM_014201013.1 | zinc-binding protein A33-like | 1.68 |
| LOC106609479 | XM_014208352.1 | small nuclear ribonucleoprotein F | 1.68 |
| LOC106567806 | XM_014137561.1 | non-histone chromosomal protein HMG-14A-like | 1.68 |
| LOC106607185 | XM_014203875.1 | von Willebrand factor C domain-containing protein 2-like | 1.68 |
| LOC106601291 | XM_014193395.1 | growth arrest and DNA damage-inducible proteins-interacting protein 1-like | 1.67 |
| LOC106604695 | XM_014199616.1 | short coiled-coil protein B-like | 1.67 |
| LOC106566187 | XM_014134060.1 | translation machinery-associated protein 7 | 1.66 |
| LOC106591659 | XM_014182884.1 | NADH dehydrogenase [ubiquinone] 1 beta subcomplex subunit 7-like | 1.66 |
| LOC106602949 | XM_014195997.1 | protein CXorf40A-like | 1.66 |
| LOC106609122 | XM_014207571.1 | vesicle transport protein GOT1B-like | 1.65 |
| LOC106565911 | XM_014133598.1 | mRNA turnover protein 4 homolog | 1.65 |
| LOC106593061 | XM_014184408.1 | meteorin-like protein | 1.65 |
| LOC106601365 | XM_014193542.1 | transcription elongation factor 1 homolog | 1.64 |
| LOC106600265 | XM_014191583.1 | NEDD8-like | 1.64 |
| ndub7 | NM_001140935.1 | NADH dehydrogenase 1 beta subcomplex subunit 7 | 1.64 |
| LOC106583717 | XM_014168243.1 | translation machinery-associated protein 7-like | 1.63 |
| LOC106565650 | XM_014132981.1 | MICOS complex subunit MIC10-like | 1.63 |
| LOC106566032 | XM_014133838.1 | 60S acidic ribosomal protein P2-like | 1.63 |
| LOC106560581 | XM_014123596.1 | prothymosin alpha-A-like | 1.63 |
| LOC106561290 | XM_014125077.1 | uncharacterized LOC106561290 | 1.63 |
| LOC106565357 | XM_014132369.1 | mitochondrial import receptor subunit TOM6 homolog | 1.63 |
| LOC106605614 | XM_014201448.1 | gamma-secretase subunit PEN-2-like | 1.62 |
| LOC106573476 | XM_014148563.1 | proteoglycan 4-like | 1.62 |
| LOC106581904 | XM_014164352.1 | NADH dehydrogenase [ubiquinone] flavoprotein 3%2C mitochondrial-like | 1.62 |
| LOC106570471 | XM_014142851.1 | 26S proteasome complex subunit DSS1-like | 1.62 |
| LOC106568858 | XM_014139592.1 | integral membrane protein 2C-like | 1.61 |
| rl23a | NM_001141301.1 | 60S ribosomal protein L23a | 1.61 |
| LOC106606605 | XM_014202931.1 | hematological and neurological expressed 1 protein-like | 1.61 |
| LOC106608853 | XM_014207032.1 | homeobox and leucine zipper protein Homez-like | 1.61 |
| lsm5 | NM_001146660.1 | U6 snRNA-associated Sm-like protein LSm5 | 1.61 |
| mak16 | XM_014174880.1 | MAK16 homolog | 1.61 |
| LOC106601801 | XM_014194188.1 | hematological and neurological expressed 1 protein-like | 1.60 |
| LOC106576397 | XM_014153539.1 | 39S ribosomal protein L42%2C mitochondrial-like | 1.60 |
| srek1ip1 | XM_014126946.1 | SREK1-interacting protein 1 | 1.60 |
| LOC106562913 | XM_014128007.1 | histone H2A.V | 1.60 |
| LOC106586742 | XM_014174318.1 | cytochrome c oxidase assembly factor 5 | 1.59 |
| LOC106582218 | XM_014165073.1 | stress-associated endoplasmic reticulum protein 2 | 1.59 |
| LOC106585031 | XM_014170775.1 | 28S ribosomal protein S36%2C mitochondrial-like | 1.59 |
| LOC106561682 | XM_014125865.1 | small nuclear ribonucleoprotein F-like | 1.59 |
| LOC106611745 | XM_014212286.1 | cysteine-rich hydrophobic domain-containing protein 1-like | 1.59 |
| ppdpf | NM_001139699.1 | c20orf149 protein | 1.58 |
| cxxc5 | XM_014212997.1 | CXXC finger protein 5 | 1.58 |
| ca108 | NM_001140609.2 | CA108 protein | 1.58 |
| LOC106600555 | XM_014192003.1 | NADH dehydrogenase [ubiquinone] 1 alpha subcomplex subunit 13-like | 1.58 |
| LOC106561079 | XM_014124701.1 | 40S ribosomal protein S13 | 1.58 |
| LOC106598980 | XM_014190015.1 | 40S ribosomal protein S28 | 1.58 |
| LOC106584327 | XM_014169486.1 | stathmin-like | 1.58 |
| LOC106611775 | XM_014212334.1 | short coiled-coil protein B-like | 1.57 |
| dnajc12 | NM_001141838.1 | DnaJ (Hsp40) homolog%2C subfamily C%2C member 12 | 1.57 |
| hn1 | NM_001123720.1 | hematological and neurological expressed 1 | 1.57 |
| LOC106581844 | XM_014164255.1 | RNA-binding protein 8A | 1.56 |
| cssa15h14orf2 | XM_014144682.1 | chromosome ssa15 open reading frame%2C human C14orf2 | 1.56 |
| dynlt3 | XM_014174208.1 | dynein%2C light chain%2C Tctex-type 3 | 1.56 |
| LOC106583768 | XM_014168332.1 | transcription and mRNA export factor ENY2-1 | 1.56 |
| LOC106611096 | XM_014210963.1 | proto-oncogene c-Fos-like | 1.56 |
| LOC106588702 | XM_014177965.1 | NADH dehydrogenase [ubiquinone] iron-sulfur protein 6%2C mitochondrial-like | 1.56 |
| ndub6 | NM_001140996.2 | NADH dehydrogenase 1 beta subcomplex subunit 6 | 1.55 |
| LOC106609885 | XM_014208916.1 | complexin-1-like | 1.55 |
| hint1 | NM_001141156.1 | histidine triad nucleotide binding protein 1 | 1.55 |
| LOC106573685 | XM_014149000.1 | cytochrome c oxidase subunit 7A2%2C mitochondrial-like | 1.55 |
| LOC106583512 | XM_014167784.1 | mitotic-spindle organizing protein 1 | 1.55 |
| LOC106599020 | XM_014190066.1 | transcription factor jun-D-like | 1.55 |
| lsm3 | NM_001146369.1 | LSM3 homolog%2C U6 small nuclear RNA and mRNA degradation associated | 1.55 |
| atp5e | XM_014145574.1 | ATP synthase%2C H+ transporting%2C mitochondrial F1 complex%2C epsilon subunit | 1.55 |
| LOC106594622 | XM_014186029.1 | ubiquitin-like protein 5 | 1.55 |
| ct011 | NM_001140653.1 | CT011 protein | 1.55 |
| LOC106579627 | XM_014159725.1 | programmed cell death protein 6-like | 1.55 |
| LOC106596057 | XM_014187377.1 | NADH dehydrogenase [ubiquinone] 1 beta subcomplex subunit 8%2C mitochondrial-like | 1.55 |
| ofd1 | XM_014164391.1 | oral-facial-digital syndrome 1 | 1.55 |
| LOC106571604 | XM_014144864.1 | splicing factor 3B subunit 6 | 1.55 |
| LOC106583016 | XM_014166754.1 | signal peptidase complex subunit 1-like | 1.54 |
| LOC106607575 | XM_014204646.1 | zinc finger CCHC domain-containing protein 10-like | 1.54 |
| LOC106611374 | XM_014211536.1 | SRA stem-loop-interacting RNA-binding protein%2C mitochondrial-like | 1.54 |
| LOC106577308 | XM_014155256.1 | NADH dehydrogenase [ubiquinone] 1 beta subcomplex subunit 8%2C mitochondrial-like | 1.53 |
| fundc2 | XM_014129171.1 | FUN14 domain containing 2 | 1.53 |
| LOC106604773 | XM_014199794.1 | 39S ribosomal protein L18%2C mitochondrial-like | 1.53 |
| LOC106572526 | XM_014146767.1 | zinc transporter ZIP10-like | 1.53 |
| LOC106604775 | XM_014199797.1 | mitochondrial import receptor subunit TOM5 homolog | 1.53 |
| LOC106581122 | XM_014162880.1 | lebercilin-like protein | 1.53 |
| pde6d | XM_014168616.1 | phosphodiesterase 6D%2C cGMP-specific%2C rod%2C delta | 1.53 |
| LOC106560579 | XM_014123592.1 | retinal rod rhodopsin-sensitive cGMP 3'%2C5'-cyclic phosphodiesterase subunit delta | 1.53 |
| ndub4 | NM_001141168.1 | NADH dehydrogenase 1 beta subcomplex subunit 4 | 1.53 |
| LOC106610070 | XM_014209209.1 | parvalbumin%2C thymic CPV3-like | 1.53 |
| LOC106603453 | XM_014197161.1 | derlin-2-like | 1.53 |
| LOC106607688 | XM_014204900.1 | cytochrome c oxidase subunit 7A2%2C mitochondrial | 1.53 |
| LOC106598901 | XM_014189920.1 | 15 kDa selenoprotein-like | 1.53 |
| LOC106601894 | XM_014194324.1 | jmjC domain-containing protein 8-like | 1.53 |
| LOC106603327 | XM_014196867.1 | non-histone chromosomal protein HMG-14A-like | 1.53 |
| LOC106564190 | XM_014130228.1 | DBIRD complex subunit ZNF326-like | 1.53 |
| LOC106602281 | XM_014194812.1 | NADH dehydrogenase [ubiquinone] 1 beta subcomplex subunit 6-like | 1.53 |
| LOC106605008 | XM_014200198.1 | stathmin-like | 1.53 |
| LOC106572941 | XM_014147534.1 | neuropeptides B/W receptor type 2-like | 1.53 |
| LOC106600926 | XM_014192717.1 | protein PET100 homolog%2C mitochondrial-like | 1.52 |
| cplx1 | NM_001140038.1 | Complexin-1 | 1.52 |
| LOC106576359 | XM_014153479.1 | NADH dehydrogenase [ubiquinone] 1 beta subcomplex subunit 2%2C mitochondrial-like | 1.52 |
| ube2v2 | XM_014191134.1 | ubiquitin-conjugating enzyme E2 variant 2 | 1.52 |
| LOC106566142 | XM_014133992.1 | dynactin subunit 2-like | 1.52 |
| LOC106583191 | XM_014167096.1 | probable protein BRICK1 | 1.52 |
| lyrm4 | XM_014188368.1 | LYR motif containing 4 | 1.52 |
| LOC106592956 | XM_014184308.1 | general transcription factor IIE subunit 2-like | 1.52 |
| rs30 | NM_001146588.1 | 40S ribosomal protein S30 | 1.52 |
| LOC106571903 | XM_014145450.1 | platelet glycoprotein IX-like | 1.52 |
| LOC106589895 | XM_014180309.1 | oxidoreductase-like domain-containing protein 1 | 1.52 |
| smim15 | XM_014171061.1 | small integral membrane protein 15 | 1.51 |
| sfrs1 | NM_001173643.1 | Splicing factor%2C arginine/serine-rich 1 | 1.51 |
| LOC106600955 | XM_014192764.1 | ferritin%2C middle subunit-like | 1.51 |
| ndufb3 | NM_001146531.1 | NADH dehydrogenase (ubiquinone) 1 beta subcomplex%2C 3%2C 12kDa | 1.51 |
| LOC106579764 | XM_014159960.1 | microsomal glutathione S-transferase 3-like | 1.51 |
| pagr1 | XM_014159253.1 | PAXIP1 associated glutamate-rich protein 1 | 1.51 |
| pdyn | NM_001140923.1 | prodynorphin | 1.51 |
| LOC106609400 | XM_014208191.1 | NADH dehydrogenase [ubiquinone] 1 beta subcomplex subunit 2%2C mitochondrial-like | 1.51 |
| LOC106563115 | XM_014128385.1 | ATPase inhibitor B%2C mitochondrial-like | 1.51 |
| LOC106562465 | XM_014127362.1 | signal peptidase complex catalytic subunit SEC11A | 1.50 |
| LOC106565436 | XM_014132573.1 | SAP domain-containing ribonucleoprotein-like | 1.50 |
| LOC106589582 | XM_014179727.1 | parvalbumin%2C thymic CPV3-like | 1.50 |
| dync2li1 | XM_014210574.1 | dynein%2C cytoplasmic 2%2C light intermediate chain 1 | 1.50 |
| LOC106584719 | XM_014170241.1 | eukaryotic translation initiation factor 3 subunit J-A-like | 1.50 |
| borcs7 | XM_014154003.1 | BLOC-1 related complex subunit 7 | 1.50 |
| LOC106607317 | XM_014204160.1 | formin-like protein 1 | 1.50 |
| LOC106570481 | XM_014142876.1 | cytochrome b-c1 complex subunit 7-like | 1.50 |
| LOC106573564 | XM_014148711.1 | mitochondrial import inner membrane translocase subunit Tim13 | 1.50 |
| ovca2 | NM_001140808.1 | ovarian tumor suppressor candidate 2 | 1.49 |
| cssa13hxorf56 | XM_014137510.1 | chromosome ssa13 open reading frame%2C human CXorf56 | 1.49 |
| LOC106591465 | XM_014182702.1 | transmembrane emp24 domain-containing protein 10-like | 1.49 |
| LOC106563619 | XM_014129357.1 | uncharacterized LOC106563619 | 1.49 |
| LOC106567917 | XM_014137806.1 | non-histone chromosomal protein HMG-14-like | 1.49 |
| LOC106565916 | XM_014133614.1 | 28S ribosomal protein S16%2C mitochondrial-like | 1.49 |
| comd5 | NM_001140699.1 | COMM domain-containing protein 5 | 1.49 |
| LOC106609820 | XM_014208845.1 | transmembrane protein 60-like | 1.49 |
| dipa | NM_001146455.1 | Delta-interacting protein A | 1.49 |
| pqbp1 | NM_001146422.1 | Polyglutamine-binding protein 1 | 1.49 |
| LOC106585740 | XM_014172313.1 | uncharacterized HIT-like protein Synpcc7942_1390 | 1.49 |
| LOC106585593 | XM_014171978.1 | ubiquitin-like protein 4A-A | 1.49 |
| txd17 | NM_001141499.1 | Thioredoxin domain-containing protein 17 | 1.48 |
| LOC106600762 | XM_014192379.1 | ferritin%2C middle subunit-like | 1.48 |
| gtf2h5 | NM_001141298.1 | general transcription factor IIH%2C polypeptide 5 | 1.48 |
| LOC106610435 | XM_014209804.1 | polyadenylate-binding protein-interacting protein 2B-like | 1.48 |
| LOC106601742 | XM_014194098.1 | NHP2-like protein 1 | 1.48 |
| LOC106569998 | XM_014141834.1 | cytochrome c oxidase subunit 6B1-like | 1.48 |
| snrpc | XM_014146394.1 | small nuclear ribonucleoprotein polypeptide C | 1.48 |
| rnasek | XM_014197441.1 | ribonuclease%2C RNase K | 1.48 |
| LOC106585554 | XM_014171905.1 | transmembrane protein 230-like | 1.48 |
| LOC106569554 | XM_014141019.1 | NADH dehydrogenase [ubiquinone] 1 alpha subcomplex subunit 13-like | 1.48 |
| LOC106577450 | XM_014155436.1 | structure-specific endonuclease subunit slx1-like | 1.48 |
| LOC106575072 | XM_014151229.1 | cyclin-dependent kinase inhibitor 1B-like | 1.47 |
| LOC106564768 | XM_014131122.1 | echinoderm microtubule-associated protein-like 6 | 1.47 |
| LOC106589535 | XM_014179635.1 | cold shock domain-containing protein C2-like | 1.47 |
| LOC106561545 | XM_014125619.1 | fibroblast growth factor receptor substrate 2-like | 1.47 |
| LOC106608529 | XM_014206486.1 | bolA-like protein 2 | 1.47 |
| LOC106610705 | XM_014210203.1 | ATPase inhibitor B%2C mitochondrial-like | 1.47 |
| LOC106603561 | XM_014197413.1 | non-histone chromosomal protein HMG-14-like | 1.47 |
| LOC106573600 | XM_014148783.1 | cold-inducible RNA-binding protein B-like | 1.47 |
| LOC106586675 | XM_014174199.1 | ubiquitin-conjugating enzyme E2 A-like | 1.47 |
| vps37d | XM_014197151.1 | vacuolar protein sorting 37 homolog D (S. cerevisiae) | 1.47 |
| jmjd7 | XM_014194092.1 | jumonji domain containing 7 | 1.47 |
| tm14c | NM_001141158.1 | Transmembrane protein 14C | 1.47 |
| LOC106576117 | XM_014153006.1 | NADH dehydrogenase [ubiquinone] 1 alpha subcomplex subunit 5-like | 1.46 |
| LOC100194563 | NM_001139649.1 | mitochondrial ribosomal protein L51-like | 1.46 |
| LOC106567898 | XM_014137768.1 | ribonuclease kappa-B | 1.46 |
| lin7b | NM_001146623.1 | lin-7 homolog B (C. elegans) | 1.46 |
| LOC106590380 | XM_014181352.1 | GTP-binding protein Rheb-like | 1.46 |
| spcs3 | NM_001140682.2 | signal peptidase complex subunit 3 homolog (S. cerevisiae) | 1.46 |
| LOC106587964 | XM_014176624.1 | heat shock factor-binding protein 1-like | 1.46 |
| mrpl34 | XM_014189988.1 | mitochondrial ribosomal protein L34 | 1.46 |
| LOC106569431 | XM_014140782.1 | NEDD8 | 1.46 |
| cdc26 | NM_001140845.1 | cell division cycle 26 | 1.46 |
| pfdn5 | XM_014132914.1 | prefoldin subunit 5 | 1.46 |
| LOC106587940 | XM_014176600.1 | gamma-aminobutyric acid receptor-associated protein-like 2 | 1.46 |
| LOC106562297 | XM_014127080.1 | COX assembly mitochondrial protein 2 homolog | 1.46 |
| LOC106580140 | XM_014160858.1 | cytochrome c oxidase subunit 7C%2C mitochondrial-like | 1.46 |
| LOC106588914 | XM_014178461.1 | cytochrome b-c1 complex subunit 7-like | 1.46 |
| LOC106568402 | XM_014138714.1 | activated RNA polymerase II transcriptional coactivator p15-like | 1.46 |
| LOC106584057 | XM_014168878.1 | protein mago nashi homolog | 1.46 |
| LOC106583097 | XM_014166913.1 | MICOS complex subunit Mic10-like | 1.46 |
| syub | NM_001141659.2 | Beta-synuclein | 1.46 |
| ltv1l | NM_001139624.1 | LTV1-like | 1.46 |
| ino80e | XM_014201017.1 | INO80 complex subunit E | 1.46 |
| vps25 | NM_001141281.1 | vacuolar protein sorting 25 homolog (S. cerevisiae) | 1.46 |
| pr38a | NM_001141758.1 | Pre-mRNA-splicing factor 38A | 1.46 |
| LOC106605822 | XM_014201784.1 | ubiquitin-like protein ATG12 | 1.46 |
| LOC106603165 | XM_014196473.1 | cofilin-2-like | 1.46 |
| LOC106569530 | XM_014140975.1 | cytochrome b-c1 complex subunit 10 | 1.46 |
| tmem234 | XM_014210094.1 | transmembrane protein 234 | 1.46 |
| LOC106597037 | XM_014188298.1 | putative E3 ubiquitin-protein ligase UBR7 | 1.46 |
| LOC106567640 | XM_014137170.1 | histone H2A | 1.46 |
| LOC106579227 | XM_014158931.1 | histone H3.3 | 1.46 |
| cssa10h12orf73 | XM_014124713.1 | chromosome ssa10 open reading frame%2C human C12orf73 | 1.45 |
| LOC106603170 | XM_014196484.1 | FUN14 domain-containing protein 1A-like | 1.45 |
| sc11a | NM_001141078.1 | Signal peptidase complex catalytic subunit SEC11A | 1.45 |
| LOC106593927 | XM_014185304.1 | uncharacterized LOC106593927 | 1.45 |
| LOC106579885 | XM_014160216.1 | transmembrane protein 230-like | 1.45 |
| LOC106583751 | XM_014168307.1 | mesencephalic astrocyte-derived neurotrophic factor-like | 1.45 |
| nr13 | XM_014127370.1 | Anti-apoptotic protein NR13 | 1.45 |
| LOC106587952 | XM_014176616.1 | proteasome subunit beta type-3 | 1.45 |
| LOC106583964 | XM_014168686.1 | prothymosin alpha-A-like | 1.45 |
| LOC106566149 | XM_014134004.1 | methionine--tRNA ligase%2C cytoplasmic-like | 1.45 |
| hs020 | NM_001146519.1 | HSPC020 homolog | 1.45 |
| LOC106573260 | XM_014148168.1 | protein LSM14 homolog A-like | 1.45 |
| mmab | XM_014160778.1 | methylmalonic aciduria (cobalamin deficiency) cblB type | 1.45 |
| LOC106613708 | XM_014216245.1 | NADH dehydrogenase [ubiquinone] 1 alpha subcomplex subunit 11-like | 1.45 |
| LOC106577644 | XM_014155944.1 | mitochondrial fission 1 protein-like | 1.45 |
| LOC106610231 | XM_014209448.1 | U6 snRNA-associated Sm-like protein LSm6 | 1.45 |
| comd1 | NM_001140257.1 | COMM domain-containing protein 1 | 1.45 |
| LOC106585051 | XM_014170807.1 | ubiquitin fusion degradation protein 1 homolog | 1.45 |
| LOC106608492 | XM_014206429.1 | uncharacterized LOC106608492 | 1.45 |
| hsp10 | XM_014165255.1 | heat shock protein 10 | 1.45 |
| LOC106587672 | XM_014176256.1 | uncharacterized LOC106587672 | 1.45 |
| LOC106609477 | XM_014208350.1 | NADH dehydrogenase [ubiquinone] 1 alpha subcomplex subunit 12-like | 1.45 |
| adat2 | XM_014144503.1 | adenosine deaminase%2C tRNA-specific 2 | 1.45 |
| LOC106564829 | XM_014131264.1 | mitochondrial import receptor subunit TOM20 homolog | 1.44 |
| atp5g2 | XM_014165625.1 | ATP synthase%2C H+ transporting%2C mitochondrial F0 complex%2C subunit c-2 | 1.44 |
| LOC106561701 | XM_014125913.1 | NADH dehydrogenase [ubiquinone] 1 alpha subcomplex subunit 8-like | 1.44 |
| LOC106581181 | XM_014163061.1 | dynein light chain 2%2C cytoplasmic | 1.44 |
| mettl3 | XM_014156116.1 | methyltransferase like 3 | 1.44 |
| LOC106608971 | XM_014207248.1 | H/ACA ribonucleoprotein complex subunit 3 | 1.44 |
| LOC106613740 | XM_014216304.1 | cold-inducible RNA-binding protein B-like | 1.44 |
| LOC106561931 | XM_014126337.1 | ARL14 effector protein-like | 1.44 |
| LOC106607035 | XM_014203593.1 | uncharacterized protein C19orf43-like | 1.44 |
| LOC106612309 | XM_014213353.1 | HIG1 domain family member 2A-like | 1.44 |
| LOC106588347 | XM_014177235.1 | protein AF1q-like | 1.44 |
| LOC106579190 | XM_014158859.1 | V-type proton ATPase 16 kDa proteolipid subunit | 1.44 |
| LOC106609683 | XM_014208655.1 | 26S protease regulatory subunit 7 | 1.44 |
| LOC106577802 | XM_014156158.1 | NEDD8-like | 1.44 |
| alg9 | XM_014198422.1 | ALG9%2C alpha-1%2C2-mannosyltransferase | 1.44 |
| LOC106611995 | XM_014212750.1 | ras-related protein Rab-33A-like | 1.44 |
| LOC106601332 | XM_014193463.1 | uncharacterized protein C19orf43-like | 1.44 |
| LOC106582252 | XM_014165136.1 | 60S ribosomal protein L31 | 1.44 |
| mrpl10 | XM_014159129.1 | mitochondrial ribosomal protein L10 | 1.44 |
| psme3 | XM_014158502.1 | proteasome activator subunit 3 | 1.44 |
| LOC106600912 | XM_014192682.1 | gastrula zinc finger protein xFG20-1-like | 1.44 |
| LOC106563057 | XM_014128278.1 | cytochrome c oxidase subunit 5B%2C mitochondrial-like | 1.44 |
| pold4 | NM_001146625.1 | polymerase (DNA-directed)%2C delta 4%2C accessory subunit | 1.44 |
| t4s5 | NM_001141538.1 | Transmembrane 4 L6 family member 5 | 1.43 |
| eif3g | NM_001140944.1 | eukaryotic translation initiation factor 3%2C subunit G | 1.43 |
| LOC106590149 | XM_014180800.1 | charged multivesicular body protein 5 | 1.43 |
| abracl | XM_014204735.1 | ABRA C-terminal like | 1.43 |
| LOC106604953 | XM_014200117.1 | histone deacetylase 3 | 1.43 |
| pfd2 | NM_001141000.2 | Prefoldin subunit 2 | 1.43 |
| LOC106567989 | XM_014137953.1 | cytochrome c oxidase subunit 5B%2C mitochondrial-like | 1.43 |
| ube2c | XM_014168153.1 | ubiquitin-conjugating enzyme E2C | 1.43 |
| LOC106582960 | XM_014166607.1 | mRNA turnover protein 4 homolog | 1.43 |
| LOC106574023 | XM_014149533.1 | transcription factor BTF3 homolog 4 | 1.43 |
| LOC106578873 | XM_014158110.1 | histone H3.3 | 1.43 |
| LOC106567817 | XM_014137580.1 | uncharacterized LOC106567817 | 1.43 |
| LOC106583697 | XM_014168196.1 | cytochrome c oxidase assembly protein COX14 homolog | 1.43 |
| LOC106577457 | XM_014155453.1 | 60S ribosomal protein L34 | 1.43 |
| tcp4 | NM_001141005.2 | Activated RNA polymerase II transcriptional coactivator p15 | 1.43 |
| LOC106578447 | XM_014157248.1 | NADH dehydrogenase [ubiquinone] 1 beta subcomplex subunit 4-like | 1.43 |
| pgrmc1 | NM_001146359.1 | progesterone receptor membrane component 1 | 1.43 |
| LOC106611239 | XM_014211237.1 | proteasome subunit alpha type-3 | 1.43 |
| aurkaip1 | NM_001141133.1 | aurora kinase A interacting protein 1 | 1.42 |
| LOC106568316 | XM_014138546.1 | mitotic-spindle organizing protein 2-like | 1.42 |
| LOC106587263 | XM_014175499.1 | embryonic polyadenylate-binding protein 2-B-like | 1.42 |
| LOC106608255 | XM_014206107.1 | ER membrane protein complex subunit 7-like | 1.42 |
| LOC106568198 | XM_014138326.1 | cytochrome b-c1 complex subunit 9-like | 1.42 |
| LOC106562909 | XM_014127997.1 | 28S ribosomal protein S24%2C mitochondrial-like | 1.42 |
| ndua4 | NM_001146612.1 | NADH dehydrogenase 1 alpha subcomplex subunit 4 | 1.42 |
| i2c2 | NM_001141115.1 | Eukaryotic translation initiation factor 2C 2 | 1.42 |
| LOC106582745 | XM_014166132.1 | mitochondrial import inner membrane translocase subunit Tim17-A | 1.42 |
| cssa24h22orf39 | XM_014170809.1 | chromosome ssa24 open reading frame%2C human C22orf39 | 1.42 |
| fam32a | NM_001140918.1 | family with sequence similarity 32%2C member A | 1.42 |
| LOC106605735 | XM_014201635.1 | inhibitor of growth protein 4-like | 1.42 |
| LOC106574050 | XM_014149576.1 | zinc finger protein 287-like | 1.42 |
| ramp3 | NM_001141893.1 | Receptor activity-modifying protein 3 | 1.42 |
| ck046 | XM_014174760.1 | CK046 protein | 1.42 |
| wbscr22 | XM_014215407.1 | Williams Beuren syndrome chromosome region 22 | 1.42 |
| LOC106585463 | XM_014171689.1 | small nuclear ribonucleoprotein Sm D3-like | 1.42 |
| LOC106584048 | XM_014168858.1 | UPF0690 protein C1orf52 homolog | 1.42 |
| LOC106604860 | XM_014199931.1 | zinc finger protein 432-like | 1.42 |
| dyl1 | NM_001141596.1 | Dynein light chain 1%2C cytoplasmic | 1.41 |
| LOC106581973 | XM_014164576.1 | DCN1-like protein 2 | 1.41 |
| LOC106577556 | XM_014155699.1 | eukaryotic translation initiation factor 5A-1 | 1.41 |
| LOC106563983 | XM_014129939.1 | actin-related protein 2/3 complex subunit 1A-like | 1.41 |
| LOC106563483 | XM_014129114.1 | barrier-to-autointegration factor-like | 1.41 |
| frih | NM_001146488.1 | Ferritin%2C heavy subunit | 1.41 |
| LOC106581107 | XM_014162851.1 | small nuclear ribonucleoprotein Sm D2 | 1.41 |
| LOC106563745 | XM_014129578.1 | NADH dehydrogenase [ubiquinone] 1 alpha subcomplex subunit 2-like | 1.41 |
| LOC106603050 | XM_014196186.1 | transgelin-3-like | 1.41 |
| LOC106591262 | XM_014182479.1 | uncharacterized LOC106591262 | 1.41 |
| LOC106580809 | XM_014162266.1 | neuromodulin-like | 1.41 |
| cssa07h4orf48 | NM_001140874.1 | chromosome ssa07 open reading frame%2C human C4orf48 | 1.41 |
| LOC106601603 | XM_014193936.1 | cytochrome c oxidase subunit NDUFA4-like | 1.41 |
| LOC106580840 | XM_014162319.1 | histone H3.3 | 1.41 |
| LOC106568726 | XM_014139313.1 | plasminogen activator inhibitor 1 RNA-binding protein-like | 1.41 |
| LOC106565348 | XM_014132347.1 | serine/arginine-rich splicing factor 3-like | 1.41 |
| djb12 | NM_001140370.1 | DnaJ homolog subfamily B member 12 | 1.41 |
| LOC106562262 | XM_014127029.1 | methenyltetrahydrofolate synthase domain-containing protein-like | 1.41 |
| LOC106588646 | XM_014177828.1 | stathmin-like | 1.41 |
| LOC106581902 | XM_014164346.1 | fibrous sheath CABYR-binding protein-like | 1.41 |
| LOC106611705 | XM_014212192.1 | ubiquitin-conjugating enzyme E2 D2-like | 1.41 |
| mrps6 | XM_014157989.1 | mitochondrial ribosomal protein S6 | 1.40 |
| pgls | NM_001146596.1 | 6-phosphogluconolactonase | 1.40 |
| LOC106579096 | XM_014158646.1 | 26S protease regulatory subunit 8 | 1.40 |
| LOC106573541 | XM_014148670.1 | 39S ribosomal protein L54%2C mitochondrial-like | 1.40 |
| LOC106593758 | XM_014185135.1 | THUMP domain-containing protein 1-like | 1.40 |
| LOC106583886 | XM_014168511.1 | V-type proton ATPase 16 kDa proteolipid subunit | 1.40 |
| ube2e3 | XM_014173635.1 | ubiquitin-conjugating enzyme E2E 3 (UBC4/5 homolog%2C yeast) | 1.40 |
| timm9 | XM_014194728.1 | translocase of inner mitochondrial membrane 9 homolog (yeast) | 1.40 |
| LOC106609414 | XM_014208217.1 | 28 kDa heat- and acid-stable phosphoprotein-like | 1.40 |
| rbm8a | NM_001141696.1 | RNA binding motif protein 8A | 1.40 |
| LOC106571302 | XM_014144212.1 | nucleoporin Nup43-like | 1.40 |
| tceb1 | XM_014159901.1 | transcription elongation factor B (SIII)%2C polypeptide 1 (15kDa%2C elongin C) | 1.40 |
| LOC106587995 | XM_014176656.1 | transcription initiation factor IIA subunit 2 | 1.40 |
| LOC106573597 | XM_014148774.1 | cold-inducible RNA-binding protein B-like | 1.40 |
| LOC106589607 | XM_014179790.1 | NHP2-like protein 1 | 1.40 |
| mtap | NM_001140147.1 | methylthioadenosine phosphorylase | 1.40 |
| LOC106612087 | XM_014212927.1 | UBX domain-containing protein 1-like | 1.40 |
| LOC106600830 | XM_014192534.1 | ATP synthase F(0) complex subunit C3%2C mitochondrial-like | 1.40 |
| LOC106589005 | XM_014178626.1 | proteasome subunit alpha type-2-like | 1.40 |
| psmb6 | XM_014128982.1 | proteasome subunit beta 6 | 1.40 |
| LOC106570752 | XM_014143238.1 | OTU domain-containing protein 6B-like | 1.40 |
| LOC106612273 | XM_014213289.1 | DNA-directed RNA polymerase II subunit RPB7 | 1.40 |
| LOC106601222 | XM_014193251.1 | DNA-directed RNA polymerases I%2C II%2C and III subunit RPABC2 | 1.40 |
| ppp1cb | NM_001141688.1 | protein phosphatase 1%2C catalytic subunit%2C beta isoform | 1.40 |
| LOC106587370 | XM_014175713.1 | cytochrome c oxidase subunit 4 isoform 1%2C mitochondrial-like | 1.40 |
| sdhaf2 | XM_014175961.1 | succinate dehydrogenase complex assembly factor 2 | 1.40 |
| LOC106604523 | XM_014199210.1 | CDK2-associated and cullin domain-containing protein 1-like | 1.40 |
| gbp | NM_001279048.1 | GSK-3-binding protein | 1.40 |
| LOC106590036 | XM_014180529.1 | zinc finger CCCH domain-containing protein 15-like | 1.40 |
| LOC106599988 | XM_014191321.1 | splicing factor 3A subunit 2-like | 1.40 |
| LOC106594183 | XM_014185549.1 | tax1-binding protein 1 homolog B-like | 1.40 |
| LOC106563255 | XM_014128673.1 | endothelial differentiation-related factor 1 homolog | 1.40 |
| mrps17 | XM_014195738.1 | mitochondrial ribosomal protein S17 | 1.40 |
| LOC106589850 | XM_014180250.1 | 39S ribosomal protein L27%2C mitochondrial-like | 1.39 |
| rbm17 | XM_014207890.1 | RNA binding motif protein 17 | 1.39 |
| LOC100194632 | NM_001139717.1 | endothelial differentiation-related factor 1-1 | 1.39 |
| commd6 | NM_001141062.1 | COMM domain containing 6 | 1.39 |
| LOC106609143 | XM_014207621.1 | ragulator complex protein LAMTOR4 | 1.39 |
| cnbp | NM_001139745.1 | CCHC-type zinc finger%2C nucleic acid binding protein | 1.39 |
| LOC106566440 | XM_014134478.1 | peptidyl-prolyl cis-trans isomerase FKBP1A-like | 1.39 |
| LOC106596537 | XM_014187819.1 | U6 snRNA-associated Sm-like protein LSm8 | 1.39 |
| LOC106582700 | XM_014166019.1 | U1 small nuclear ribonucleoprotein C-like | 1.39 |
| pfd1 | NM_001141510.1 | Prefoldin subunit 1 | 1.39 |
| LOC106607684 | XM_014204888.1 | N-alpha-acetyltransferase 20-like | 1.39 |
| md19b | NM_001146654.1 | Mediator of RNA polymerase II transcription subunit 19-B | 1.39 |
| LOC106568761 | XM_014139399.1 | UMP-CMP kinase | 1.39 |
| mfap1 | XM_014127584.1 | microfibrillar-associated protein 1 | 1.39 |
| prpf18 | XM_014124963.1 | pre-mRNA processing factor 18 | 1.39 |
| LOC106578297 | XM_014156968.1 | V-type proton ATPase 16 kDa proteolipid subunit | 1.39 |
| LOC100136564 | NM_001123657.1 | ferritin heavy subunit | 1.39 |
| mea1 | XM_014154081.1 | male-enhanced antigen 1 | 1.39 |
| LOC106587425 | XM_014175803.1 | probable ribosome biogenesis protein RLP24 | 1.39 |
| gbrap | NM_001142717.1 | Gamma-aminobutyric acid receptor-associated protein | 1.39 |
| chac1 | NM_001140335.1 | ChaC%2C cation transport regulator-like 1 | 1.39 |
| meaf6 | XM_014142533.1 | MYST/Esa1-associated factor 6 | 1.39 |
| rbx1 | NM_001123564.1 | ring-box 1 | 1.39 |
| tma20 | NM_001141426.1 | Translation machinery-associated protein 20 | 1.39 |
| LOC106579473 | XM_014159414.1 | eukaryotic translation initiation factor 1A%2C X-chromosomal | 1.39 |
| gtpba | XM_014202036.1 | GTP-binding protein 10 | 1.39 |
| LOC106568863 | XM_014139604.1 | protein FAM131A-like | 1.39 |
| twf1 | NM_001140132.1 | Twinfilin-1 | 1.39 |
| LOC106590604 | XM_014181724.1 | proteasome maturation protein-like | 1.39 |
| LOC106566794 | XM_014135189.1 | transforming protein RhoA-like | 1.38 |
| LOC106610772 | XM_014210331.1 | cytochrome c oxidase subunit 7A2%2C mitochondrial-like | 1.38 |
| LOC106599031 | XM_014190084.1 | ubiquitin-60S ribosomal protein L40-like | 1.38 |
| LOC106602047 | XM_014194496.1 | ATP synthase subunit d%2C mitochondrial-like | 1.38 |
| LOC106588193 | XM_014176940.1 | ATPase inhibitor A%2C mitochondrial-like | 1.38 |
| sap18 | NM_001279001.1 | Histone deacetylase complex subunit SAP18 | 1.38 |
| LOC106567406 | XM_014136601.1 | complement component 1 Q subcomponent-binding protein%2C mitochondrial-like | 1.38 |
| eif4e2 | XM_014192045.1 | eukaryotic translation initiation factor 4E family member 2 | 1.38 |
| LOC106611512 | XM_014211791.1 | ubiquitin-conjugating enzyme E2 A | 1.38 |
| LOC106612297 | XM_014213339.1 | glutathione peroxidase 3-like | 1.38 |
| LOC106562065 | XM_014126614.1 | embryonic polyadenylate-binding protein 2-B-like | 1.38 |
| LOC106565035 | XM_014131620.1 | cytochrome c oxidase subunit 6C-1 | 1.38 |
| LOC106567767 | XM_014137483.1 | non-POU domain-containing octamer-binding protein-like | 1.38 |
| LOC106611785 | XM_014212367.1 | small integral membrane protein 19-like | 1.38 |
| fkb1b | NM_001141565.2 | FK506-binding protein 1B | 1.38 |
| inip | XM_014147377.1 | INTS3 and NABP interacting protein | 1.38 |
| LOC106606979 | XM_014203510.1 | THO complex subunit 4-like | 1.38 |
| LOC106562230 | XM_014126959.1 | cleavage and polyadenylation specificity factor subunit 5-like | 1.38 |
| acbd7 | NM_001141117.1 | acyl-CoA binding domain containing 7 | 1.38 |
| atp5h | NM_001139687.1 | ATP synthase%2C H+ transporting%2C mitochondrial F0 complex%2C subunit d | 1.38 |
| commd10 | NM_001146382.1 | COMM domain containing 10 | 1.38 |
| LOC106598624 | XM_014189722.1 | galectin-related protein B-like | 1.38 |
| LOC106606918 | XM_014203387.1 | ER membrane protein complex subunit 10-like | 1.38 |
| rwdd4 | XM_014206481.1 | RWD domain containing 4 | 1.38 |
| LOC106593029 | XM_014184373.1 | cyclin-dependent kinase 5 activator 2-like | 1.38 |
| LOC106566500 | XM_014134587.1 | ER membrane protein complex subunit 3-like | 1.38 |
| ift22 | NM_001140751.1 | intraflagellar transport 22 | 1.38 |
| fkbp3 | NM_001141274.1 | FK506 binding protein 3 | 1.38 |
| sae1 | NM_001146514.1 | SUMO1 activating enzyme subunit 1 | 1.38 |
| ppia | NM_001146606.1 | peptidylprolyl isomerase A (cyclophilin A) | 1.38 |
| LOC106585258 | XM_014171268.1 | eukaryotic translation initiation factor 4E-binding protein 2-like | 1.38 |
| LOC106574824 | XM_014150915.1 | PEST proteolytic signal-containing nuclear protein-like | 1.38 |
| LOC106567425 | XM_014136661.1 | tetratricopeptide repeat protein 1-like | 1.38 |
| LOC106577413 | XM_014155380.1 | fumarate hydratase%2C mitochondrial | 1.38 |
| LOC106563496 | XM_014129146.1 | peptidyl-prolyl cis-trans isomerase FKBP2-like | 1.38 |
| LOC106563497 | XM_014129147.1 | protein phosphatase 1 regulatory subunit 14B-like | 1.38 |
| LOC106562224 | XM_014126951.1 | probable ribosome biogenesis protein RLP24 | 1.38 |
| ddrgk1 | XM_014132927.1 | DDRGK domain containing 1 | 1.38 |
| LOC106576246 | XM_014153314.1 | mitochondrial inner membrane protease ATP23 homolog | 1.38 |
| atp5g3 | XM_014172926.1 | ATP synthase%2C H+ transporting%2C mitochondrial F0 complex%2C subunit c-3 | 1.38 |
| LOC106562614 | XM_014127569.1 | mortality factor 4-like protein 1 | 1.38 |
| LOC106578266 | XM_014156930.1 | SUMO-conjugating enzyme UBC9-B-like | 1.38 |
| grl1 | NM_001140623.1 | Gamma-aminobutyric acid receptor-associated protein-like 1 | 1.37 |
| LOC106587585 | XM_014176115.1 | 39S ribosomal protein L4%2C mitochondrial-like | 1.37 |
| LOC106610898 | XM_014210565.1 | pre-mRNA-splicing factor syf2-like | 1.37 |
| gabarapl2 | XM_014148919.1 | GABA(A) receptor-associated protein like 2 | 1.37 |
| LOC106610623 | XM_014210040.1 | uncharacterized protein C15orf57 homolog | 1.37 |
| nudc | NM_001146614.2 | nuclear distribution gene C homolog | 1.37 |
| LOC106613943 | XM_014216789.1 | dolichyl-diphosphooligosaccharide--protein glycosyltransferase subunit DAD1-like | 1.37 |
| LOC106607599 | XM_014204688.1 | proteasome subunit alpha type-6 | 1.37 |
| LOC106581268 | XM_014163224.1 | 28S ribosomal protein S23%2C mitochondrial-like | 1.37 |
| LOC106584850 | XM_014170455.1 | dnaJ homolog subfamily A member 4-like | 1.37 |
| LOC106608115 | XM_014205835.1 | splicing factor 3B subunit 6 | 1.37 |
| commd7 | NM_001141365.1 | COMM domain containing 7 | 1.37 |
| LOC106563505 | XM_014129157.1 | cofilin-2-like | 1.37 |
| anp32b | XM_014209536.1 | acidic (leucine-rich) nuclear phosphoprotein 32 family%2C member B | 1.37 |
| s7a6o | XM_014126558.1 | SLC7A6OS | 1.37 |
| LOC106603799 | XM_014197914.1 | small nuclear ribonucleoprotein Sm D2-like | 1.37 |
| LOC106608669 | XM_014206761.1 | G protein pathway suppressor 2-like | 1.37 |
| LOC106563029 | XM_014128235.1 | V-type proton ATPase subunit G 1-like | 1.37 |
| LOC106606648 | XM_014202992.1 | protein YIPF4 | 1.37 |
| mo4l1 | NM_001146521.1 | Mortality factor 4-like protein 1 | 1.37 |
| stmn4 | XM_014192519.1 | stathmin-like 4 | 1.37 |
| mrpl16 | XM_014151177.1 | mitochondrial ribosomal protein L16 | 1.37 |
| LOC106566962 | XM_014135660.1 | transcription initiation factor TFIID subunit 10-like | 1.37 |
| lerl1 | XM_014199416.1 | Leptin receptor overlapping transcript-like 1 | 1.37 |
| sar1a | NM_001141789.1 | SAR1 gene homolog A (S. cerevisiae) | 1.37 |
| LOC106595829 | XM_014187174.1 | uncharacterized protein KIAA1143 homolog | 1.36 |
| nduba | NM_001141557.1 | NADH dehydrogenase 1 beta subcomplex subunit 10 | 1.36 |
| LOC106572706 | XM_014147111.1 | N-alpha-acetyltransferase 10-like | 1.36 |
| LOC106588698 | XM_014177962.1 | mediator of RNA polymerase II transcription subunit 10 | 1.36 |
| LOC106573579 | XM_014148732.1 | phospholipid hydroperoxide glutathione peroxidase%2C mitochondrial-like | 1.36 |
| LOC106585845 | XM_014172529.1 | transmembrane emp24 domain-containing protein 7-like | 1.36 |
| kpra | XM_014131117.1 | Phosphoribosyl pyrophosphate synthetase-associated protein 1 | 1.36 |
| crip2 | NM_001146545.1 | cysteine-rich protein 2 | 1.36 |
| LOC106563671 | XM_014129454.1 | high mobility group protein B3-like | 1.36 |
| LOC106613902 | XM_014216652.1 | calreticulin-like | 1.36 |
| LOC106592861 | XM_014184209.1 | deoxyhypusine synthase-like | 1.36 |
| djc18 | NM_001165308.1 | DnaJ homolog subfamily C member 18 | 1.36 |
| ub2v1 | NM_001140875.1 | Ubiquitin-conjugating enzyme E2 variant 1 | 1.36 |
| LOC106609785 | XM_014208797.1 | nuclear-interacting partner of ALK-like | 1.36 |
| arpc3 | NM_001142718.1 | actin related protein 2/3 complex%2C subunit 3 | 1.36 |
| LOC106609717 | XM_014208689.1 | DNA-(apurinic or apyrimidinic site) lyase-like | 1.36 |
| snf8 | XM_014179044.1 | SNF8%2C ESCRT-II complex subunit | 1.36 |
| LOC106601210 | XM_014193231.1 | heme-binding protein 1-like | 1.36 |
| LOC106582216 | XM_014165071.1 | BTB/POZ domain-containing protein KCTD4-like | 1.36 |
| LOC106571095 | XM_014143764.1 | ER membrane protein complex subunit 7-like | 1.36 |
| LOC106566721 | XM_014135038.1 | prostaglandin E synthase 3-like | 1.36 |
| LOC106603926 | XM_014198178.1 | heterogeneous nuclear ribonucleoprotein A/B-like | 1.36 |
| LOC106588845 | XM_014178326.1 | chromobox protein homolog 3-like | 1.36 |
| LOC106592831 | XM_014184177.1 | COX assembly mitochondrial protein homolog | 1.36 |
| commd9 | NM_001141001.1 | COMM domain containing 9 | 1.36 |
| LOC106560679 | XM_014123786.1 | ATP synthase subunit g%2C mitochondrial-like | 1.36 |
| wdr70 | XM_014130171.1 | WD repeat domain 70 | 1.36 |
| pop7 | XM_014206985.1 | POP7 homolog%2C ribonuclease P/MRP subunit | 1.36 |
| LOC106568019 | XM_014138012.1 | cytochrome c oxidase subunit 7B%2C mitochondrial-like | 1.36 |
| LOC106601696 | XM_014194049.1 | protein Tob1-like | 1.36 |
| LOC106589015 | XM_014178644.1 | chromatin modification-related protein MEAF6 | 1.36 |
| LOC106608215 | XM_014206046.1 | V-type proton ATPase subunit D-like | 1.36 |
| LOC106589849 | XM_014180249.1 | serine/arginine-rich splicing factor 2-like | 1.36 |
| LOC106571313 | XM_014144237.1 | ribosome production factor 2 homolog | 1.36 |
| LOC106581011 | XM_014162664.1 | methylosome subunit pICln-like | 1.36 |
| LOC100196400 | XM_014137639.1 | protein CWC15 homolog | 1.36 |
| LOC106611761 | XM_014212307.1 | cofilin-2-like | 1.35 |
| LOC106599769 | XM_014191080.1 | serine/arginine-rich splicing factor 11-like | 1.35 |
| LOC106609405 | XM_014208200.1 | protein LLP homolog | 1.35 |
| fam133b | XM_014142764.1 | family with sequence similarity 133%2C member B | 1.35 |
| LOC106605847 | XM_014201823.1 | splicing factor U2AF 65 kDa subunit-like | 1.35 |
| fam69a | XM_014123115.1 | family with sequence similarity 69%2C member A | 1.35 |
| LOC106567218 | XM_014136245.1 | tumor protein D54-like | 1.35 |
| cops5 | XM_014181414.1 | COP9 signalosome subunit 5 | 1.35 |
| LOC106570102 | XM_014142107.1 | uncharacterized LOC106570102 | 1.35 |
| cc068 | NM_001140764.1 | CC068 protein | 1.35 |
| LOC106560853 | XM_014124227.1 | protein FAM192A-like | 1.35 |
| LOC106570228 | XM_014142325.1 | thioredoxin-like protein 4A | 1.35 |
| sels | NM_001140931.1 | selenoprotein S | 1.35 |
| LOC106562058 | XM_014126601.1 | mitotic spindle-associated MMXD complex subunit MIP18-like | 1.35 |
| id2 | XM_014210193.1 | inhibitor of DNA binding 2%2C dominant negative helix-loop-helix protein | 1.35 |
| LOC106602900 | XM_014195884.1 | gamma-aminobutyric acid receptor-associated protein | 1.35 |
| LOC106604794 | XM_014199838.1 | protocadherin alpha-C2-like | 1.35 |
| emc4 | NM_001141096.1 | ER membrane protein complex subunit 4 | 1.35 |
| LOC106567526 | XM_014136881.1 | S-phase kinase-associated protein 1 | 1.35 |
| LOC106586704 | XM_014174252.1 | protein jagunal homolog 1-A-like | 1.35 |
| LOC106580350 | XM_014161293.1 | selenoprotein M-like | 1.35 |
| LOC106563108 | XM_014128333.1 | corticotropin-releasing factor-binding protein-like | 1.35 |
| LOC106584215 | XM_014169252.1 | proteasome subunit beta type-5-like | 1.35 |
| LOC106607492 | XM_014204489.1 | ATP synthase F(0) complex subunit C3%2C mitochondrial-like | 1.35 |
| bok | XM_014189908.1 | BCL2-related ovarian killer | 1.35 |
| LOC106591015 | XM_014182185.1 | E3 ubiquitin-protein ligase RNF115-like | 1.35 |
| hexim | NM_001139959.1 | HEXIM protein | 1.35 |
| denr | NM_001141283.1 | density-regulated protein | 1.35 |
| LOC106607142 | XM_014203767.1 | DNA-directed RNA polymerases I%2C II%2C and III subunit RPABC2-like | 1.34 |
| LOC106564007 | XM_014129967.1 | gastrula zinc finger protein XlCGF57.1-like | 1.34 |
| LOC106570207 | XM_014142291.1 | uncharacterized LOC106570207 | 1.34 |
| LOC106586318 | XM_014173487.1 | methylmalonic aciduria and homocystinuria type D protein%2C mitochondrial-like | 1.34 |
| psmd9 | NM_001141185.1 | proteasome (prosome%2C macropain) 26S subunit%2C non-ATPase%2C 9 | 1.34 |
| cssa10h1orf52 | NM_001141204.1 | chromosome ssa10 open reading frame%2C human C1orf52 | 1.34 |
| LOC106576269 | XM_014153352.1 | V-type proton ATPase subunit E 1-like | 1.34 |
| LOC106594500 | XM_014185871.1 | anaphase-promoting complex subunit 16 | 1.34 |
| LOC106587420 | XM_014175797.1 | cleavage and polyadenylation specificity factor subunit 5-like | 1.34 |
| 7b2 | XM_014143772.1 | Neuroendocrine protein 7B2 | 1.34 |
| calm2 | NM_001139713.1 | calmodulin 2 (phosphorylase kinase%2C delta) | 1.34 |
| LOC106611558 | XM_014211902.1 | peptidyl-prolyl cis-trans isomerase D-like | 1.34 |
| LOC106602970 | XM_014196045.1 | Sjoegren syndrome/scleroderma autoantigen 1 homolog | 1.34 |
| LOC106565058 | XM_014131679.1 | eukaryotic translation initiation factor 2 subunit 2-like | 1.34 |
| LOC106573595 | XM_014148772.1 | yjeF N-terminal domain-containing protein 3-like | 1.34 |
| LOC106562286 | XM_014127068.1 | cytochrome c oxidase subunit 4 isoform 1%2C mitochondrial-like | 1.34 |
| LOC106569426 | XM_014140774.1 | proteasome subunit beta type-5-like | 1.34 |
| sfrs9 | XM_014128542.1 | Splicing factor%2C arginine/serine-rich 9 | 1.34 |
| LOC106563585 | XM_014129301.1 | eukaryotic translation initiation factor 1A%2C X-chromosomal-like | 1.34 |
| LOC106589310 | XM_014179106.1 | proteasome activator complex subunit 3-like | 1.34 |
| LOC106598876 | XM_014189895.1 | ras-related protein Rab-6B-like | 1.34 |
| LOC106601335 | XM_014193466.1 | solute carrier family 25 member 38-B-like | 1.34 |
| slc25a11 | XM_014156891.1 | solute carrier family 25 (mitochondrial carrier%3B oxoglutarate carrier)%2C member 11 | 1.34 |
| arpc5l | NM_001141375.1 | actin related protein 2/3 complex%2C subunit 5-like | 1.34 |
| cssa11h9orf16 | XM_014128744.1 | chromosome ssa11 open reading frame%2C human C9orf16 | 1.34 |
| LOC106609347 | XM_014208082.1 | nuclear transcription factor Y subunit beta-like | 1.34 |
| psmc1 | XM_014211088.1 | proteasome 26S subunit%2C ATPase 1 | 1.34 |
| LOC106586159 | XM_014173071.1 | mitochondrial import receptor subunit TOM20 homolog | 1.33 |
| LOC106576424 | XM_014153602.1 | myotrophin | 1.33 |
| LOC106567919 | XM_014137811.1 | Purkinje cell protein 4-like | 1.33 |
| chga | XM_014145052.1 | chromogranin A | 1.33 |
| LOC106576510 | XM_014153727.1 | prickle-like protein 1 | 1.33 |
| LOC106580927 | XM_014162485.1 | succinate dehydrogenase [ubiquinone] cytochrome b small subunit B%2C mitochondrial-like | 1.33 |
| LOC106587003 | XM_014174788.1 | surfeit locus protein 4-like | 1.33 |
| LOC106589470 | XM_014179498.1 | WW domain-binding protein 2-like | 1.33 |
| cf105 | NM_001141548.1 | CF105 protein | 1.33 |
| LOC106593509 | XM_014184850.1 | DOMON domain-containing protein FRRS1L-like | 1.33 |
| LOC106574862 | XM_014150979.1 | synapse-associated protein 1-like | 1.33 |
| chchd6 | XM_014132081.1 | coiled-coil-helix-coiled-coil-helix domain containing 6 | 1.33 |
| aarsd1 | NM_001140214.1 | alanyl-tRNA synthetase domain containing 1 | 1.33 |
| LOC106575808 | XM_014152500.1 | retinoic acid-induced protein 2-like | 1.33 |
| cbx1 | NM_001165285.1 | chromobox homolog 1 | 1.33 |
| LOC106613741 | XM_014216308.1 | cold-inducible RNA-binding protein B-like | 1.33 |
| LOC106571589 | XM_014144872.1 | uncharacterized LOC106571589 | 1.33 |
| lsm7 | XM_014141126.1 | LSM7 homolog%2C U6 small nuclear RNA and mRNA degradation associated | 1.33 |
| taf11 | NM_001141392.1 | TAF11 RNA polymerase II%2C TATA box binding protein (TBP)-associated factor%2C 28kDa | 1.33 |
| LOC106576832 | XM_014154285.1 | gamma-aminobutyric acid receptor-associated protein-like 1 | 1.33 |
| LOC106599148 | XM_014190253.1 | calreticulin-like | 1.33 |
| ube2g2 | XM_014165761.1 | ubiquitin-conjugating enzyme E2G 2 | 1.33 |
| LOC106562977 | XM_014128176.1 | heterogeneous nuclear ribonucleoprotein R | 1.33 |
| LOC106587125 | XM_014175169.1 | hematological and neurological expressed 1 protein-like | 1.33 |
| LOC106592674 | XM_014184011.1 | zinc finger protein 330-like | 1.33 |
| prdx1 | XM_014179684.1 | peroxiredoxin 1 | 1.33 |
| LOC106561718 | XM_014125933.1 | uncharacterized LOC106561718 | 1.33 |
| neum | NM_001139802.1 | Neuromodulin | 1.33 |
| ndka | NM_001141244.1 | Nucleoside diphosphate kinase A | 1.33 |
| LOC106572179 | XM_014146100.1 | stathmin-3-like | 1.33 |
| LOC106609141 | XM_014207620.1 | V-type proton ATPase subunit F | 1.33 |
| lzic | XM_014145630.1 | leucine zipper and CTNNBIP1 domain containing | 1.33 |
| LOC106600385 | XM_014191691.1 | peroxisomal biogenesis factor 19-like | 1.33 |
| ub2l3 | XM_014138439.1 | Ubiquitin-conjugating enzyme E2 L3 | 1.33 |
| chst2 | NM_001140092.1 | Carbohydrate sulfotransferase 2 | 1.33 |
| LOC106610994 | XM_014210774.1 | charged multivesicular body protein 3 | 1.32 |
| LOC106567263 | XM_014136363.1 | cell division control protein 42 homolog | 1.32 |
| tsr3 | XM_014158932.1 | TSR3%2C 20S rRNA accumulation%2C homolog (S. cerevisiae) | 1.32 |
| ndufa3 | NM_001141206.1 | NADH dehydrogenase (ubiquinone) 1 alpha subcomplex%2C 3%2C 9kDa | 1.32 |
| LOC106568888 | XM_014139671.1 | calreticulin-like | 1.32 |
| yjefn3 | XM_014216300.1 | YjeF N-terminal domain containing 3 | 1.32 |
| LOC106604708 | XM_014199640.1 | cofilin-2-like | 1.32 |
| ppia1 | NM_001141689.1 | 2-peptidylprolyl isomerase A | 1.32 |
| LOC106565026 | XM_014131599.1 | dynein light chain roadblock-type 1 | 1.32 |
| uba3 | XM_014166581.1 | ubiquitin-like modifier activating enzyme 3 | 1.32 |
| LOC106565555 | XM_014132813.1 | actin-related protein 2/3 complex subunit 4 | 1.32 |
| cfdp1 | XM_014149074.1 | craniofacial development protein 1 | 1.32 |
| LOC106581067 | XM_014162776.1 | ribosome maturation protein SBDS | 1.32 |
| LOC106563281 | XM_014128746.1 | surfeit locus protein 4 | 1.32 |
| ub2g1 | XM_014133923.1 | Ubiquitin-conjugating enzyme E2 G1 | 1.32 |
| stml2 | NM_001141736.1 | Stomatin-like protein 2 | 1.32 |
| LOC106601585 | XM_014193896.1 | transcription elongation factor B polypeptide 2 | 1.32 |
| LOC106571686 | XM_014145049.1 | activator of 90 kDa heat shock protein ATPase homolog 1-like | 1.32 |
| dcps | XM_014197827.1 | decapping enzyme%2C scavenger | 1.32 |
| LOC106570531 | XM_014142960.1 | transformer-2 protein homolog alpha-like | 1.32 |
| tbca | NM_001140987.1 | tubulin folding cofactor A | 1.32 |
| LOC106567531 | XM_014136892.1 | GTP-binding protein SAR1a-like | 1.32 |
| LOC106573700 | XM_014148990.1 | heat shock factor-binding protein 1-like | 1.32 |
| u2af2 | XM_014154451.1 | U2 small nuclear RNA auxiliary factor 2 | 1.32 |
| LOC106601215 | XM_014193236.1 | coiled-coil domain-containing protein 134-like | 1.32 |
| LOC106576015 | XM_014152847.1 | adenylyltransferase and sulfurtransferase MOCS3-like | 1.32 |
| tp4a1 | NM_001165365.1 | tyrosine phosphatase type IVA 1 | 1.32 |
| LOC100136498 | NM_001123609.1 | macrophage migration inhibitory factor | 1.32 |
| LOC106601546 | XM_014193839.1 | uncharacterized LOC106601546 | 1.32 |
| tppc3 | XM_014178210.1 | Trafficking protein particle complex subunit 3 | 1.32 |
| LOC106585458 | XM_014171683.1 | SWI/SNF-related matrix-associated actin-dependent regulator of chromatin subfamily B member 1 | 1.32 |
| LOC106593576 | XM_014184926.1 | conserved oligomeric Golgi complex subunit 1-like | 1.32 |
| fbxw12 | XM_014145436.1 | F-box and WD repeat domain containing 12 | 1.32 |
| LOC106589537 | XM_014179641.1 | 28 kDa heat- and acid-stable phosphoprotein-like | 1.32 |
| cf166 | NM_001165297.1 | CF166 protein | 1.31 |
| LOC106567495 | XM_014136789.1 | NEDD4 family-interacting protein 1-like | 1.31 |
| LOC106595987 | XM_014187323.1 | V-type proton ATPase subunit H-like | 1.31 |
| rab35 | XM_014160050.1 | RAB35%2C member RAS oncogene family | 1.31 |
| LOC106613487 | XM_014215769.1 | profilin-2-like | 1.31 |
| LOC106593403 | XM_014184737.1 | contactin-1-like | 1.31 |
| LOC106609277 | XM_014207904.1 | cell division cycle protein 123 homolog | 1.31 |
| LOC106576032 | XM_014152852.1 | ragulator complex protein LAMTOR4-like | 1.31 |
| LOC106591949 | XM_014183220.1 | guanine nucleotide-binding protein G(I)/G(S)/G(O) subunit gamma-13-like | 1.31 |
| LOC106564619 | XM_014130785.1 | UPF0585 protein C16orf13 homolog A-like | 1.31 |
| LOC106571096 | XM_014143765.1 | KATNB1-like protein 1 | 1.31 |
| ube2b | NM_001146361.1 | ubiquitin-conjugating enzyme E2B (RAD6 homolog) | 1.31 |
| atp6v0c | XM_014192208.1 | ATPase%2C H+ transporting%2C lysosomal 16kDa%2C V0 subunit c | 1.31 |
| mk67i | NM_001140700.1 | MKI67 FHA domain-interacting nucleolar phosphoprotein-like | 1.31 |
| acp1 | XM_014205655.1 | acid phosphatase 1%2C soluble | 1.31 |
| surf1 | NM_001141597.1 | surfeit 1 | 1.31 |
| LOC106573472 | XM_014148558.1 | casein kinase II subunit beta-like | 1.31 |
| LOC106576943 | XM_014154490.1 | splicing factor U2AF 65 kDa subunit-like | 1.31 |
| LOC106573420 | XM_014148450.1 | CD9 antigen-like | 1.31 |
| LOC106589821 | XM_014180198.1 | mitochondrial pyruvate carrier 1-like | 1.31 |
| LOC106577817 | XM_014156173.1 | thioredoxin-like | 1.31 |
| pebp1 | NM_001141612.1 | phosphatidylethanolamine binding protein 1 | 1.31 |
| commd3 | NM_001141012.1 | COMM domain containing 3 | 1.31 |
| LOC106580669 | XM_014161976.1 | eukaryotic translation initiation factor 4H-like | 1.31 |
| ube2v1 | NM_001205110.1 | ubiquitin-conjugating enzyme E2 variant 1 | 1.31 |
| LOC106577557 | XM_014155700.1 | gamma-aminobutyric acid receptor-associated protein-like | 1.31 |
| LOC106585280 | XM_014171334.1 | arginine/serine-rich coiled-coil protein 2-like | 1.31 |
| LOC106612293 | XM_014213328.1 | serine/arginine-rich splicing factor 2-like | 1.31 |
| enp6 | NM_001141709.1 | Ectonucleoside triphosphate diphosphohydrolase 6 | 1.31 |
| LOC106569985 | XM_014141796.1 | triosephosphate isomerase A | 1.31 |
| LOC100196748 | XM_014150142.1 | quinone oxidoreductase-like protein 1 | 1.31 |
| mtpn | NM_001165386.1 | myotrophin | 1.31 |
| ndufc1 | XM_014212340.1 | NADH dehydrogenase (ubiquinone) 1%2C subcomplex unknown%2C 1%2C 6kDa | 1.30 |
| LOC106580784 | XM_014162192.1 | 14-3-3 protein epsilon-like | 1.30 |
| fam50a | NM_001146427.1 | family with sequence similarity 50%2C member A | 1.30 |
| LOC106586370 | XM_014173569.1 | MOB-like protein phocein | 1.30 |
| LOC106590414 | XM_014181416.1 | ER membrane protein complex subunit 9-like | 1.30 |
| LOC106586850 | XM_014174569.1 | ice-structuring glycoprotein-like | 1.30 |
| LOC106582215 | XM_014165068.1 | translationally-controlled tumor protein homolog | 1.30 |
| LOC106610722 | XM_014210239.1 | 14-3-3 protein beta/alpha-1-like | 1.30 |
| LOC106567415 | XM_014136622.1 | heterogeneous nuclear ribonucleoprotein A/B-like | 1.30 |
| LOC106609902 | XM_014208950.1 | NADH dehydrogenase [ubiquinone] iron-sulfur protein 8%2C mitochondrial-like | 1.30 |
| polr2c | NM_001141873.1 | polymerase (RNA) II (DNA directed) polypeptide C | 1.30 |
| LOC106604403 | XM_014198988.1 | ubiquitin-conjugating enzyme E2 D2 | 1.30 |
| dap1 | NM_001141583.1 | Death-associated protein 1 | 1.30 |
| LOC106564857 | XM_014131322.1 | malate dehydrogenase%2C cytoplasmic-like | 1.30 |
| fkbp1b | XM_014205706.1 | FK506 binding protein 1B%2C 12.6 kDa | 1.30 |
| drg1 | XM_014131454.1 | developmentally regulated GTP binding protein 1 | 1.30 |
| LOC106575588 | XM_014152186.1 | histone deacetylase complex subunit SAP18-like | 1.30 |
| LOC106584314 | XM_014169422.1 | acyl-CoA-binding domain-containing protein 6-like | 1.30 |
| LOC106611998 | XM_014212752.1 | E3 ubiquitin-protein ligase RNF4-like | 1.30 |
| ub2e3 | XM_014165028.1 | Ubiquitin-conjugating enzyme E2 E3 | 1.30 |
| sox2 | NM_001141718.1 | SRY-box containing gene 2 | 1.30 |
| LOC106583167 | XM_014167033.1 | class E basic helix-loop-helix protein 40-like | 1.30 |
| LOC106563719 | XM_014129530.1 | gamma-aminobutyric acid receptor subunit alpha-5-like | 1.30 |
| LOC106585454 | XM_014171675.1 | protein SET-like | 1.30 |
| LOC106563500 | XM_014129151.1 | calmodulin | 1.30 |
| saraf | XM_014209177.1 | store-operated calcium entry-associated regulatory factor | 1.30 |
| ppme1 | XM_014137585.1 | protein phosphatase methylesterase 1 | 1.30 |
| LOC106572212 | XM_014146190.1 | transcription initiation factor TFIID subunit 10-like | 1.30 |
| LOC106578494 | XM_014157352.1 | cytochrome c-type heme lyase-like | 1.30 |
| LOC106567379 | XM_014136550.1 | phosphatase and actin regulator 3-like | 1.29 |
| LOC106579942 | XM_014160366.1 | cytochrome c oxidase subunit 6A%2C mitochondrial | 1.29 |
| ube2i | XM_014158940.1 | ubiquitin-conjugating enzyme E2I | 1.29 |
| pesc | NM_001139866.1 | Pescadillo | 1.29 |
| naa38 | XM_014155956.1 | N(alpha)-acetyltransferase 38%2C NatC auxiliary subunit | 1.29 |
| LOC106574719 | XM_014150735.1 | paraspeckle component 1-like | 1.29 |
| mrps34 | XM_014192203.1 | mitochondrial ribosomal protein S34 | 1.29 |
| LOC106603869 | XM_014198073.1 | neuron-specific protein family member 2-like | 1.29 |
| LOC106562632 | XM_014127597.1 | transmembrane emp24 domain-containing protein 2-like | 1.29 |
| LOC106593686 | XM_014185058.1 | guanine nucleotide-binding protein G(I)/G(S)/G(O) subunit gamma-13-like | 1.29 |
| LOC106563531 | XM_014129212.1 | protein TSSC4-like | 1.29 |
| LOC106580428 | XM_014161484.1 | dynein light chain 1%2C cytoplasmic | 1.29 |
| hc127 | XM_014198747.1 | Hepatocellular carcinoma-associated antigen 127 | 1.29 |
| LOC106582071 | XM_014164790.1 | ras-related protein Rab-5A-like | 1.29 |
| LOC106598823 | XM_014189830.1 | DCN1-like protein 1 | 1.29 |
| LOC106562540 | XM_014127480.1 | myelin expression factor 2-like | 1.29 |
| LOC106574700 | XM_014150711.1 | cyclin-dependent kinase 5 activator 1-like | 1.29 |
| ik | NM_001173724.1 | IK cytokine%2C down-regulator of HLA II | 1.29 |
| zn706 | NM_001141044.1 | Zinc finger protein 706 | 1.29 |
| LOC106570595 | XM_014143052.1 | protein SET-like | 1.29 |
| LOC106579304 | XM_014159111.1 | charged multivesicular body protein 2a-like | 1.29 |
| LOC106612133 | XM_014213037.1 | heterogeneous nuclear ribonucleoprotein H-like | 1.29 |
| LOC106583084 | XM_014166884.1 | serine/arginine-rich splicing factor 3 | 1.29 |
| LOC106577997 | XM_014156512.1 | heterogeneous nuclear ribonucleoprotein C-like | 1.29 |
| tmed2 | XM_014161100.1 | transmembrane emp24 domain trafficking protein 2 | 1.29 |
| cssa16h11orf49 | XM_014147803.1 | chromosome ssa16 open reading frame%2C human C11orf49 | 1.29 |
| LOC106606477 | XM_014202702.1 | core histone macro-H2A.2-like | 1.28 |
| LOC106589760 | XM_014180087.1 | mitoferrin-2-like | 1.28 |
| LOC106608224 | XM_014206056.1 | 26S protease regulatory subunit 4-like | 1.28 |
| LOC106576223 | XM_014153264.1 | nuclear transcription factor Y subunit beta-like | 1.28 |
| LOC106569287 | XM_014140478.1 | nudC domain-containing protein 3-like | 1.28 |
| LOC106607186 | XM_014203876.1 | SWI/SNF-related matrix-associated actin-dependent regulator of chromatin subfamily E member 1-like | 1.28 |
| tmm32 | NM_001141502.1 | Transmembrane protein 32 | 1.28 |
| lab | NM_001173766.1 | Lupus La protein homolog B | 1.28 |
| LOC106562047 | XM_014126588.1 | heterogeneous nuclear ribonucleoprotein K-like | 1.28 |
| LOC106593495 | XM_014184826.1 | A-kinase anchor protein 8-like | 1.28 |
| LOC106561397 | XM_014125339.1 | ornithine decarboxylase antizyme 2-like | 1.28 |
| LOC106560291 | XM_014123047.1 | growth hormone-inducible transmembrane protein-like | 1.28 |
| LOC106579971 | XM_014160440.1 | protein SET | 1.28 |
| spf30 | XM_014154464.1 | Survival of motor neuron-related-splicing factor 30 | 1.28 |
| LOC106588799 | XM_014178233.1 | mitochondrial import receptor subunit TOM7 homolog | 1.28 |
| stk25 | NM_001140433.1 | serine/threonine kinase 25 | 1.28 |
| LOC106578562 | XM_014157552.1 | syntaxin-12-like | 1.28 |
| elob | NM_001141486.2 | Transcription elongation factor B polypeptide 2 | 1.28 |
| npm | NM_001140148.1 | Nucleophosmin | 1.28 |
| LOC106607501 | XM_014204503.1 | eukaryotic translation initiation factor 1 | 1.28 |
| LOC106600883 | XM_014192633.1 | SUMO-conjugating enzyme UBC9-like | 1.28 |
| LOC106584447 | XM_014169800.1 | protein QIL1-like | 1.28 |
| LOC106567593 | XM_014137102.1 | histone H3.3 | 1.28 |
| LOC106564789 | XM_014131165.1 | apelin receptor B-like | 1.28 |
| LOC106600554 | XM_014191997.1 | glycine-rich RNA-binding protein-like | 1.28 |
| LOC106596773 | XM_014188036.1 | actin-related protein 2-A-like | 1.28 |
| ndufs3 | XM_014175094.1 | NADH dehydrogenase (ubiquinone) Fe-S protein 3%2C 30kDa (NADH-coenzyme Q reductase) | 1.28 |
| dyl2 | NM_001141605.1 | Dynein light chain 2%2C cytoplasmic | 1.28 |
| LOC106575630 | XM_014152240.1 | actin-related protein 2/3 complex subunit 2 | 1.28 |
| arf3 | XM_014135543.1 | ADP-ribosylation factor 3 | 1.27 |
| LOC106611763 | XM_014212312.1 | ubiquitin-like protein 3 | 1.27 |
| LOC106585115 | XM_014170960.1 | nucleolysin TIA-1 isoform p40-like | 1.27 |
| mtx2 | NM_001141391.2 | metaxin 2 | 1.27 |
| LOC106574227 | XM_014149918.1 | uncharacterized protein C7orf57 homolog | 1.27 |
| pdk1 | XM_014173278.1 | pyruvate dehydrogenase kinase%2C isozyme 1 | 1.27 |
| LOC106571089 | XM_014143757.1 | protein max-like | 1.27 |
| LOC106567090 | XM_014135974.1 | tubulin alpha chain | 1.27 |
| LOC106565439 | XM_014132580.1 | tubulin alpha chain-like | 1.27 |
| LOC106601115 | XM_014193052.1 | pinin-like | 1.27 |
| LOC106611940 | XM_014212628.1 | heterogeneous nuclear ribonucleoprotein A/B-like | 1.27 |
| LOC106581816 | XM_014164187.1 | uncharacterized protein C7orf57-like | 1.27 |
| LOC106612439 | XM_014213560.1 | high mobility group-T protein-like | 1.27 |
| LOC106571940 | XM_014145529.1 | prostaglandin E synthase 3-like | 1.27 |
| ci025 | XM_014160080.1 | CI025 protein | 1.27 |
| LOC106583126 | XM_014166959.1 | F-box only protein 2-like | 1.27 |
| kad | NM_001139707.1 | adenylate kinase 1-2 | 1.27 |
| LOC106612793 | XM_014214296.1 | dual specificity protein phosphatase 14-like | 1.27 |
| LOC100194716 | NM_001139761.1 | selenoprotein T-like | 1.27 |
| rabggtb | NM_001140171.1 | Rab geranylgeranyltransferase%2C beta subunit | 1.27 |
| LOC106561967 | XM_014126460.1 | mitochondrial carrier homolog 2-like | 1.27 |
| LOC106612606 | XM_014213924.1 | serine/arginine-rich splicing factor 7-like | 1.27 |
| tfpt | XM_014201661.1 | TCF3 (E2A) fusion partner (in childhood Leukemia) | 1.27 |
| LOC106603705 | XM_014197705.1 | histone H3.3 | 1.27 |
| chm1b | XM_014137435.1 | Charged multivesicular body protein 1b | 1.27 |
| LOC106567639 | XM_014137169.1 | zinc finger protein ZPR1-like | 1.27 |
| LOC106569367 | XM_014140676.1 | heterogeneous nuclear ribonucleoprotein C-like | 1.27 |
| LOC106576148 | XM_014153065.1 | mesoderm-specific transcript homolog protein-like | 1.27 |
| LOC106601841 | XM_014194245.1 | heme oxygenase 2-like | 1.27 |
| LOC106581187 | XM_014163083.1 | transcriptional adapter 2-alpha-like | 1.27 |
| ub2r1 | NM_001140115.1 | Ubiquitin-conjugating enzyme E2 R1 | 1.27 |
| cl012 | NM_001146475.1 | CL012 protein | 1.27 |
| taf7 | NM_001140248.1 | TAF7 RNA polymerase II%2C TATA box binding protein (TBP)-associated factor%2C 55kDa | 1.27 |
| LOC106607125 | XM_014203741.1 | WW domain-binding protein 2-like | 1.27 |
| LOC106580642 | XM_014161919.1 | high mobility group-T protein-like | 1.27 |
| LOC106564477 | XM_014130591.1 | programmed cell death protein 4-like | 1.27 |
| LOC106576681 | XM_014153984.1 | core histone macro-H2A.2-like | 1.27 |
| ppwd1 | XM_014138550.1 | peptidylprolyl isomerase domain and WD repeat containing 1 | 1.26 |
| emc6 | XM_014137940.1 | ER membrane protein complex subunit 6 | 1.26 |
| LOC100194664 | XM_014204698.1 | Vps20-associated 1 like 1 | 1.26 |
| LOC106584722 | XM_014170247.1 | COP9 signalosome complex subunit 2-like | 1.26 |
| LOC106601417 | XM_014193623.1 | cerebellar degeneration-related protein 2-like | 1.26 |
| LOC106613600 | XM_014216029.1 | small glutamine-rich tetratricopeptide repeat-containing protein alpha-like | 1.26 |
| LOC106566691 | XM_014134977.1 | vesicle-associated membrane protein-associated protein B-like | 1.26 |
| LOC106603160 | XM_014196468.1 | calmodulin | 1.26 |
| ube2j1 | NM_001139996.1 | ubiquitin-conjugating enzyme E2%2C J1 | 1.26 |
| pfn2 | XM_014149743.1 | profilin 2 | 1.26 |
| LOC106579642 | XM_014159747.1 | gamma-soluble NSF attachment protein-like | 1.26 |
| pelo | XM_014158567.1 | pelota homolog (Drosophila) | 1.26 |
| psd12 | NM_001141769.1 | 26S proteasome non-ATPase regulatory subunit 12 | 1.26 |
| ppp2r4 | NM_001141756.1 | protein phosphatase 2A activator%2C regulatory subunit 4 | 1.26 |
| LOC106592767 | XM_014184093.1 | acidic leucine-rich nuclear phosphoprotein 32 family member E-like | 1.26 |
| LOC100194554 | XM_014207488.1 | solute carrier family 25 member 3 | 1.26 |
| eef1e1 | XM_014181051.1 | eukaryotic translation elongation factor 1 epsilon 1 | 1.26 |
| rnf34 | XM_014130839.1 | ring finger protein 34%2C E3 ubiquitin protein ligase | 1.26 |
| LOC106582630 | XM_014165858.1 | cytochrome c1%2C heme protein%2C mitochondrial-like | 1.26 |
| LOC106585705 | XM_014172226.1 | 26S proteasome non-ATPase regulatory subunit 12-like | 1.26 |
| gmds | XM_014123207.1 | GDP-mannose 4%2C6-dehydratase | 1.25 |
| hnrpg | XM_014199418.1 | Heterogeneous nuclear ribonucleoprotein G | 1.25 |
| LOC106584440 | XM_014169779.1 | SWI/SNF-related matrix-associated actin-dependent regulator of chromatin subfamily E member 1-related-like | 1.25 |
| LOC106613829 | XM_014216480.1 | vesicle-trafficking protein SEC22b-B | 1.25 |
| LOC106584750 | XM_014170292.1 | transmembrane protein 178B-like | 1.25 |
| face1 | NM_001140076.1 | CAAX prenyl protease 1 homolog | 1.25 |
| LOC106575622 | XM_014152233.1 | FUN14 domain-containing protein 1-like | 1.25 |
| eif3j | XM_014175443.1 | eukaryotic translation initiation factor 3%2C subunit J | 1.25 |
| LOC106580918 | XM_014162474.1 | mitochondrial fission factor homolog B-like | 1.25 |
| oaz1s | NM_001142707.1 | ornithine decarboxylase antizyme 1S | 1.25 |
| LOC106571252 | XM_014144067.1 | 14-3-3 protein zeta | 1.25 |
| LOC106605558 | XM_014201344.1 | platelet-activating factor acetylhydrolase IB subunit gamma-like | 1.25 |
| LOC106603817 | XM_014197982.1 | voltage-dependent anion-selective channel protein 1 | 1.25 |
| hsf2 | XM_014204746.1 | heat shock factor 2 | 1.25 |
| LOC106579884 | XM_014160215.1 | transmembrane protein 127-like | 1.25 |
| LOC106584153 | XM_014169074.1 | ubiquitin carboxyl-terminal hydrolase isozyme L5-like | 1.25 |
| LOC106599679 | XM_014190972.1 | beta-soluble NSF attachment protein-like | 1.25 |
| LOC100194523 | NM_001139609.1 | MGC81610 protein-like | 1.25 |
| LOC106578074 | XM_014156653.1 | polyadenylate-binding protein-interacting protein 2B-like | 1.25 |
| LOC106576826 | XM_014154267.1 | nucleolysin TIAR-like | 1.25 |
| ap3s1 | XM_014160057.1 | adaptor-related protein complex 3%2C sigma 1 subunit | 1.24 |
| cacybp | NM_001140952.1 | calcyclin binding protein | 1.24 |
| LOC106576022 | XM_014152821.1 | serine-threonine kinase receptor-associated protein-like | 1.24 |
| LOC106610956 | XM_014210682.1 | eukaryotic translation initiation factor 5-like | 1.24 |
| renr | NM_001140091.1 | Renin receptor | 1.24 |
| LOC106572481 | XM_014146704.1 | ubiquitin-conjugating enzyme E2 variant 1 | 1.24 |
| LOC106571654 | XM_014144965.1 | cysteine-rich protein 2-like | 1.24 |
| LOC106612225 | XM_014213199.1 | NEDD4 family-interacting protein 1-like | 1.24 |
| LOC106567523 | XM_014136870.1 | voltage-dependent anion-selective channel protein 1-like | 1.24 |
| LOC106591329 | XM_014182544.1 | aspartate aminotransferase%2C cytoplasmic-like | 1.24 |
| LOC106593297 | XM_014184635.1 | ornithine aminotransferase%2C mitochondrial-like | 1.24 |
| LOC106590146 | XM_014180790.1 | myosin regulatory light polypeptide 9 | 1.24 |
| prpf3 | XM_014151195.1 | pre-mRNA processing factor 3 | 1.24 |
| LOC106612660 | XM_014214050.1 | 14-3-3 protein epsilon-like | 1.24 |
| LOC106603856 | XM_014198054.1 | NEDD4 family-interacting protein 1-like | 1.24 |
| idh3a | XM_014208669.1 | isocitrate dehydrogenase 3 (NAD+) alpha | 1.23 |
| LOC106600642 | XM_014192145.1 | phosphatidylinositol transfer protein beta isoform-like | 1.23 |
| LOC106583189 | XM_014167094.1 | ER membrane protein complex subunit 3 | 1.23 |
| LOC106586437 | XM_014173712.1 | basic leucine zipper and W2 domain-containing protein 1-A-like | 1.23 |
| hnrnpk | XM_014172158.1 | heterogeneous nuclear ribonucleoprotein K | 1.23 |
| LOC106583278 | XM_014167248.1 | tubulin alpha chain-like | 1.23 |
| rbtn1 | NM_001146398.1 | Rhombotin-1 | 1.23 |
| LOC106578933 | XM_014158211.1 | protein phosphatase 1B-like | 1.23 |
| LOC106609126 | XM_014207582.1 | serine-threonine kinase receptor-associated protein-like | 1.23 |
| LOC106564670 | XM_014130886.1 | small ubiquitin-related modifier 2-like | 1.23 |
| LOC100380720 | XM_014125643.1 | F-actin-capping protein subunit alpha-2 | 1.23 |
| LOC106574283 | XM_014150081.1 | acidic leucine-rich nuclear phosphoprotein 32 family member E-like | 1.23 |
| LOC106607800 | XM_014205163.1 | uncharacterized protein C1orf198 homolog | 1.23 |
| ap1m1 | XM_014148669.1 | adaptor-related protein complex 1%2C mu 1 subunit | 1.23 |
| LOC106610676 | XM_014210147.1 | protein phosphatase 1G-like | 1.22 |
| LOC106580853 | XM_014162336.1 | serine/arginine-rich splicing factor 7-like | 1.22 |
| ppp1cc | XM_014160404.1 | protein phosphatase 1%2C catalytic subunit%2C gamma isozyme | 1.22 |
| LOC106571634 | XM_014144936.1 | serine/threonine-protein phosphatase PP1-beta catalytic subunit-like | 1.22 |
| actr8 | NM_001140158.1 | ARP8 actin-related protein 8 homolog (yeast) | 1.22 |
| LOC106608292 | XM_014206155.1 | transmembrane emp24 domain-containing protein 10-like | 1.22 |
| arf1 | XM_014140595.1 | ADP-ribosylation factor 1 | 1.22 |
| LOC106605532 | XM_014201296.1 | protein LYRIC-like | 1.21 |
| cops3 | NM_001140561.1 | COP9 signalosome subunit 3 | 1.21 |
| LOC106606270 | XM_014202415.1 | UPF0472 protein C16orf72 homolog | 1.21 |
| LOC100194681 | XM_014200054.1 | heterogeneous nuclear ribonucleoprotein A/B | 1.21 |
| LOC100380682 | XM_014180902.1 | protein CDV3 homolog | 1.21 |
| LOC106566654 | XM_014134891.1 | 14-3-3 protein beta/alpha-2 | 1.20 |
| LOC106566965 | XM_014135663.1 | TAR DNA-binding protein 43-like | 1.20 |
| LOC106567579 | XM_014137062.1 | 14-3-3 protein epsilon-like | 1.20 |
| LOC106613688 | XM_014216216.1 | AP-1 complex subunit mu-1 | 1.19 |

**Table S5.** Gene Ontology (GO) categories with a significant (*p* < 0.05) overrepresentation of upregulated genes in Atlantic salmon juveniles subjected to 8 weeks of exercise, compared to unexercised control.

| GO ID | GO term | *p* value | No. genes in GO category | No. genes upregulated in GO category |
| --- | --- | --- | --- | --- |
| GO:0016059 | deactivation of rhodopsin mediated signaling | 4.91E-05 | 24 | 8 |
| GO:0018105 | peptidyl-serine phosphorylation | 0.000112069 | 332 | 28 |
| GO:0008340 | determination of adult lifespan | 0.000262183 | 356 | 29 |
| GO:0019722 | calcium-mediated signaling | 0.000280014 | 111 | 13 |
| GO:0016056 | rhodopsin mediated signaling pathway | 0.000389687 | 68 | 8 |
| GO:2000615 | regulation of histone H3-K9 acetylation | 0.000548256 | 38 | 9 |
| GO:0048477 | oogenesis | 0.000557648 | 187 | 18 |
| GO:0000185 | activation of MAPKKK activity | 0.000795386 | 32 | 6 |
| GO:0072577 | endothelial cell apoptotic process | 0.000807135 | 20 | 6 |
| GO:0051497 | negative regulation of stress fiber assembly | 0.000826275 | 88 | 12 |
| GO:0010470 | regulation of gastrulation | 0.000953507 | 45 | 10 |
| GO:0050803 | regulation of synapse structure or activity | 0.001047207 | 72 | 11 |
| GO:0043966 | histone H3 acetylation | 0.001257902 | 129 | 16 |
| GO:0042981 | regulation of apoptotic process | 0.00127063 | 245 | 21 |
| GO:0010717 | regulation of epithelial to mesenchymal transition | 0.001361786 | 47 | 9 |
| GO:0018107 | peptidyl-threonine phosphorylation | 0.001382254 | 177 | 18 |
| GO:0044257 | cellular protein catabolic process | 0.001394764 | 29 | 7 |
| GO:1903690 | negative regulation of wound healing, spreading of epidermal cells | 0.00143647 | 22 | 7 |
| GO:0043984 | histone H4-K16 acetylation | 0.001548036 | 62 | 11 |
| GO:0016458 | gene silencing | 0.001551823 | 44 | 10 |
| GO:0046488 | phosphatidylinositol metabolic process | 0.001586795 | 52 | 9 |
| GO:0090557 | establishment of endothelial intestinal barrier | 0.001887925 | 47 | 8 |
| GO:0031023 | microtubule organizing center organization | 0.002296447 | 27 | 7 |
| GO:0006987 | activation of signaling protein activity involved in unfolded protein response | 0.003059474 | 29 | 5 |
| GO:0080182 | histone H3-K4 trimethylation | 0.003100813 | 34 | 9 |
| GO:0007020 | microtubule nucleation | 0.003111667 | 47 | 8 |
| GO:0090091 | positive regulation of extracellular matrix disassembly | 0.003166918 | 29 | 7 |
| GO:0070983 | dendrite guidance | 0.003445746 | 30 | 9 |
| GO:0070972 | protein localization to endoplasmic reticulum | 0.00350467 | 21 | 7 |
| GO:0045927 | positive regulation of growth | 0.003686576 | 25 | 5 |
| GO:0061136 | regulation of proteasomal protein catabolic process | 0.003688617 | 95 | 11 |
| GO:0006903 | vesicle targeting | 0.003919159 | 41 | 7 |
| GO:0006974 | cellular response to DNA damage stimulus | 0.004441235 | 291 | 23 |
| GO:1901018 | positive regulation of potassium ion transmembrane transporter activity | 0.004441368 | 27 | 8 |
| GO:2000599 | NA | 0.004603574 | 22 | 5 |
| GO:2000021 | regulation of ion homeostasis | 0.00466305 | 24 | 7 |
| GO:2000651 | positive regulation of sodium ion transmembrane transporter activity | 0.005544155 | 44 | 11 |
| GO:0006338 | chromatin remodeling | 0.005792504 | 161 | 13 |
| GO:0043052 | thermotaxis | 0.00583157 | 32 | 6 |
| GO:0002385 | mucosal immune response | 0.006036644 | 21 | 5 |
| GO:0043547 | positive regulation of GTPase activity | 0.006186397 | 732 | 49 |
| GO:0051568 | histone H3-K4 methylation | 0.006212116 | 55 | 11 |
| GO:0044255 | cellular lipid metabolic process | 0.006426544 | 120 | 13 |
| GO:0034453 | microtubule anchoring | 0.006428213 | 31 | 7 |
| GO:0086014 | atrial cardiac muscle cell action potential | 0.006746725 | 28 | 7 |
| GO:0006306 | DNA methylation | 0.006752043 | 70 | 10 |
| GO:0006513 | protein monoubiquitination | 0.007015936 | 91 | 9 |
| GO:0007016 | cytoskeletal anchoring at plasma membrane | 0.00726445 | 68 | 13 |
| GO:0048589 | developmental growth | 0.007643379 | 84 | 10 |
| GO:0051482 | positive regulation of cytosolic calcium ion concentration involved in phospholipase C-activating G-protein coupled signaling pathway | 0.008130151 | 46 | 6 |
| GO:0051225 | spindle assembly | 0.008557846 | 78 | 9 |
| GO:2000648 | positive regulation of stem cell proliferation | 0.008945318 | 40 | 6 |
| GO:0019827 | stem cell population maintenance | 0.008984222 | 156 | 13 |
| GO:0043981 | histone H4-K5 acetylation | 0.009065338 | 48 | 7 |
| GO:0007279 | pole cell formation | 0.00928416 | 20 | 4 |
| GO:0043254 | regulation of protein complex assembly | 0.009417262 | 25 | 5 |
| GO:2001259 | positive regulation of cation channel activity | 0.011040008 | 44 | 8 |
| GO:1903428 | positive regulation of reactive oxygen species biosynthetic process | 0.01119157 | 22 | 4 |
| GO:0043486 | histone exchange | 0.011332405 | 23 | 5 |
| GO:2000026 | regulation of multicellular organismal development | 0.011381497 | 83 | 10 |
| GO:0060562 | epithelial tube morphogenesis | 0.011412479 | 32 | 6 |
| GO:0045200 | establishment of neuroblast polarity | 0.012094773 | 26 | 4 |
| GO:0010458 | exit from mitosis | 0.01224387 | 39 | 6 |
| GO:0032088 | negative regulation of NF-kappaB transcription factor activity | 0.012408966 | 154 | 11 |
| GO:0006607 | NLS-bearing protein import into nucleus | 0.012946646 | 43 | 5 |
| GO:0006302 | double-strand break repair | 0.013033434 | 98 | 8 |
| GO:0046939 | nucleotide phosphorylation | 0.013283862 | 42 | 5 |
| GO:0010288 | response to lead ion | 0.013649056 | 62 | 6 |
| GO:0006342 | chromatin silencing | 0.013851989 | 167 | 14 |
| GO:0043113 | receptor clustering | 0.014172273 | 76 | 10 |
| GO:0019915 | lipid storage | 0.014244936 | 162 | 12 |
| GO:0007052 | mitotic spindle organization | 0.014551215 | 181 | 13 |
| GO:1902476 | chloride transmembrane transport | 0.014639653 | 138 | 10 |
| GO:0048821 | erythrocyte development | 0.01485592 | 57 | 8 |
| GO:0050821 | protein stabilization | 0.015980449 | 357 | 21 |
| GO:0010634 | positive regulation of epithelial cell migration | 0.015985433 | 95 | 9 |
| GO:0009299 | mRNA transcription | 0.016102279 | 27 | 4 |
| GO:0014894 | response to denervation involved in regulation of muscle adaptation | 0.016185392 | 23 | 5 |
| GO:0030948 | negative regulation of vascular endothelial growth factor receptor signaling pathway | 0.016458607 | 27 | 5 |
| GO:0001932 | regulation of protein phosphorylation | 0.016551667 | 58 | 10 |
| GO:0008654 | phospholipid biosynthetic process | 0.016566401 | 35 | 5 |
| GO:0031345 | negative regulation of cell projection organization | 0.017073709 | 20 | 4 |
| GO:0070555 | response to interleukin-1 | 0.017195444 | 80 | 8 |
| GO:0008104 | protein localization | 0.017735895 | 171 | 17 |
| GO:0071420 | cellular response to histamine | 0.017741007 | 36 | 4 |
| GO:0035159 | regulation of tube length, open tracheal system | 0.017817347 | 23 | 6 |
| GO:0001702 | gastrulation with mouth forming second | 0.018119819 | 90 | 12 |
| GO:0010960 | magnesium ion homeostasis | 0.018165753 | 35 | 7 |
| GO:0006651 | diacylglycerol biosynthetic process | 0.018213974 | 30 | 5 |
| GO:0038083 | peptidyl-tyrosine autophosphorylation | 0.01890937 | 64 | 7 |
| GO:0090314 | positive regulation of protein targeting to membrane | 0.019883905 | 69 | 8 |
| GO:0016573 | histone acetylation | 0.020072338 | 86 | 9 |
| GO:0007608 | sensory perception of smell | 0.020123816 | 124 | 10 |
| GO:0045454 | cell redox homeostasis | 0.020620634 | 102 | 11 |
| GO:0051932 | synaptic transmission, GABAergic | 0.020887749 | 97 | 9 |
| GO:0070317 | negative regulation of G0 to G1 transition | 0.020904017 | 20 | 4 |
| GO:0048172 | regulation of short-term neuronal synaptic plasticity | 0.021181586 | 79 | 9 |
| GO:0007095 | mitotic G2 DNA damage checkpoint | 0.021266996 | 130 | 9 |
| GO:0060235 | lens induction in camera-type eye | 0.021501175 | 21 | 4 |
| GO:0010310 | regulation of hydrogen peroxide metabolic process | 0.022132589 | 22 | 3 |
| GO:0090162 | establishment of epithelial cell polarity | 0.022172154 | 61 | 7 |
| GO:0097193 | intrinsic apoptotic signaling pathway | 0.022520883 | 128 | 8 |
| GO:0032147 | activation of protein kinase activity | 0.022674737 | 185 | 12 |
| GO:0045838 | positive regulation of membrane potential | 0.023160209 | 31 | 6 |
| GO:0007186 | G-protein coupled receptor signaling pathway | 0.023378589 | 347 | 24 |
| GO:0060024 | rhythmic synaptic transmission | 0.023523141 | 37 | 6 |
| GO:0060996 | dendritic spine development | 0.02387406 | 48 | 5 |
| GO:0090630 | activation of GTPase activity | 0.023951091 | 156 | 13 |
| GO:0043409 | negative regulation of MAPK cascade | 0.024244472 | 26 | 5 |
| GO:0034394 | protein localization to cell surface | 0.024291683 | 69 | 9 |
| GO:0071709 | membrane assembly | 0.024944882 | 20 | 7 |
| GO:0050729 | positive regulation of inflammatory response | 0.025255609 | 61 | 5 |
| GO:0007214 | gamma-aminobutyric acid signaling pathway | 0.025291239 | 65 | 7 |
| GO:0043030 | regulation of macrophage activation | 0.025349349 | 21 | 3 |
| GO:0007611 | learning or memory | 0.025399332 | 138 | 13 |
| GO:0007043 | cell-cell junction assembly | 0.025587908 | 37 | 5 |
| GO:0007596 | blood coagulation | 0.026378668 | 460 | 24 |
| GO:0001574 | ganglioside biosynthetic process | 0.026884891 | 28 | 3 |
| GO:0045921 | positive regulation of exocytosis | 0.027109055 | 90 | 8 |
| GO:0060397 | JAK-STAT cascade involved in growth hormone signaling pathway | 0.027703323 | 39 | 4 |
| GO:0050832 | defense response to fungus | 0.027784826 | 83 | 11 |
| GO:0021591 | ventricular system development | 0.027881717 | 65 | 10 |
| GO:0043982 | histone H4-K8 acetylation | 0.027881868 | 46 | 6 |
| GO:0006020 | inositol metabolic process | 0.027900196 | 26 | 4 |
| GO:0030728 | ovulation | 0.02875822 | 23 | 5 |
| GO:0071333 | cellular response to glucose stimulus | 0.028758386 | 168 | 11 |
| GO:0055085 | transmembrane transport | 0.029664196 | 313 | 17 |
| GO:0010592 | positive regulation of lamellipodium assembly | 0.029906142 | 76 | 6 |
| GO:0034122 | negative regulation of toll-like receptor signaling pathway | 0.03009698 | 26 | 3 |
| GO:0071158 | positive regulation of cell cycle arrest | 0.030418092 | 59 | 5 |
| GO:0042129 | regulation of T cell proliferation | 0.030608072 | 26 | 3 |
| GO:0032410 | negative regulation of transporter activity | 0.030627182 | 23 | 3 |
| GO:0060307 | regulation of ventricular cardiac muscle cell membrane repolarization | 0.030926704 | 45 | 8 |
| GO:0040013 | negative regulation of locomotion | 0.03097416 | 29 | 3 |
| GO:0030183 | B cell differentiation | 0.031510451 | 107 | 10 |
| GO:0032869 | cellular response to insulin stimulus | 0.031740473 | 248 | 15 |
| GO:0048666 | neuron development | 0.031937239 | 143 | 13 |
| GO:0008285 | negative regulation of cell proliferation | 0.032527749 | 855 | 47 |
| GO:0048009 | insulin-like growth factor receptor signaling pathway | 0.032571413 | 67 | 7 |
| GO:0000088 | mitotic prophase | 0.032664833 | 138 | 9 |
| GO:1903861 | positive regulation of dendrite extension | 0.032969993 | 69 | 9 |
| GO:0007420 | brain development | 0.034012426 | 572 | 32 |
| GO:0015914 | phospholipid transport | 0.034064257 | 60 | 5 |
| GO:0050796 | regulation of insulin secretion | 0.034293105 | 190 | 13 |
| GO:0043968 | histone H2A acetylation | 0.034779066 | 31 | 4 |
| GO:0007172 | signal complex assembly | 0.034794031 | 25 | 3 |
| GO:0046627 | negative regulation of insulin receptor signaling pathway | 0.034896506 | 105 | 8 |
| GO:0006468 | protein phosphorylation | 0.03552311 | 410 | 23 |
| GO:0033120 | positive regulation of RNA splicing | 0.035602524 | 53 | 5 |
| GO:0036336 | dendritic cell migration | 0.035627312 | 20 | 3 |
| GO:1901017 | negative regulation of potassium ion transmembrane transporter activity | 0.035784643 | 22 | 3 |
| GO:0050890 | cognition | 0.037377736 | 66 | 8 |
| GO:0006979 | response to oxidative stress | 0.037391681 | 251 | 14 |
| GO:1990090 | cellular response to nerve growth factor stimulus | 0.03748905 | 131 | 10 |
| GO:1901800 | positive regulation of proteasomal protein catabolic process | 0.037789283 | 41 | 4 |
| GO:0007617 | mating behavior | 0.038145761 | 42 | 4 |
| GO:0042157 | lipoprotein metabolic process | 0.039195242 | 33 | 6 |
| GO:0098910 | regulation of atrial cardiac muscle cell action potential | 0.039298313 | 20 | 7 |
| GO:0046580 | negative regulation of Ras protein signal transduction | 0.039379957 | 78 | 7 |
| GO:0050775 | positive regulation of dendrite morphogenesis | 0.039934217 | 80 | 8 |
| GO:0050882 | voluntary musculoskeletal movement | 0.039940986 | 20 | 5 |
| GO:0001881 | receptor recycling | 0.040266087 | 26 | 4 |
| GO:0055072 | iron ion homeostasis | 0.040884551 | 65 | 7 |
| GO:0035924 | cellular response to vascular endothelial growth factor stimulus | 0.040944957 | 77 | 7 |
| GO:0051387 | negative regulation of neurotrophin TRK receptor signaling pathway | 0.041025891 | 26 | 4 |
| GO:0031340 | positive regulation of vesicle fusion | 0.041064831 | 40 | 4 |
| GO:1903779 | regulation of cardiac conduction | 0.041186252 | 24 | 4 |
| GO:0021549 | cerebellum development | 0.041675869 | 178 | 12 |
| GO:0009791 | post-embryonic development | 0.041987081 | 377 | 28 |
| GO:0009628 | response to abiotic stimulus | 0.042114779 | 93 | 7 |
| GO:0032922 | circadian regulation of gene expression | 0.042946404 | 194 | 14 |
| GO:0030010 | establishment of cell polarity | 0.043382404 | 114 | 10 |
| GO:0086005 | ventricular cardiac muscle cell action potential | 0.044153368 | 51 | 9 |
| GO:0050727 | regulation of inflammatory response | 0.044379988 | 122 | 8 |
| GO:0048536 | spleen development | 0.044516128 | 104 | 10 |
| GO:0033148 | positive regulation of intracellular estrogen receptor signaling pathway | 0.044548004 | 48 | 7 |
| GO:2001020 | regulation of response to DNA damage stimulus | 0.044873128 | 35 | 5 |
| GO:0006779 | porphyrin-containing compound biosynthetic process | 0.044948131 | 28 | 7 |
| GO:0051963 | regulation of synapse assembly | 0.045170262 | 54 | 7 |
| GO:0006972 | hyperosmotic response | 0.045438982 | 76 | 5 |
| GO:0007252 | I-kappaB phosphorylation | 0.046359992 | 31 | 4 |
| GO:0001778 | plasma membrane repair | 0.046391858 | 20 | 4 |
| GO:0045820 | negative regulation of glycolytic process | 0.046718094 | 42 | 4 |
| GO:0042771 | intrinsic apoptotic signaling pathway in response to DNA damage by p53 class mediator | 0.046746549 | 74 | 7 |
| GO:1902600 | hydrogen ion transmembrane transport | 0.047395413 | 90 | 5 |
| GO:0031047 | gene silencing by RNA | 0.047534006 | 46 | 4 |
| GO:0014066 | regulation of phosphatidylinositol 3-kinase signaling | 0.047712663 | 22 | 3 |
| GO:0090307 | mitotic spindle assembly | 0.047937451 | 113 | 7 |
| GO:0071374 | cellular response to parathyroid hormone stimulus | 0.048597204 | 33 | 4 |
| GO:0070102 | interleukin-6-mediated signaling pathway | 0.049228778 | 24 | 3 |
| GO:0035162 | embryonic hemopoiesis | 0.049881259 | 84 | 9 |
| GO:0032482 | Rab protein signal transduction | 0.049913583 | 141 | 7 |
| GO:0036010 | protein localization to endosome | 0.049996504 | 40 | 4 |

**Table S6.** Gene Ontology (GO) categories with a significant (*p* < 0.05) overrepresentation of downregulated genes in Atlantic salmon juveniles subjected to 8 weeks of exercise, compared to unexercised control.

| GO ID | GO term | *p* value | No. genes in GO category | No. genes downregulated in GO category |
| --- | --- | --- | --- | --- |
| GO:0009792 | embryo development ending in birth or egg hatching | 8.48E-16 | 995 | 112 |
| GO:0040035 | hermaphrodite genitalia development | 2.24E-10 | 401 | 52 |
| GO:0000398 | mRNA splicing, via spliceosome | 5.41E-10 | 319 | 44 |
| GO:0002119 | nematode larval development | 2.51E-09 | 812 | 83 |
| GO:0016032 | viral process | 3.88E-09 | 647 | 60 |
| GO:0040011 | locomotion | 6.52E-09 | 439 | 45 |
| GO:0006369 | termination of RNA polymerase II transcription | 3.44E-08 | 140 | 27 |
| GO:0048255 | mRNA stabilization | 3.06E-07 | 43 | 11 |
| GO:0008340 | determination of adult lifespan | 7.05E-07 | 356 | 36 |
| GO:0031124 | mRNA 3'-end processing | 9.06E-07 | 85 | 20 |
| GO:0040020 | regulation of meiotic nuclear division | 6.65E-06 | 83 | 13 |
| GO:0006120 | mitochondrial electron transport, NADH to ubiquinone | 7.82E-06 | 58 | 18 |
| GO:0000381 | regulation of alternative mRNA splicing, via spliceosome | 8.00E-06 | 175 | 21 |
| GO:0007476 | imaginal disc-derived wing morphogenesis | 9.22E-06 | 115 | 13 |
| GO:0022904 | respiratory electron transport chain | 1.03E-05 | 112 | 24 |
| GO:0048025 | negative regulation of mRNA splicing, via spliceosome | 1.05E-05 | 107 | 17 |
| GO:0006898 | receptor-mediated endocytosis | 1.18E-05 | 442 | 37 |
| GO:0061025 | membrane fusion | 1.18E-05 | 75 | 14 |
| GO:0000422 | autophagy of mitochondrion | 1.54E-05 | 79 | 14 |
| GO:0010171 | body morphogenesis | 1.97E-05 | 241 | 29 |
| GO:1902600 | hydrogen ion transmembrane transport | 2.03E-05 | 90 | 18 |
| GO:0008286 | insulin receptor signaling pathway | 2.05E-05 | 654 | 43 |
| GO:0006406 | mRNA export from nucleus | 2.19E-05 | 131 | 19 |
| GO:0000394 | RNA splicing, via endonucleolytic cleavage and ligation | 2.29E-05 | 29 | 10 |
| GO:0015991 | ATP hydrolysis coupled proton transport | 2.43E-05 | 53 | 11 |
| GO:0018991 | oviposition | 4.19E-05 | 124 | 15 |
| GO:0042776 | mitochondrial ATP synthesis coupled proton transport | 4.47E-05 | 31 | 11 |
| GO:0006915 | apoptotic process | 6.15E-05 | 913 | 64 |
| GO:0009409 | response to cold | 8.53E-05 | 122 | 15 |
| GO:0006995 | cellular response to nitrogen starvation | 8.60E-05 | 35 | 9 |
| GO:0035308 | negative regulation of protein dephosphorylation | 0.00012 | 24 | 6 |
| GO:0034063 | stress granule assembly | 0.00013 | 39 | 7 |
| GO:0007052 | mitotic spindle organization | 0.00014 | 181 | 18 |
| GO:0002223 | stimulatory C-type lectin receptor signaling pathway | 0.00016 | 256 | 28 |
| GO:0061001 | regulation of dendritic spine morphogenesis | 0.00017 | 62 | 6 |
| GO:0070534 | protein K63-linked ubiquitination | 0.00017 | 118 | 15 |
| GO:0043086 | negative regulation of catalytic activity | 0.00018 | 156 | 16 |
| GO:0000413 | protein peptidyl-prolyl isomerization | 0.00019 | 61 | 12 |
| GO:0035021 | negative regulation of Rac protein signal transduction | 0.00019 | 23 | 6 |
| GO:0040024 | dauer larval development | 0.00022 | 32 | 6 |
| GO:0040025 | vulval development | 0.00023 | 48 | 9 |
| GO:0007294 | germarium-derived oocyte fate determination | 0.00024 | 23 | 5 |
| GO:0070265 | necrotic cell death | 0.00026 | 23 | 5 |
| GO:0070979 | protein K11-linked ubiquitination | 0.00039 | 69 | 12 |
| GO:1900740 | positive regulation of protein insertion into mitochondrial membrane involved in apoptotic signaling pathway | 0.00046 | 74 | 10 |
| GO:0070936 | protein K48-linked ubiquitination | 0.00046 | 142 | 14 |
| GO:0051437 | positive regulation of ubiquitin-protein ligase activity involved in regulation of mitotic cell cycle transition | 0.00049 | 162 | 24 |
| GO:0006521 | regulation of cellular amino acid metabolic process | 0.00050 | 102 | 18 |
| GO:0048208 | COPII vesicle coating | 0.00053 | 38 | 6 |
| GO:0035196 | production of miRNAs involved in gene silencing by miRNA | 0.00065 | 27 | 6 |
| GO:0034975 | protein folding in endoplasmic reticulum | 0.00066 | 40 | 7 |
| GO:0038061 | NIK/NF-kappaB signaling | 0.00070 | 122 | 19 |
| GO:0000723 | telomere maintenance | 0.00074 | 48 | 8 |
| GO:0006367 | transcription initiation from RNA polymerase II promoter | 0.00081 | 478 | 32 |
| GO:0017148 | negative regulation of translation | 0.00082 | 191 | 15 |
| GO:0090382 | phagosome maturation | 0.00087 | 50 | 8 |
| GO:0030433 | ubiquitin-dependent ERAD pathway | 0.00093 | 115 | 13 |
| GO:0031145 | anaphase-promoting complex-dependent catabolic process | 0.00095 | 172 | 24 |
| GO:0010940 | positive regulation of necrotic cell death | 0.00098 | 28 | 5 |
| GO:0040027 | negative regulation of vulval development | 0.00100 | 137 | 17 |
| GO:0006626 | protein targeting to mitochondrion | 0.00103 | 92 | 14 |
| GO:0044281 | small molecule metabolic process | 0.00108 | 760 | 47 |
| GO:0035459 | cargo loading into vesicle | 0.00108 | 23 | 4 |
| GO:0045116 | protein neddylation | 0.00117 | 28 | 7 |
| GO:0050830 | defense response to Gram-positive bacterium | 0.00118 | 80 | 10 |
| GO:0060212 | negative regulation of nuclear-transcribed mRNA poly(A) tail shortening | 0.00125 | 24 | 6 |
| GO:0060315 | negative regulation of ryanodine-sensitive calcium-release channel activity | 0.00135 | 32 | 7 |
| GO:0051701 | interaction with host | 0.00136 | 51 | 8 |
| GO:0002479 | antigen processing and presentation of exogenous peptide antigen via MHC class I, TAP-dependent | 0.00138 | 137 | 20 |
| GO:0009965 | leaf morphogenesis | 0.00139 | 32 | 7 |
| GO:0000387 | spliceosomal snRNP assembly | 0.00140 | 51 | 11 |
| GO:0061077 | chaperone-mediated protein folding | 0.00166 | 86 | 11 |
| GO:0045446 | endothelial cell differentiation | 0.00166 | 34 | 5 |
| GO:0009411 | response to UV | 0.00172 | 95 | 9 |
| GO:0006997 | nucleus organization | 0.00172 | 66 | 9 |
| GO:0042981 | regulation of apoptotic process | 0.00182 | 245 | 14 |
| GO:0042921 | glucocorticoid receptor signaling pathway | 0.00184 | 41 | 5 |
| GO:0033209 | tumor necrosis factor-mediated signaling pathway | 0.00185 | 179 | 21 |
| GO:0016036 | cellular response to phosphate starvation | 0.00188 | 20 | 4 |
| GO:0022008 | neurogenesis | 0.00191 | 291 | 25 |
| GO:0000395 | mRNA 5'-splice site recognition | 0.00203 | 26 | 6 |
| GO:0006879 | cellular iron ion homeostasis | 0.00220 | 134 | 14 |
| GO:0033120 | positive regulation of RNA splicing | 0.00223 | 53 | 6 |
| GO:0048680 | positive regulation of axon regeneration | 0.00226 | 23 | 5 |
| GO:0006091 | generation of precursor metabolites and energy | 0.00234 | 41 | 7 |
| GO:0007140 | male meiotic nuclear division | 0.00236 | 54 | 7 |
| GO:0006337 | nucleosome disassembly | 0.00242 | 51 | 5 |
| GO:0022417 | protein maturation by protein folding | 0.00269 | 31 | 6 |
| GO:0030836 | positive regulation of actin filament depolymerization | 0.00271 | 22 | 5 |
| GO:0097167 | circadian regulation of translation | 0.00280 | 24 | 4 |
| GO:0030042 | actin filament depolymerization | 0.00287 | 26 | 4 |
| GO:0050832 | defense response to fungus | 0.00290 | 83 | 8 |
| GO:0035067 | negative regulation of histone acetylation | 0.00290 | 22 | 4 |
| GO:0071901 | negative regulation of protein serine/threonine kinase activity | 0.00298 | 46 | 5 |
| GO:0006368 | transcription elongation from RNA polymerase II promoter | 0.00315 | 131 | 14 |
| GO:0009793 | embryo development ending in seed dormancy | 0.00326 | 99 | 12 |
| GO:0045727 | positive regulation of translation | 0.00329 | 230 | 17 |
| GO:0035071 | salivary gland cell autophagic cell death | 0.00335 | 58 | 10 |
| GO:0010467 | gene expression | 0.00347 | 292 | 22 |
| GO:0021762 | substantia nigra development | 0.00349 | 174 | 17 |
| GO:0006605 | protein targeting | 0.00399 | 124 | 9 |
| GO:0044723 | NA | 0.00420 | 31 | 5 |
| GO:0007095 | mitotic G2 DNA damage checkpoint | 0.00431 | 130 | 12 |
| GO:0000226 | microtubule cytoskeleton organization | 0.00437 | 174 | 11 |
| GO:0007280 | pole cell migration | 0.00451 | 23 | 3 |
| GO:0006334 | nucleosome assembly | 0.00451 | 90 | 9 |
| GO:0006336 | DNA replication-independent nucleosome assembly | 0.00465 | 26 | 5 |
| GO:0000245 | spliceosomal complex assembly | 0.00476 | 81 | 11 |
| GO:0048205 | COPI coating of Golgi vesicle | 0.00497 | 38 | 6 |
| GO:0051436 | negative regulation of ubiquitin-protein ligase activity involved in mitotic cell cycle | 0.00500 | 113 | 17 |
| GO:0002576 | platelet degranulation | 0.00510 | 137 | 12 |
| GO:0050434 | positive regulation of viral transcription | 0.00548 | 77 | 10 |
| GO:0061158 | 3'-UTR-mediated mRNA destabilization | 0.00559 | 28 | 4 |
| GO:0097193 | intrinsic apoptotic signaling pathway | 0.00562 | 128 | 11 |
| GO:0070124 | mitochondrial translational initiation | 0.00581 | 114 | 18 |
| GO:0032940 | secretion by cell | 0.00588 | 90 | 10 |
| GO:0000165 | MAPK cascade | 0.00597 | 386 | 23 |
| GO:0043162 | ubiquitin-dependent protein catabolic process via the multivesicular body sorting pathway | 0.00612 | 58 | 7 |
| GO:0035329 | hippo signaling | 0.00612 | 61 | 5 |
| GO:0007281 | germ cell development | 0.00620 | 164 | 13 |
| GO:0071392 | cellular response to estradiol stimulus | 0.00631 | 105 | 8 |
| GO:0030431 | sleep | 0.00647 | 75 | 7 |
| GO:0070126 | mitochondrial translational termination | 0.00656 | 116 | 18 |
| GO:0016236 | macroautophagy | 0.00661 | 72 | 8 |
| GO:0000183 | chromatin silencing at rDNA | 0.00698 | 37 | 5 |
| GO:0009408 | response to heat | 0.00715 | 212 | 16 |
| GO:0008103 | oocyte microtubule cytoskeleton polarization | 0.00723 | 44 | 5 |
| GO:0030182 | neuron differentiation | 0.00737 | 152 | 11 |
| GO:0044804 | autophagy of nucleus | 0.00750 | 35 | 6 |
| GO:0030866 | cortical actin cytoskeleton organization | 0.00791 | 105 | 7 |
| GO:0021549 | cerebellum development | 0.00793 | 178 | 11 |
| GO:0007265 | Ras protein signal transduction | 0.00853 | 460 | 27 |
| GO:0007291 | sperm individualization | 0.00923 | 46 | 6 |
| GO:0000082 | G1/S transition of mitotic cell cycle | 0.00929 | 360 | 27 |
| GO:0007088 | regulation of mitotic nuclear division | 0.00932 | 89 | 7 |
| GO:0042692 | muscle cell differentiation | 0.00986 | 75 | 6 |
| GO:0010388 | NA | 0.01007 | 39 | 7 |
| GO:0006977 | DNA damage response, signal transduction by p53 class mediator resulting in cell cycle arrest | 0.01010 | 149 | 18 |
| GO:0033572 | transferrin transport | 0.01014 | 86 | 8 |
| GO:0000090 | mitotic anaphase | 0.01057 | 327 | 27 |
| GO:0043388 | positive regulation of DNA binding | 0.01090 | 90 | 9 |
| GO:0010228 | vegetative to reproductive phase transition of meristem | 0.01092 | 30 | 5 |
| GO:0038095 | Fc-epsilon receptor signaling pathway | 0.01092 | 595 | 31 |
| GO:1901800 | positive regulation of proteasomal protein catabolic process | 0.01105 | 41 | 5 |
| GO:0048190 | wing disc dorsal/ventral pattern formation | 0.01146 | 31 | 3 |
| GO:0070125 | mitochondrial translational elongation | 0.01152 | 120 | 18 |
| GO:0046716 | muscle cell cellular homeostasis | 0.01183 | 79 | 7 |
| GO:0006376 | mRNA splice site selection | 0.01186 | 58 | 7 |
| GO:0071230 | cellular response to amino acid stimulus | 0.01209 | 130 | 8 |
| GO:0045792 | negative regulation of cell size | 0.01212 | 52 | 5 |
| GO:0048013 | ephrin receptor signaling pathway | 0.01246 | 316 | 15 |
| GO:0000003 | reproduction | 0.01273 | 541 | 39 |
| GO:0051683 | establishment of Golgi localization | 0.01296 | 39 | 5 |
| GO:0007019 | microtubule depolymerization | 0.01338 | 31 | 5 |
| GO:0061484 | hematopoietic stem cell homeostasis | 0.01344 | 26 | 4 |
| GO:0021766 | hippocampus development | 0.01350 | 231 | 10 |
| GO:1901224 | positive regulation of NIK/NF-kappaB signaling | 0.01354 | 31 | 3 |
| GO:0008039 | synaptic target recognition | 0.01371 | 21 | 4 |
| GO:0071732 | cellular response to nitric oxide | 0.01393 | 43 | 4 |
| GO:0006623 | protein targeting to vacuole | 0.01412 | 54 | 7 |
| GO:0007422 | peripheral nervous system development | 0.01427 | 151 | 9 |
| GO:0086013 | membrane repolarization during cardiac muscle cell action potential | 0.01464 | 31 | 3 |
| GO:1901016 | regulation of potassium ion transmembrane transporter activity | 0.01490 | 26 | 3 |
| GO:0042787 | protein ubiquitination involved in ubiquitin-dependent protein catabolic process | 0.01497 | 216 | 13 |
| GO:0018996 | molting cycle, collagen and cuticulin-based cuticle | 0.01513 | 127 | 13 |
| GO:0034660 | ncRNA metabolic process | 0.01548 | 42 | 8 |
| GO:0000045 | autophagosome assembly | 0.01552 | 87 | 8 |
| GO:0043206 | NA | 0.01648 | 23 | 3 |
| GO:0010881 | regulation of cardiac muscle contraction by regulation of the release of sequestered calcium ion | 0.01659 | 78 | 6 |
| GO:0009737 | response to abscisic acid | 0.01679 | 49 | 6 |
| GO:0090090 | negative regulation of canonical Wnt signaling pathway | 0.01680 | 396 | 22 |
| GO:0032516 | positive regulation of phosphoprotein phosphatase activity | 0.01699 | 38 | 4 |
| GO:0010976 | positive regulation of neuron projection development | 0.01708 | 356 | 16 |
| GO:0045087 | innate immune response | 0.01709 | 1119 | 51 |
| GO:0043087 | regulation of GTPase activity | 0.01748 | 101 | 6 |
| GO:0006509 | membrane protein ectodomain proteolysis | 0.01757 | 53 | 5 |
| GO:0090307 | mitotic spindle assembly | 0.01759 | 113 | 10 |
| GO:2001243 | negative regulation of intrinsic apoptotic signaling pathway | 0.01795 | 60 | 6 |
| GO:0060306 | regulation of membrane repolarization | 0.01800 | 23 | 3 |
| GO:0033523 | histone H2B ubiquitination | 0.01805 | 29 | 5 |
| GO:0055007 | cardiac muscle cell differentiation | 0.01816 | 75 | 4 |
| GO:0045132 | meiotic chromosome segregation | 0.01858 | 45 | 5 |
| GO:0034047 | NA | 0.01900 | 44 | 5 |
| GO:0072499 | photoreceptor cell axon guidance | 0.01910 | 25 | 3 |
| GO:0072593 | reactive oxygen species metabolic process | 0.01926 | 69 | 7 |
| GO:0034454 | microtubule anchoring at centrosome | 0.01934 | 20 | 4 |
| GO:0006661 | phosphatidylinositol biosynthetic process | 0.01956 | 112 | 7 |
| GO:0036099 | female germ-line stem cell population maintenance | 0.01979 | 35 | 6 |
| GO:0051489 | regulation of filopodium assembly | 0.01990 | 73 | 6 |
| GO:0016239 | positive regulation of macroautophagy | 0.01998 | 35 | 3 |
| GO:0016568 | NA | 0.02007 | 126 | 8 |
| GO:0010390 | histone monoubiquitination | 0.02044 | 44 | 5 |
| GO:0044458 | motile cilium assembly | 0.02053 | 29 | 4 |
| GO:0010459 | negative regulation of heart rate | 0.02073 | 29 | 4 |
| GO:0048812 | neuron projection morphogenesis | 0.02126 | 162 | 9 |
| GO:0043281 | regulation of cysteine-type endopeptidase activity involved in apoptotic process | 0.02128 | 64 | 4 |
| GO:0045292 | mRNA cis splicing, via spliceosome | 0.02128 | 28 | 5 |
| GO:0007183 | SMAD protein complex assembly | 0.02156 | 23 | 3 |
| GO:0019886 | antigen processing and presentation of exogenous peptide antigen via MHC class II | 0.02222 | 208 | 11 |
| GO:0031987 | locomotion involved in locomotory behavior | 0.02227 | 36 | 3 |
| GO:0009853 | photorespiration | 0.02228 | 45 | 7 |
| GO:0048387 | negative regulation of retinoic acid receptor signaling pathway | 0.02258 | 30 | 3 |
| GO:0043652 | engulfment of apoptotic cell | 0.02282 | 51 | 6 |
| GO:0006465 | signal peptide processing | 0.02315 | 24 | 4 |
| GO:0000132 | establishment of mitotic spindle orientation | 0.02320 | 101 | 7 |
| GO:0006412 | translation | 0.02371 | 151 | 13 |
| GO:0048477 | oogenesis | 0.02387 | 187 | 13 |
| GO:0007288 | sperm axoneme assembly | 0.02545 | 26 | 3 |
| GO:0030177 | positive regulation of Wnt signaling pathway | 0.02552 | 147 | 7 |
| GO:0043488 | regulation of mRNA stability | 0.02553 | 47 | 5 |
| GO:0044708 | NA | 0.02586 | 32 | 3 |
| GO:0007369 | gastrulation | 0.02593 | 79 | 7 |
| GO:0042048 | olfactory behavior | 0.02598 | 52 | 3 |
| GO:0000084 | mitotic S phase | 0.02608 | 200 | 19 |
| GO:1900028 | negative regulation of ruffle assembly | 0.02781 | 26 | 3 |
| GO:1901841 | regulation of high voltage-gated calcium channel activity | 0.02790 | 30 | 4 |
| GO:0001731 | formation of translation preinitiation complex | 0.02807 | 45 | 7 |
| GO:0007179 | transforming growth factor beta receptor signaling pathway | 0.02835 | 374 | 18 |
| GO:0006417 | regulation of translation | 0.02845 | 123 | 10 |
| GO:0046329 | negative regulation of JNK cascade | 0.02944 | 98 | 5 |
| GO:0009407 | toxin catabolic process | 0.02950 | 32 | 6 |
| GO:0035046 | pronuclear migration | 0.02964 | 51 | 6 |
| GO:0031000 | response to caffeine | 0.02984 | 40 | 4 |
| GO:0006783 | heme biosynthetic process | 0.03021 | 31 | 5 |
| GO:0002322 | B cell proliferation involved in immune response | 0.03035 | 22 | 3 |
| GO:0061179 | negative regulation of insulin secretion involved in cellular response to glucose stimulus | 0.03100 | 35 | 4 |
| GO:1901673 | regulation of mitotic spindle assembly | 0.03106 | 28 | 4 |
| GO:0042098 | T cell proliferation | 0.03212 | 54 | 5 |
| GO:0043951 | negative regulation of cAMP-mediated signaling | 0.03217 | 38 | 4 |
| GO:0006734 | NADH metabolic process | 0.03245 | 26 | 4 |
| GO:2000300 | regulation of synaptic vesicle exocytosis | 0.03245 | 25 | 3 |
| GO:0002168 | instar larval development | 0.03279 | 24 | 3 |
| GO:0051280 | negative regulation of release of sequestered calcium ion into cytosol | 0.03292 | 21 | 3 |
| GO:0051775 | response to redox state | 0.03326 | 34 | 4 |
| GO:0002407 | dendritic cell chemotaxis | 0.03338 | 22 | 3 |
| GO:0008582 | regulation of synaptic growth at neuromuscular junction | 0.03376 | 39 | 4 |
| GO:0030307 | positive regulation of cell growth | 0.03471 | 225 | 13 |
| GO:0000724 | double-strand break repair via homologous recombination | 0.03500 | 88 | 8 |
| GO:0032410 | negative regulation of transporter activity | 0.03565 | 23 | 3 |
| GO:0006344 | maintenance of chromatin silencing | 0.03584 | 24 | 3 |
| GO:0033554 | cellular response to stress | 0.03818 | 205 | 12 |
| GO:0033227 | dsRNA transport | 0.03860 | 38 | 4 |
| GO:0000186 | activation of MAPKK activity | 0.03932 | 452 | 24 |
| GO:0031115 | negative regulation of microtubule polymerization | 0.03944 | 59 | 5 |
| GO:0045947 | negative regulation of translational initiation | 0.03946 | 44 | 4 |
| GO:0042407 | cristae formation | 0.03966 | 27 | 4 |
| GO:0010172 | embryonic body morphogenesis | 0.03984 | 55 | 4 |
| GO:0043044 | ATP-dependent chromatin remodeling | 0.04026 | 86 | 5 |
| GO:0042326 | negative regulation of phosphorylation | 0.04093 | 38 | 4 |
| GO:0016477 | cell migration | 0.04093 | 491 | 19 |
| GO:0032007 | negative regulation of TOR signaling | 0.04105 | 77 | 5 |
| GO:0010633 | negative regulation of epithelial cell migration | 0.04112 | 63 | 4 |
| GO:0035584 | calcium-mediated signaling using intracellular calcium source | 0.04142 | 65 | 4 |
| GO:0007346 | regulation of mitotic cell cycle | 0.04173 | 132 | 7 |
| GO:0008625 | extrinsic apoptotic signaling pathway via death domain receptors | 0.04241 | 76 | 5 |
| GO:0008407 | chaeta morphogenesis | 0.04248 | 36 | 4 |
| GO:0048010 | vascular endothelial growth factor receptor signaling pathway | 0.04316 | 610 | 28 |
| GO:0051224 | negative regulation of protein transport | 0.04331 | 24 | 3 |
| GO:0006913 | nucleocytoplasmic transport | 0.04353 | 151 | 10 |
| GO:0048793 | pronephros development | 0.04368 | 75 | 6 |
| GO:0045165 | cell fate commitment | 0.04450 | 75 | 4 |
| GO:2000778 | positive regulation of interleukin-6 secretion | 0.04456 | 26 | 4 |
| GO:0001649 | osteoblast differentiation | 0.04471 | 257 | 16 |
| GO:0032008 | positive regulation of TOR signaling | 0.04500 | 48 | 5 |
| GO:0017145 | stem cell division | 0.04546 | 21 | 2 |
| GO:0043123 | positive regulation of I-kappaB kinase/NF-kappaB signaling | 0.04574 | 298 | 17 |
| GO:0008156 | negative regulation of DNA replication | 0.04690 | 37 | 4 |
| GO:0031398 | positive regulation of protein ubiquitination | 0.04733 | 119 | 7 |
| GO:0043983 | histone H4-K12 acetylation | 0.04735 | 33 | 3 |
| GO:0043537 | negative regulation of blood vessel endothelial cell migration | 0.04819 | 33 | 4 |
| GO:0006939 | smooth muscle contraction | 0.04858 | 50 | 3 |
